# Assembling membraneless organelles from de novo designed proteins

**DOI:** 10.1038/s41557-023-01321-y

**Published:** 2023-09-14

**Authors:** Alexander T. Hilditch, Andrey Romanyuk, Stephen J. Cross, Richard Obexer, Jennifer J. McManus, Derek N. Woolfson

**Affiliations:** 1 School of Chemistry, University of Bristol, Bristol, UK; 2 School of Biochemistry, University of Bristol, Bristol, UK; 3 Max Planck-Bristol Centre for Minimal Biology, University of Bristol, Bristol, UK; 4 Wolfson Bioimaging Facility, University of Bristol, Bristol, UK; 5 HH Wills Physics Laboratory, School of Physics, University of Bristol, Bristol, UK; 6 Bristol BioDesign Institute, School of Chemistry, University of Bristol, Bristol, UK; 7 Present Address: Department of Chemistry, Manchester Institute of Biotechnology, University of Manchester, Manchester, UK

**Keywords:** Synthetic biology, Synthetic biology

## Abstract

Recent advances in de novo protein design have delivered a diversity of discrete de novo protein structures and complexes. A new challenge for the field is to use these designs directly in cells to intervene in biological processes and augment natural systems. The bottom-up design of self-assembled objects such as microcompartments and membraneless organelles is one such challenge. Here we describe the design of genetically encoded polypeptides that form membraneless organelles in *Escherichia coli*. To do this, we combine de novo α-helical sequences, intrinsically disordered linkers and client proteins in single-polypeptide constructs. We tailor the properties of the helical regions to shift protein assembly from arrested assemblies to dynamic condensates. The designs are characterized in cells and in vitro using biophysical methods and soft-matter physics. Finally, we use the designed polypeptide to co-compartmentalize a functional enzyme pair in *E. coli*, improving product formation close to the theoretical limit.

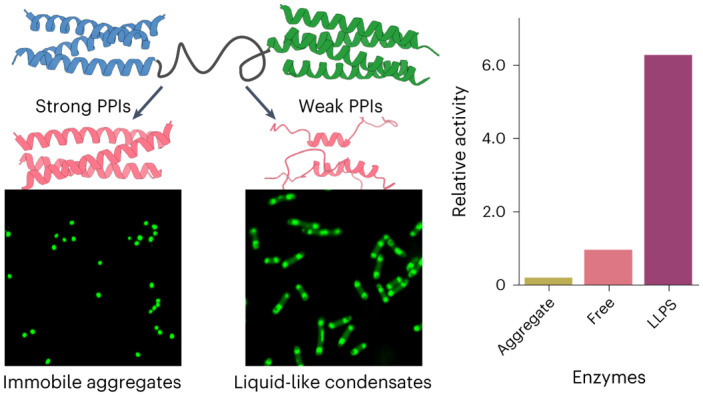

## Abstract (web summary)

Phase separation is being revealed as important in many biological processes. Most attempts to mimic and deconstruct this use engineered natural proteins. Now it is shown that de novo proteins can be designed from first principles to undergo liquid–liquid phase separation in cells, with the potential to organize multi-enzyme pathways.

## Main

**Fig. 1:**
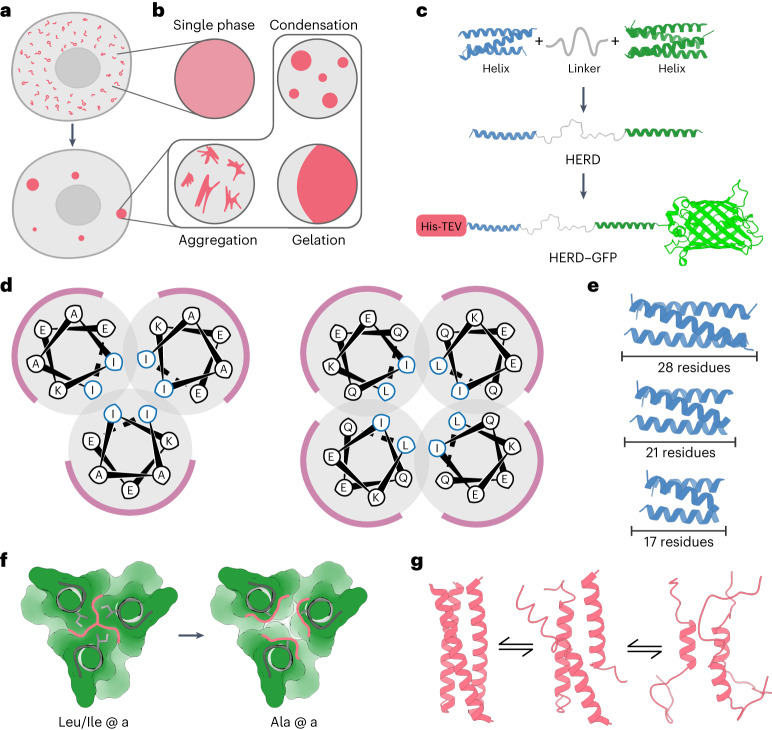
Design and assembly of de novo polypeptides for biomolecular condensation. **a**, Cartoon for membraneless-organelle formation in cells, that is, protein condensation leading to the formation of de-mixed droplets. **b**, Protein solutions can form a single phase, or phase-separated systems including condensates, aggregates and gels. **c**, HERD design strategy for phase separation by concatenation of de novo CCs. **d**, Helical wheels of the heptad (seven-residue) repeats for trimeric (left) and tetrameric (right) CCs with hydrophobic interface residues in blue and solvent-exposed residues in black. **e**–**g**, Weakening of PPIs by truncating the helical CC lengths (**e**), disrupting packing in the hydrophobic core through Ile/Leu (left) to Ala (right) mutations to the *a* position in the *abcdefg* heptad repeat (**f**) and reducing helical propensity by replacing surface residues to those with a low helical propensities (**g**).

The presence of dynamic cellular compartments known as membraneless organelles (MLOs) has been known for some time^1,2^. However, the widespread occurrence and utility of the phenomenon in biological systems, and specifically within cells have only recently become apparent^3,4^. Biomolecular condensates can take diverse forms including: amorphous aggregates, viscous liquids and gels, compartments formed by liquid–liquid phase separation (LLPS), and complex coacervates formed by protein–nucleic acid interactions (Fig. 1a,b)^5,6^. Each mode of condensation has different physical properties, and therefore the specific organization of macromolecules within the condensate has functional consequences^7,8^. LLPS is of particular interest because it can lead to highly dynamic and reversible cellular compartments that can respond to internal or external stimuli^9^. LLPS occurs when soluble macromolecules reversibly separate into de-mixed liquid phases, leaving one richer in the macromolecules than the other^10^. LLPS creates dense macromolecular phases that can accommodate diverse clients at high local concentrations, while permitting small molecules, proteins and nucleic acids to diffuse between the organelle and its surroundings^11^.

The ubiquity and utility of LLPS and MLOs in biology has brought the phenomenon to the attention of synthetic biologists^12^. Their aim is clear: to build artificial phase-separated compartments within cells to provide new and engineerable routes to functional MLOs. Indeed, artificially induced protein condensation has been demonstrated by exploiting the properties of natural and engineered intrinsically disordered proteins^13–18^. As an alternative to using natural sequences, here we demonstrate the bottom-up de novo design of polypeptides to promote protein condensation in cells. This uses weak, short-ranged attractive protein–protein interactions (PPIs) to drive condensation. The proteins present a programmable platform orthogonal to the host proteome, with the potential to expand the capabilities of LLPS and MLOs in synthetic biology.

With the coming of age of de novo protein design^19^, researchers are now exploring the construction of protein assemblies that interface with and augment biology^20^. These include small self-assembled polypeptide-based objects (origamis)^21^; fibrous materials for organizing proteins and reporting on cellular events^22–24^; and the rational and computational design of large peptide- and protein-based cages for cell delivery^25^. The design of peptides or proteins for LLPS would explore uncharted design space by exploiting weak and structurally less-well-defined interactions. De novo proteins, such as our own set of de novo α-helical coiled coils (CCs)^26,27^, are good starting points for creating new assemblies^28^ due to their defined interactions and orthogonality to natural proteomes^29^. Indeed, generally, helical motifs are recognized as key oligomerization motifs in protein condensates^30,31^. CCs provide high valencies encoded in short helical sequences. Therefore, they have the potential to mimic the high-valency interactions of natural proteins that undergo LLPS^32^. Further, our understanding of sequence-to-structure relationships for CCs presents a tractable route towards engineering the PPIs that they make and, thus, their collective solution behaviour^33,34^.

In this Article, we present the de novo design and characterization of genetically encoded polypeptides that form dynamic droplets under physiological conditions in *Escherichia coli*. We start by concatenating multivalent de novo CCs. The properties of these helical regions are then tuned to direct weakened PPIs leading to condensation consistent with LLPS. Droplet formation is reversible with an upper critical solution temperature. The PPIs are weakly attractive with an interaction parameter (diffusivity), *k*_D_, consistent with natural proteins that undergo LLPS. Interestingly, LLPS is triggerable within a physiologically accessible temperature range, which we exploit to modulate the material properties of droplets directly within bacteria. Finally, we demonstrate the potential of our de novo polypeptide system to generate functional organelle-like compartments in *E. coli* by co-compartmentalizing different client proteins including two enzymes to produce indigo in the host cells.

## Results and discussion

### De novo design delivers subcellular protein condensates

To generate a modular polypeptide that promotes biomolecular condensation in cells, we focused on emulating high-valency PPIs of the sticker-spacer model for natural condensates and hydrogels^35,36^. To do this, we concatenated two α-helical CCs via a flexible linker to create helical-repeat domains (HERD; Fig. 1c). Specifically, we used extant de novo trimeric (CC-Tri)^26^ and tetrameric (CC-Tet)^37^ CCs as the stickers (helical repeat (HR)1 and HR2), and a flexible 25-residue linker as the spacer. Inspired by disordered linkers in sticker-spacer systems, we designed a single linker using rational design principles as follows. First, the amino-acid composition of the unstructured linker was chosen on the basis of the propensities of residues in natural intrinsically disordered proteins^38^. Second, we gave the sequence an overall net-zero charge and a high hydrophilicity to achieve balanced and effective solvation^39^. Full details of linker design are given in Supplementary Fig. 1. Third, glycine residues were used as helical caps to prevent helical readthrough into the linker^40^. Returning to HR1 and HR2, to avoid interactions with intracellular nucleic acids and potential complex coacervate formation, the overall pI of the HERD was lowered from 9.4 to 4.7 by exchanging the original, solvent-facing lysine (Lys, K) residues for glutamate (Glu, E) in the HRs (Fig. 1d)^41,42^. As an initial client protein and to facilitate imaging, the monomeric fluorescent protein mEmerald^43^ was fused to the C terminus of the HERD. Finally, an N-terminal His tag followed by a TEV-cleavage site was added for purification (Fig. 1c). We named the final construct His–HERD-0–mGFP, or HERD-0–GFP for short. The constructs below are similar but with the HERD varied (Supplementary Tables 1 and 2).

**Fig. 2:**
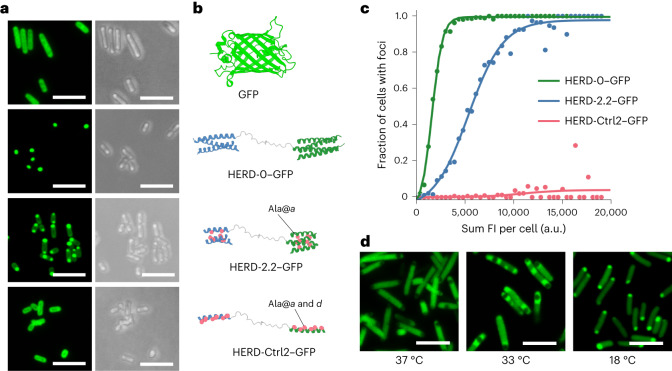
Weakening the designed helix–helix interactions leads to soluble protein that can condense in cells. **a**, Iteration of the HERD designs visualized by light microscopy. Fixed-cell confocal microscopy images of *E. coli* cells expressing (from top to bottom): soluble GFP (His-TEV–GFP; control), the initial HERD-0–GFP design, the final variant HERD-2.2–GFP, and a control construct with a monomeric helical region HERD-Ctrl2–GFP (control). GFP fluorescence images (488 nm) are shown left (green) and brightfield transmission images on the right (grey). Scale bars, 5 μm. **b**, Iteration of the HERD designs visualized by light microscopy. Cartoons of the helical regions of the HERD design, with Ala substitutions highlighted in pink. Linkers and GFP are mostly omitted for clarity. **c**, Automated image analysis of protein condensation in *E. coli* cells expressing HERD-0–GFP (green; *n* = 5,782), HERD-2.2–GFP (blue; *n* = 5,993), and HERD-Ctrl2–GFP (pink, *n* = 7,923). *E. coli* cells were binned according to their total intra-cellular fluorescence (FI, *x* axis) and the fraction of cells identified as displaying intracellular foci (*y* axis). *E. coli* cells were grown for 6 h after induction at 18 °C and collected hourly for imaging and automated foci detection. **d**, Live-cell confocal microscopy images of HERD-2.2–GFP in *E. coli* grown and imaged at the indicated temperature. At 37 °C and 33 °C the formation of non-fluorescent inclusion bodies is visible by non-fluorescent cellular foci (Supplementary Fig. 14). Scale bars, 5 μm.

Expression of HERD-0–GFP in *E. coli* resulted in fluorescent intracellular foci (Fig. 2a,b and Supplementary Fig. 2), whereas expression of mEmerald alone gave uniformly distributed fluorescence, indicating that protein condensation was specific to the HERD-0–GFP construct (Supplementary Fig. 3). However, western blotting showed that the majority of the de novo polypeptide was irreversibly aggregated in the insoluble fraction (Supplementary Fig. 4), which we attributed to the strong (≤ nM affinity) interactions between the HRs^26,37^. Therefore, to weaken these CC interactions and the net PPIs, initially, we shortened the HRs from the standard 28 residues to 21 residues (Fig. 1e). In terms of condensation and solubility, this gave polypeptides (HERD-1.1–GFP) that behaved similarly to the original HERD-0–GFP (Supplementary Figs. 5 and 6). Therefore, we applied a combination of the following strategies to modulate condensation: (1) further shortening the HRs; (2) mutating interfacial hydrophobic residues to alanine (Ala) (Fig. 1f); and (3) introducing overall helix-destabilizing mutations^44^ outside of the hydrophobic interface (Fig. 1g). This gave the redesigns HERD-2.1–GFP through 2.4. The resulting constructs improved solubility while retaining protein condensation in cells (Fig. 2a,b and Supplementary Figs. 7 and 8). However, destabilization of the HRs beyond the recognized limit of CC formation—for instance, by reducing their lengths to two heptads or less, and completely disrupting core packing (HERD-2.5–GFP through 2.8, and HERD-Ctrl1–GFP and 2) (ref. ^45^)—resulted in the loss of protein condensation and gave largely dispersed and soluble constructs (Supplementary Figs. 9–12). At this stage, different linkers were tested to explore the effect of linker length and polarity on protein condensation (HERD-3.1–GFP through 3.4; Supplementary Tables 1 and 2 and Supplementary Fig. 13). However, in our system at least, these linker variations had no discernible impact on protein condensation (Supplementary Fig. 14).

Automated image detection of foci in *E. coli* was used to quantify changes in cellular protein concentration and condensation (Fig. 2c and Supplementary Fig. 15). For HERD-0–GFP, this revealed that condensates formed at low protein concentrations, suggesting aggregation. By contrast, HERD-2.2–GFP only formed condensates when a critical intracellular concentration was reached, indicative of threshold phase separation. Interestingly, this behaviour was also temperature dependent: in *E. coli* grown at 37 °C there was no observable condensation, while at lower temperatures (33 °C and 18 °C) enriched protein condensates were observed (Fig. 2d and Supplementary Fig. 16). Live imaging of cells grown at 37 °C as the temperature was reduced confirmed the temperature-dependent appearance of condensates (Supplementary Fig. 16). Therefore, HERD-2.2–GFP was selected for further analysis. This has 2.5-heptad (17-residue) HRs with Ala residues at the ***a*** positions, isoleucine (Ile) at the ***d*** sites, and the originally designed linker.

### Purified HERD-2.2–GFP phase separates in vitro

**Fig. 3:**
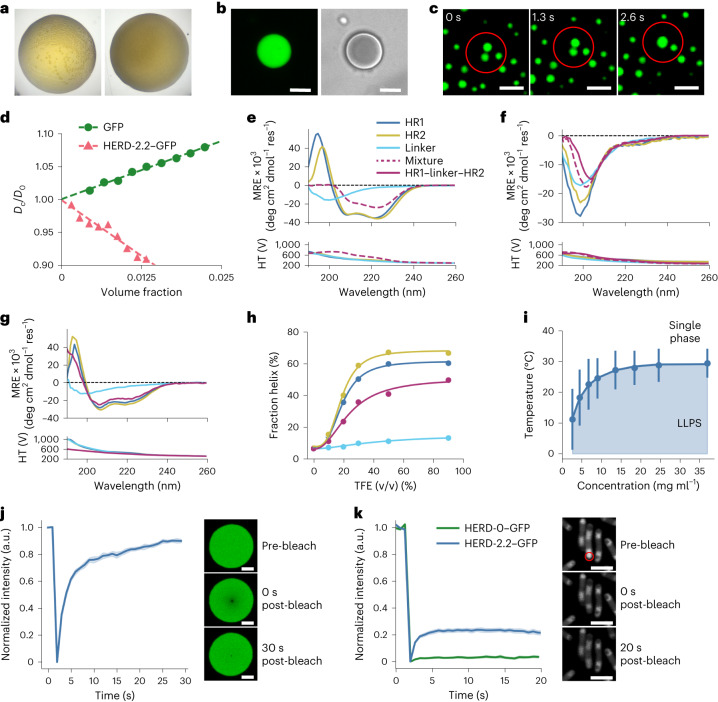
HERD-2.2–GFP forms de-mixed liquid droplets in vitro and in cells. **a**, Images of HERD-2.2–GFP showing macroscopic liquid de-mixing (left) and amorphous aggregation (right) in 0.8 μl droplets, approximately 1–2 mm in diameter. **b**,**c**, Confocal microscopy of 1 mM HERD-2.2–GFP de-mixed droplets in 4% PEG 3350 (**b**), and showing the coalescence of 2 such droplets circled in red. Coalescence occurred over a 2–3 s timescale, imaging every 648 ms (**c**). Scale bar, 5 μm. **d**, The dependence of *D*_C_/*D*_0_ (*D*_C_, collective diffusion coefficient; *D*_0_, free-particle diffusion coefficient) on protein volume fraction for GFP (green circles) and HERD-2.2–GFP (pink triangles) measured by DLS at 20 °C. **e**–**g**, CD data for the chemically synthesized HR and linker peptides: CD spectra for the HERD-0 (**e**) and HERD-2.2 (**f**) peptides at 500 μM (per peptide); CD spectra of HERD-2.2 peptides at 100 µM in 50% TFE, 5 °C (**g**). HR1, blue; HR2, yellow; linker, teal; mixture, purple dashes; HR1–linker–HR2, purple solid. HT is the applied high tension voltage (V). **h**, Fraction helix of HERD-2.2 peptides through a TFE titration, at 100 µM peptide, 5 °C. **i**, Phase diagram of HERD-2.2–GFP from turbidity measurements in 4% PEG 3350. Bars indicate the difference between *T*_cloud_ and *T*_clear_ from individual biophysical measurements where all attempts at repetition were successful. **j**, FRAP of HERD-2.2–GFP droplets in vitro. *t*_1/2_ = 1.54 ± 0.21 s. Data are represented as mean ± standard error from *n* = 13 biologically independent experiments. Representative images of pre-bleach, post-bleach frame 1 and the final post-bleach frame shown alongside. Scale bar, 5 μm. **k**, FRAP of HERD-2.2–GFP (blue) and HERD-0–GFP (green) condensates in cells. The red circle indicates the bleached area. *t*_1/2_ for HERD-2.2–GFP is 0.46 ± 0.11 s. Data are represented as mean ± standard error from *n* = 13 (HERD-2.2–GFP) or *n* = 19 (HERD-0–GFP) biologically independent cells. Representative images of pre-bleach, post-bleach frame 1 and the final post-bleach frame shown alongside. Scale bar, 5 μm. Common conditions for all experiments: 125 mM NaCl, 20 mM Tris–HCl, pH 7.5.

The intact HERD-2.2–GFP protein was purified for in vitro studies (Supplementary Fig. 17). Initially, different buffers, ionic strengths and molecular crowders^46^ (that is, PEG 3350) were screened in 0.8 μl droplets by automated imaging to identify conditions for phase separation (Supplementary Fig. 18). We observed both general protein aggregation and potential liquid–liquid de-mixing, characterized by the formation of spherical macroscopic droplets (Fig. 3a). The optimal conditions for droplet formation were 4% PEG 3350 and 125 mM NaCl in Tris–HCl buffer (pH 7.5). Observations under these conditions by confocal microscopy revealed that the droplets were spherical and coalesced, indicative of liquid-like behaviour (Fig. 3b,c and Supplementary Figs. 19 and 20). Variable-temperature measurements showed that droplet formation occurred as the temperature was reduced from 40 °C to 5 °C (Supplementary Video 1), and was reversible upon reheating (Supplementary Video 2). All of these properties are consistent with the formation of liquid condensates formed by LLPS. Also, we tested for any contribution of the N-terminal His-TEV tag: following TEV cleavage, the shortened HERD-2.2–GFP still underwent phase separation similar to the full-length protein, though it required more molecular crowding agent, 10% PEG 3350 (Supplementary Figs. 21 and 22).

To examine the self-interactions of the designed polypeptide further, we determined the net interaction parameter (diffusivity), *k*_D_, for GFP and HERD-2.2–GFP (Fig. 3d)^47^. Positive values of *k*_D_ indicate a protein with repulsive net interactions, while negative values indicate attractive PPIs. While GFP was slightly repulsive by itself (*k*_D_ = 3.5 ± 0.2), the fusion protein had net attractive PPIs (*k*_D_ = −6.9 ± 0.6) consistent with its behaviour in cells and in vitro. Moreover, this *k*_D_ value is similar to those measured for other proteins that undergo LLPS^48^. Therefore, the designed polypeptide tag introduces attractive interactions to the slightly repulsive GFP molecule, making the net overall PPIs of the fusion construct attractive.

### Nascent helicity helps drive condensation of HERD-2.2–GFP

To probe changes in secondary structure content of HERD-2.2–GFP, we followed de-mixing by circular dichroism (CD) spectroscopy. CD spectra were dominated by the β structure of GFP, and showed no detectable changes when recorded at different protein concentrations and temperatures (Supplementary Fig. 23). To investigate this further, we made the variants of the HR1 and HR2 sequences from our design trajectory and the linker sequence by solid-phase peptide synthesis (SPSS) (Supplementary Table 3 and Supplementary Figs. 24 and 25). As expected, the CD spectra of the original HRs from the HERD-0 domain were highly α helical, with intense minima at 208 nm and 222 nm (Fig. 3e and Supplementary Fig. 26); while the spectrum of the linker had a single minimum at 200 nm and low signal at 222 nm, indicating disorder. By contrast, for the HR variants of HERD-2.2—that is, with Ala at ***a*** positions, and truncated to 2.5 heptads—this helicity was lost, although some residual structure over the disordered linker peptide may be present (Fig. 3f and Supplementary Fig. 26). Mixing this HR1, HR2, plus linker combination did not induce structure (Fig. 3f). Moreover, a chemically synthesized HR1–linker–HR2 peptide for HERD-2.2 appeared largely unstructured, even in the presence of PEG (Supplementary Figs. 26 and 27).

These in vitro CD data were unexpected given our design hypothesis that α-helical domains drive PPIs and phase separation. However, it is possible that destabilized HRs still form nascent helices that associate transiently in the crowded environment of phase-separated droplets in cells and in vitro^49^. To investigate the potential for nascent helicity in the HERD-2.2 design, we recorded CD spectra in the presence of trifluoroethanol (TFE)^50^. In TFE titrations, the HR1 and HR2 peptides shifted to α-helical conformations, whereas the linker remained unstructured (Fig. 3g,h and Supplementary Fig. 28). Furthermore, the HR1–linker–HR2 peptide also shifted to a partially α-helical conformation. These experiments indicate that, while largely unstructured in aqueous solution, the HRs of HERD-2.2 retain propensity to form α helices.

Encouraged by these data, we tested for nascent helicity and helix–helix interactions in cells by mutating HR1 and HR2 in the successful HERD-2.2–GFP background to knock out any such structure and interactions (controls 1–7, Supplementary Table 1). This was done in three different ways. For instance, we replaced the remaining large hydrophobic Ile residues with Ala in a 3-heptad HERD background, to resemble a known monomeric α helix (HERD-Ctrl1–GFP and Ctrl2, Fig. 2a,b)^51^. Also, to eliminate the amphipathicity of the HRs, we scrambled their sequences (HERD-Ctrl3–GFP). And, we introduced helix-breaking mutations into the HRs, for example, proline (Pro, P) or glycine (Gly, G) at various positions of the heptad repeats (HERD-Ctrl4–GFP through Ctrl7). All three redesigns showed significantly reduced or near-complete abolishment of protein condensation in cells (Fig. 2c and Supplementary Figs. 15 and 29). From these experiments, we posit that interactions between partially or transiently helical regions in the parent construct, HERD-2.2–GFP, contribute to condensation.

### HERD-2.2–GFP undergoes LLPS in vitro and in cells

Next, we mapped the binodal phase boundary of expressed and purified HERD-2.2–GFP by measuring the cloud-point as a function of temperature and protein concentration (Supplementary Fig. 30). These experiments started with a single phase at higher temperature and measured changes in turbidity as the phases separated upon cooling. This returned an upper critical solution temperature for an enthalpically driven phase transition (Fig. 3i). The process was reversible on heating with hysteresis between the solution cloud-point temperature (*T*_cloud_) and the clearing temperature (*T*_clear_) characteristic of protein LLPS. Further, the turbidity change accelerated with increased protein concentration consistent with faster nucleation.

To confirm the liquid nature of the condensates, we probed molecular diffusion within the droplets by fluorescence recovery after photobleaching (FRAP). First, droplets of purified HERD-2.2–GFP in vitro showed FRAP with a *t*_1/2_ of 1.54 s and near-complete recovery of signal, indicating highly mobile molecules (Fig. 3j). Next, we performed FRAP on the HERD-2.2–GFP condensates directly in *E. coli* cells. Here we observed recovery of fluorescence with a similar rate to that measured in vitro (<1 s; Fig. 3k). However, compared with the bulk in vitro experiments, the amplitude of the final signal was considerably reduced (Fig. 3j,k). We attribute this to the confined system of the cell and, thus, the finite amount of fluorescent protein available to diffuse into the bleached region, which is large relative to the volume of the cell. This contrasts with the in vitro experiments where there is a very large excess of unbleached material to diffuse back into the bleached area. Nonetheless, in cells, asymmetrically bleached droplets nearly entirely re-equilibrated their fluorescence within 20 s after bleaching (Supplementary Fig. 31). Similar experiments with HERD-0–GFP aggregates in cells showed no fluorescence recovery, and asymmetrically bleached droplets did not re-equilibrate their fluorescence between the bleached and non-bleached areas (Fig. 3k and Supplementary Fig. 31). Thus, our design process progressed from insoluble CC-based constructs (with HERD-0) to biomolecular condensates with dynamic, liquid properties (with HERD-2.2) both in vitro and in cells.

### HERD-2.2 condensates can be functionalized in cells

**Fig. 4:**
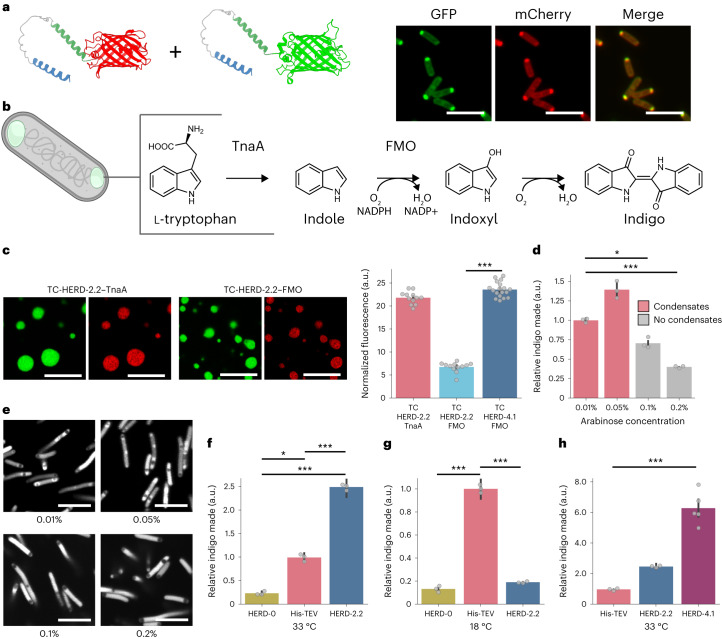
HERD-2.2-tagged enzymes form functional MLOs. **a**, Confocal microscopy images of HERD-2.2–GFP and HERD-2.2–mCherry co-expressed in *E. coli*. GFP fluorescence at 488 nm (green), mCherry fluorescence at 561 nm (red), and the merged channels. Scale bar, 5 μm. **b**, Schematic for the in cell co-localization of TnaA and FMO using the HERD-2.2 polypeptide and the subsequent enzymatic production of indigo dye. **c**, Left: confocal microscopy images of de-mixed droplets in vitro formed by mixing HERD-2.2–GFP plus TC-HERD-2.2–TnaA (top), and HERD-2.2–GFP plus TC-HERD-2.2–FMO (bottom), with fluorescent reporters GFP (green) and TC-ReAsH at 561 nm (red). Scale bar, 5 μm. Conditions: 125 mM NaCl, 4% PEG 3350, 20 mM Tris pH 7.5, 500 μM HERD-2.2–GFP, 25 μM HERD-2.2–TnaA or HERD-2.2–FMO. Right: quantification of the fluorescence intensity (561 nm for TC-ReAsH) in de-mixed droplets formed by HERD-2.2–GFP. Fluorescence intensity was normalized by subtracting the fluorescence intensity within HERD-2.2–GFP droplets containing ReAsH dye but no TC-tagged proteins. Data are represented as mean ± the standard error from *n* = 12 (TC-HERD-2.2–TnaA, TC-HERD-2.2–FMO) or *n* = 18 (TC-HERD-4.1–FMO) independent biological measurements. *P* = 0.001 (***) by one-way analysis of variance (ANOVA) and Tukey’s honestly significant difference (HSD) post-hoc test. **d**, Relative indigo produced by cells grown at 33 °C co-expressing HERD-2.2–GFP, HERD-2.2–FMO and HERD-2.2–TnaA at increasing concentrations of the FMO- and TnaA-containing fusions. The latter were under control of the pBAD promotor with concentrations varied by increasing the concentration of d-arabinose (0.01% to 0.2% w/v). Indigo production (*A*_610_) was normalized both to cell density (OD_700_) and the relative expression levels of the rate-limiting enzyme (FMO) measured by western blotting. Data are represented as mean ± standard error from *n* = 3 biologically independent experiments for each sample. *P* = 0.0318 (*) and *P* = 0.001 (***) by one-way ANOVA and Tukey’s HSD post-hoc test. **e**, Confocal microscopy images of HERD-2.2–GFP condensates in cells at increasing HERD-2.2–FMO and HERD-2.2–TnaA concentrations (0.01% to 0.2% w/v d-arabinose). Cells grown and imaged at 33 °C. Scale bar, 5 μm. **f**–**h**, Relative indigo produced by cells co-expressing different GFP-, TnaA- and FMO-containing fusions and grown at 33 °C or 18 °C as stated. Indigo production (*A*_610_) was normalized both to cell density (OD_700_) and to the relative expression levels of FMO (AU) measured by western blotting (Supplementary Figs. 37, 38, 39 and 41). Data are represented as mean ± standard error from *n* = 3 (His-TEV, HERD-0 and HERD-2.2) or *n* = 5 (HERD-4.1) biologically independent samples. Statistical testing was performed by one-way ANOVA and Tukey’s HSD post-hoc test. **f**, HERD-0 versus His-TEV *P* = 0.0285 (*); His-TEV versus HERD-2.2 *P* = 0.001 (***); HERD-0 versus HERD-2.2 *P* = 0.001 (***). **g**, HERD-0 versus His-TEV *P* = 0.001 (***); His-TEV versus HERD-2.2 *P* = 0.001 (***). **h**, His-TEV versus HERD-4.1 *P* = 0.001 (***).

Finally, we tested the HERD-2.2 polypeptide as a component for designing functional MLOs with alternate client proteins. Initially, we swapped mEmerald for mCherry to give two fluorescent constructs HERD-2.2–GFP and HERD-2.2–mCherry (Fig. 4a). When co-expressed in *E. coli*, these co-localized to the same condensates (Fig. 4a and Supplementary Fig. 32). Next, we replaced the fluorescent proteins with the enzymes tryptophanase (TnaA) and flavin-containing monooxygenase (FMO) to give HERD-2.2–TnaA and HERD-2.2–FMO (Supplementary Fig. 33). The tetrameric TnaA and dimeric FMO enzymes together catalyse the two-step conversion of tryptophan to indigo (Fig. 4b), which we sought to test in the HERD-based system^52^. Purified, His-tagged HERD-2.2–TnaA formed de-mixed liquid droplets similar to HERD-2.2–GFP, while HERD-2.2–FMO did not undergo phase separation in vitro (Supplementary Fig. 34). We attribute this to TnaA and GFP having very similar calculated net charges (both −6 at pH 7.5), whereas FMO is highly negatively charged (net charge −21 at pH 7.5). Again, this indicates that it is the net PPIs made by whole construct and not just by the de novo polypeptide that lead to condensation.

As HERD-2.2–FMO did not undergo LLPS in isolation, we tested if HERD-2.2–GFP could facilitate the co-condensation of the FMO and TnaA constructs. First, we confirmed that HERD-2.2–GFP droplets in vitro could recruit both HERD-2.2–FMO and HERD-2.2–TnaA separately by adding tetra-cysteine (TC) motifs into the flexible linkers (giving TC–HERD-2.2–FMO and TC–HERD-2.2–TnaA, Supplementary Table 1) and subsequently labelling with TC-ReAsH II (Fig. 4c and Supplementary Figs. 35 and 36)^53^. Moreover, we confirmed co-localization of the two enzymes into droplets in cells both individually and together using HERD-2.2–GFP and HERD-2.2–mCherry fusions as follows (Supplementary Fig. 37). Individually, both HERD-2.2–mCherry-TnaA and HERD-2.2–mCherry-FMO localized to HERD-2.2–GFP condensates in *E. coli*; and when co-expressed, HERD-2.2–GFP-TnaA and HERD-2.2–mCherry-FMO co-localized, confirming dual enrichment in HERD-2.2 condensates in cells.

Next, having confirmed that both tagged proteins co-condense with HERD-2.2 droplets, we tested the effect of enzyme co-localization directly in cells. For this, HERD-2.2–TnaA and HERD-2.2–FMO fusions were co-expressed under the arabinose promotor on a low-copy-number vector to control their expression, while HERD-2.2–GFP was expressed from the T7 promotor to generate the de-mixed compartments. First, cells expressing the three constructs were grown at 33 °C, close to where the liquid-like HERD-2.2–GFP condensates were identified. We observed that high expression levels of the tagged enzymes reduced the number of condensates formed, suggesting the maximum enzyme-loading capacity of the condensates had been reached. Nonetheless, and interestingly, cells with condensates produced more indigo than those without condensates and higher expression levels of the enzymes (Fig. 4d,e and Supplementary Figs. 38 and 39). Cells with HERD-2.2 condensates produced approximately 2.3 times more indigo than free enzymes expressed at similar levels, suggesting that the efficiency of the two-enzyme cascade was improved by condensation into a liquid state (Fig. 4f and Supplementary Fig. 40). However, when the condensates were formed in cells grown at 18 °C there was a marked reduction in indigo compared with the free enzymes (Fig. 4g and Supplementary Fig. 41). Consistent with this, FRAP of HERD-2.2–GFP performed in cells grown at 18 °C revealed the condensates to be less liquid-like than those grown at 33 °C, and possibly amorphous aggregates or gels (Supplementary Fig. 42). Furthermore, under both growth temperatures, condensates seeded by HERD-0–GFP showed significantly reduced indigo production compared with the free enzymes (Fig. 4f,g and Supplementary Figs. 40 and 41). This suggests that the HERD-0-based design simply sequesters the enzymes and makes them inaccessible to substrate.

To improve the effect of condensation on indigo production, we sought to tailor our HERD design more specifically to the client enzymes. As noted above, HERD-2.2–FMO enriched less strongly than HERD-2.2–TnaA in HERD-2.2–GFP droplets (Fig. 4c). We hypothesized that this was due to FMO being highly negatively charged, and that the poor co-condensation of HERD-2.2–FMO limits any potential improvement in productivity from phase separation. Therefore, to improve FMO loading of the condensates, we modified HERD-2.2–FMO to reduce its overall net negative charge to give HERD-4.1–FMO (Supplementary Table 1). Encouragingly, cells expressing HERD-4.1–FMO, HERD-2.2–TnaA and HERD-2.2–GFP produced more indigo than the HERD-2.2–FMO design, and >2.5 times more than the free enzymes (Supplementary Fig. 43). Moreover, when normalized to the concentration of FMO, the rate-limiting enzyme in the cascade, this equated to a six-fold increase in productivity over the free enzymes (Fig. 4h). To confirm that co-condensation had been improved by the redesign, we assessed the co-condensation of HERD-4.1–FMO using TC-ReAsH II in vitro (Supplementary Figs. 35 and 36). The redesigned protein was 3.5-fold more enriched in HERD-2.2–GFP droplets than the original HERD-2.2–FMO design (Fig. 4c). These experiments show that the HERD tag can be redesigned rationally to improve condensation of client proteins and enhance pathway efficiency.

## Conclusion

In summary, we have designed polypeptide tags that can be fused to client proteins enabling the resulting fusions to undergo phase separation in vitro and in living cells. Rather than designing geometrically defined and rigid proteins, we focused on creating constructs that are reminiscent of IDRs to promote long-range disorder and create macroscopic condensates with desired physical properties. The polypeptides are designed from first principles by concatenating two designed helical oligomerization domains via an artificial intrinsically disordered linker to give ~100-residue sequence. When appended as an N-terminal tag to a green fluorescent protein, this produces insoluble fluorescent aggregates in cells. However, the assemblies can be rendered soluble by destabilizing the helical regions and weakening their interactions. Notably, only a handful of designs had to be screened to identify sequences with the desired characteristics, and in the final design, condensate formation depends on protein concentration and temperature indicative of reversible liquid–liquid phase separation (LLPS). Through a series of in vitro and in-cell experiments using soft-matter physics and biophysical methods, we show that the protein condensates are highly dynamic and behave like de-mixed liquid droplets. Finally, we demonstrate that the client fluorescent protein can be substituted by enzymes to assemble functional fusion proteins that co-condense into liquid states. In cells, these dual-enzyme organelles give increased activities over the freely expressed, soluble enzymes. Further, we show that the designed tag can be modified to suit the client protein of interest to improve co-condensation and activity. Interestingly, the six-fold enhancement in productivity that we find matches the theoretical enhancement from co-localizing two enzymes^54^, suggesting that we have achieved highly efficient co-condensation.

Others have reported the successful engineering of natural condensing proteins in cells to explore the potential of MLOs in synthetic biology^13,15,17,18,55,56^. Recent examples have demonstrated the capability for condensates to augment cells with artificial functions approaching the complexity of natural organelles^14,57^, and to generate compartments that can be modulated through rational changes to their scaffold proteins^16^. Engineered condensates have also incorporated designed enzymatic reactions, creating functional MLOs^58^. In addition to co-localizing client proteins, our system has a potentially useful thermo-switchable behaviour that allows induction and dissolution of protein condensates in vitro and in cells by modifying the conditions or the cell-growth temperature. Furthermore, the material properties of these condensates can be varied with temperature, switching from gel-like condensates to liquid-like droplets. This directly affects the efficiency of the co-condensed enzyme cascade, as measured in our system by the production of indigo. This temperature-dependent switching of phase behaviour potentially permits the control of protein condensation and function using a simple control mechanism.

Overall, we anticipate that our de novo designed polypeptide tag will provide a valuable tool for studying biomolecular condensation, and for developing MLOs in synthetic or natural biological systems both in vitro and within cells. Furthermore, the relative simplicity of our designs and the ease with which they can be redesigned to access soluble, dynamic condensed and aggregated states should allow them to be adapted for other experiments and applications.

## Methods

### Materials

All chemicals and biological materials were obtained from commercial suppliers. *E. coli* BL21(DE3), Q5 DNA polymerase, T4 DNA ligase and restriction enzymes were purchased from New England BioLabs. Genes were ordered as g-blocks from IDT, and primers were ordered from Eurofins Genomics. Anti-His primary antibody (H1029, clone HIS-1) was purchased from Sigma-Aldrich, and goat anti-mouse IgG (H + L) secondary antibody HRP (31430) was purchased from Invitrogen. Lysogeny broth (LB, Lennox) was purchased from Sigma-Aldrich.

### Statistics and reproducibility

All experiments were repeated at least in triplicate to ensure reproducibility, and representative data shown, and statistical tests were performed on a minimum of three independent replicates.

### Calculation of protein pI and net charge

Protein physical and chemical parameters were calculated from the primary amino acid sequence using the ExPASy ProtParam tool^59^.

### Protein expression

The genes encoding the HERDs and enzymes (FMO, TnaA) were codon optimized for expression in *E. coli* and ordered from IDT as g-blocks. Constructs were cloned into pET38a(+) derivative vectors (pDICa (ampicillin selection marker) or pDICc (chloramphenicol selection marker); Supplementary Fig. 44) kindly donated by M. Lee, using XbaI and NdeI restriction sites and T4 DNA ligase.

Plasmids (25 ng) were transformed into *E. coli* BL21(DE3) competent cells by heat shock and plated on LB agar plates supplemented with appropriate antibiotics (100 μg ml^−1^ ampicillin, 25 μg ml^−1^ chloramphenicol). Following overnight incubation at 37 °C, a single colony was used to inoculate 5 ml LB and grown overnight (37 °C, 200 rpm). Fresh LB was inoculated 1:100 from the overnight culture and grown to OD_600_ of 0.4–0.6 (37 °C, 200 rpm). Protein expression was then induced using 400 μM isopropyl β-d-1-thiogalactopyranoside (vectors with the T7 promotor) or varying d-arabinose concentrations (vectors with the arabinose promotor).

### In-cell confocal microscopy

For confocal microscopy, 50 ml of LB was inoculated from the overnight culture. After induction of protein expression, cultures were grown at 18 °C, shaking at 200 rpm typically for 5 h. One millilitre of culture was collected, and cells were pelleted by centrifugation (3,000*g*, 3 min). For fixed cells, pellets were washed three times in phosphate-buffered saline (PBS), before fixing by incubating in 1 ml of 2% paraformaldehyde in PBS for 15 min at room temperature. Pellets were washed a further three times in PBS before resuspending in 50 μl PBS. Fixed cells were mounted in ProLong Diamond Antifade Mountant (Invitrogen). For live cell imaging, cells were grown as described above, with variable growth temperatures (18–37 °C) after induction. One millilitre of culture was collected by centrifugation and immediately resuspended in 50 μl PBS. Fifteen microlitres of cell suspension was sealed onto a glass slide under a coverslip with nail polish to prevent evaporation and imaged immediately. Confocal images were collected using a Leica SP5II microscope using a 63× objective lens, running Leica LAS X (3.7.6). For temperature-controlled live-cell microscopy, a variable temperature microscope stage was used (Linkam), set to the indicated temperature 30 min before imaging to ensure equilibration. All slides, coverslips and materials were pre-heated to the indicated temperature before use, and cells were transported on a temperature-controlled platform to the imaging stage. Fixed cell images are represented as maximum intensity projections, assembled in ImageJ.

### Western blotting

For western blotting, 50 ml of LB was inoculated from the overnight culture and grown at 18 °C, 200 rpm. Pellets were collected by centrifugation (3,000*g*, 10 min) after normalization to cell density (OD_600_). Pellets were lysed by resuspension in BugBuster lysis buffer (Millipore) with benzonase nuclease (Millipore) and incubated at 37 °C for 30 min. Suspensions were then snap frozen three times in liquid nitrogen to ensure complete cell lysis. For separation of cellular soluble and insoluble fractions, suspensions were centrifuged (18,000*g*, 20 min). The supernatant (soluble fraction) was removed, and the pellet (insoluble fraction) was resuspended in an equal volume of BugBuster. For sodium dodecyl sulfate–polyacrylamide gel electrophoresis (SDS–PAGE), samples were mixed with appropriate volumes of reducing SDS loading dye and heated to 95 °C for 5–10 min. Six microlitres of sample was loaded alongside 6 μl of colour pre-stained protein standard, broad range (NEB) onto 12% acrylamide/bis-acrylamide (29:1) gels and run at 180 V for 1 h, or until the loading dye reached the bottom of the gel. For western blotting, proteins were transferred onto a 0.2 μm polyvinylidene fluoride membrane (Cytiva) using Power Blotter 1-Step Transfer Buffer (Invitrogen) for 10 min at 1.3 A. Membranes were blocked in 4% skimmed milk powder with 0.1% Tween-20 in PBS for 30 min with gentle rocking. Membranes were then incubated with anti-His primary antibody 1:5,000 in 4% milk in PBS-T (Sigma) for 2 h. Membranes were washed three times for 5 min in PBS-T, before adding the HRP-conjugated secondary antibody 1:10,000 in 4% milk in PBS-T (Invitrogen) for 1 h. Membranes were washed a further three times for 5 min in PBS-T, before adding 2 ml of SuperSignal West Pico Plus chemiluminescent substrate (Thermo), and incubating for 1 min before imaging using a G:Box Chemi-XT4 chemiluminescent imager (SynGene) for the desired interval.

### Automated image analysis

Images for automated image analysis were collected from *E. coli* cells expressing HERD variants. Cells for imaging were collected beginning in the log phase of growth at OD_600_ of 0.4–0.6. This was established as timepoint 0. Cells were collected every hour after timepoint 0 for 6 h, with samples fixed for later imaging as described. After 6 h, the cultures reached approximately OD_600_ of 2–2.5. Brightfield and fluorescent microscopy images of *E. coli* were quantified using the ModularImageAnalysis (MIA; v0.21.11) plugin for Fiji^60–63^. Before detection of *E. coli*, brightfield images stacks were normalized using sliding paraboloid background subtraction (radius 10 px). From these, single slices chosen for optimal feature contrast were extracted using a modified version of the Stack Focuser ImageJ plugin^64^. The focused images were then intensity normalized and subject to further background correction by pixelwise division with 2D Gaussian-filtered (sigma 10 px) variants of the same images. The corrected brightfield images were down-sampled 2× in *XY* before being passed to the StarDist Fiji plugin for detection of *E. coli*^65–67^, using a model trained on the DeepBacs *E. coli* dataset^68^. To account for overlap between adjacent cells detected via StarDist, binary images showing detected cells were created and re-segmented using the distance-based watershed transform. Final *E. coli* detections were obtained from the segmented images using connected components labelling. Foci were detected in maximum intensity *z*-axis projections of fluorescent image stacks. These images were passed through a 2D top-hat filter (radius 5 px) to remove general cell background intensity. The images were then converted to binary maps using a fixed global intensity threshold and adjacent foci separated using another distance-based watershed transform. Markers for the watershed transform were acquired using TrackMate’s LoG spot detector^69^. This detector convolves the image with a Laplacian of Gaussian kernel to enhance spot-like features of a specific size (radius 4 px) and detects foci as features in the convolved image brighter than a set threshold. Foci were detected from the segmented images using connected components labelling^70^. Number, area and fluorescent intensity statistics for each measured cell and focus were measured and exported as a single Excel spreadsheet for downstream analysis.

### Protein purification

For protein purification, 1–12 litres of LB was inoculated 1:100 from an overnight culture and grown at 18 °C, shaking at 200 rpm. Cell pellets were resuspended in buffer containing 500 mM NaCl, 20 mM Tris–HCl pH 7.5, 2 M urea, 50 mM imidazole and one tablet cOmplete protease inhibitor (Roche), and lysed by sonication on ice (3 s on, 1 s off, 70% amplitude, 15 min). The lysate was centrifuged (18,000*g*, 20 min) and the supernatant filtered through a 0.2 μm filter to clarify. Protein purification was performed using an Äkta Pure (Cytiva) at 4 °C, with chromatograms monitored at 280 nm. The clarified lysate was applied to a HisTrap HP (Cytiva) immobilized metal affinity chromatography column, pre-equilibrated in 500 mM NaCl, 20 mM Tris–HCl pH 7.5, 2 M urea and 50 mM imidazole. The column was washed until *A*_280_ was re-stabilized (typically 3–4× the column volume), before eluting the bound protein with a gradient of imidazole (50–500 mM). Recombinant protein was further purified by size exclusion chromatography using a HiLoad 16/600 Superdex 200 pg exclusion column (Cytiva) with a flow rate of 1 ml min^−1^. Size exclusion was performed using a 20 mM Tris–HCl pH 7.5, 2 M urea running buffer and elution monitored by *A*_280_. Protein fractions were identified by SDS–PAGE and the relevant fractions pooled. Protein samples were finally desalted using a HiPrep 26/10 desalting column (Cytiva) into 20 mM Tris–HCl pH 7.5, aliquoted, flash frozen and stored at −70 °C.

### CD spectroscopy

CD data were collected on a JASCO J-810 or J-815 spectropolarimeter fitted with a Peltier temperature controller (Jasco UK) running Spectra Manager (1.55). Full spectra were measured between 190 nm and 260 nm with a 1 nm step size, 100 nm min^−1^ scanning speed, 1 nm bandwidth and 1 s response time. Spectra were measured at 5 °C unless otherwise stated. Baselines recorded using the same buffer, cuvette and parameters were subtracted from each dataset. For experiments in TFE, the protein in buffer was mixed with neat TFE to produce the stated concentrations. The spectra were converted from ellipticities (deg) to mean residue ellipticities (MRE, (deg cm^2^ dmol^−1^ res^−1^)) by normalizing for concentration of peptide bonds and the cell path length using the following equation:

$${{\mathrm{MRE}}}\,\left(\deg {{{\mathrm{cm}}}}^{2}\;{{{\mathrm{dmol}}}}^{-1}\;{{{\mathrm{res}}}}^{-1}\right)=\,\frac{\theta \times 100}{c\times l\times b}\,$$

where the variable *θ* is the measured difference in absorbed circularly polarized light in millidegrees, *c* is the millimolar concentration of the specimen, *l* is the path length of the cuvette in cm and *b* is the number of amide bonds in the polypeptide, for which the N-terminal acetyl bond was included but not the C-terminal amide. Peptide concentration was determined at 280 nm (*ε*_280_(Trp) = 5,690 cm^−1^, *ε*_280_(Tyr) = 1,280 cm^−1^) (ref. ^71^) (for peptides 1–9) or by measuring the peptide bond at 214 nm (ref. ^72^) (for peptide 10) using a Nanodrop 2000 (Thermo) spectrometer. Fraction helix (%) was calculated from MRE at 222 nm using the following equation:

$${{\mathrm{Fraction}}}\,{{\mathrm{helix}}}\,\left( \% \right)=100\times \frac{{{{\mathrm{MRE}}}}_{222}-{{{\mathrm{MRE}}}}_{{{\mathrm{coil}}}}}{-42500\times \left(1-3/n\right)-{{{\mathrm{MRE}}}}_{{{\mathrm{coil}}}}}$$

where MRE_coil_ is calculated by 640-45*T; *T* is the temperature in °C; and *n* is the number of amide bonds in the sample (including the C-terminal amide)^73^.

### Peptide synthesis

Solid-phase peptide synthesis (SPPS) reagents were purchased from Cambridge Reagents with the exception of *N*,*N*′-diisopropylcarbodiimide (DIC) purchased from Carbosynth. Rink amide MBHA resin and Fmoc-protected amino were purchased from Merck. SPPS was performed on a Liberty Blue automated peptide synthesizer (CEM) with inline ultraviolet (UV) monitoring. All peptides were synthesized as the C-terminal amide on Rink amide MBHA resin, with DIC/Oxyma as the coupling reagents. Fmoc was removed using 20% v/v morpholine:dimethylformamide. All peptides were N-terminally acetylated through treatment with pyridine (0.5 ml) and acetic anhydride (0.3 ml) in dimethylformamide (9.2 ml) and shaking at room temperature for 20–60 min. Peptides were cleaved from the resin with addition of 95:2.5:2.5 v/v trifluoroacetic acid (TFA):H_2_O:triisopropylsilane and shaking at room temperature for 3 h. Following collection of the cleavage solution, TFA was evaporated under a N_2_ stream followed by precipitation with ice cold diethyl ether. Precipitates were collected by centrifugation and dissolved in 50:50 v/v acetonitrile (MeCN):H_2_O. Crude peptides were lyophilized to yield a white or off-white powder.

### Peptide purification

Peptides were purified by reverse-phase high-performance liquid chromatography (HPLC) on a Phenomenex Luna C18 stationary phase column (150 × 10 mm, 5 μm particle size, 100 Å pore size) using a preparative JASCO HPLC system. Crude peptide was dissolved at 3–5 mg ml^−1^ in 0–20% v/v acetonitrile with 0.1% TFA. A (0–20)–100% gradient of acetonitrile with 0.1% TFA over 30–45 min was used to separate the target peptide. Chromatograms were monitored at wavelengths of 220 and 280 nm. The identities of the peptides were confirmed using mass spectrometry. Peptide purities were determined using a JASCO analytical HPLC system, fitted with a reverse-phase Kinetex C18 analytical column (100 × 4.6 mm, 5 μm particle size, 100 Å pore size). Fractions containing pure peptide were pooled and lyophilized.

### Mass spectrometry

Matrix-assisted laser desorption/ionization–time of flight (MALDI–TOF) mass spectra were collected on a Bruker UltraFlex MALDI–TOF mass spectrometer operating in positive-ion reflector mode. Peptides were spotted on a ground steel target plate using α-cyano-4-hydroxycinnamic acid dissolved in 1:1 acetonitrile:H_2_O as the matrix. Masses quoted are for the monoisotopic mass as the singly protonated species. Full electrospray ionization mass spectrometry spectra were acquired on a Synapt G2S (Waters) mass spectrometer equipped with an IMS-Q-TOF analyser and using an Advion Nanomate for robot chip-based nanospray ionization in positive mode. Five microlitres of a 50 μM peptide solution in 1:1 acetonitrile:H_2_O were generally injected for the analysis. Masses quoted are for the deconvoluted monoisotopic mass.

### TEV cleavage

Cleavage by TEV protease was performed using ProTEV Plus (Promega) with 1 mM dithiothreitol, 0.5 mM ethylenediaminetetraacetic acid, 18 mg of HERD-2.2–GFP, and 200 units of ProTEV Plus in a 12 ml reaction volume. The reaction was incubated overnight at 30 °C. The cleaved protein was purified by application to a HisTrap HP column and collection of the flow-through. Cleavage was confirmed by SDS–PAGE and staining using Coomassie blue.

### DLS

For dynamic light scattering (DLS) measurements, the proteins were purified as mentioned previously and desalted using a HiLoad 16/600 Superdex 200 pg column (Cytiva) with 20 mM Tris–HCl pH 7.5 buffer as an eluent the day before the experiment. Buffers were filtered through Anatop 0.02 μm filters (Whatman) were used for preparation of different protein concentrations. On the day of the experiment, the proteins were concentrated to 15–30 mg ml^−1^ concentration using Amicon Ultra Centrifugal filters (Merck) via short (2–5 min) cycles at the speed ≤3,000*g* at 20 °C, and then centrifuged for 60–90 min at 17,000*g* at room temperature to remove any pre-formed aggregates in solution.

An ALV/CGS-3 goniometer with a HeNe laser operating at a wavelength of 632.8 nm, an optical fibre based detector and an ALV/LSE-5004 Light Scattering Electronics and Multiple Tau Digital Correlator were used for DLS measurements running ALV for Windows (3.04.11). The temperature was kept constant at 20 °C during data acquisition using a Thermo Scientific DC30-K20 water bath connected to the instrument and measured with a Pt-100 probe immersed into the index matching fluid vat. DLS measurements were carried out for 30–60 min at a scattering angle of 90 °C at each protein concentration. The protein concentration was determined for the sample after the last measurement using Cary-100 (Agilent) UV–visible spectrometer based on the extinction coefficients calculated by the ExPASy Server^74^.

Volume fraction is calculated using the expression *c* = *ϕ* × *n* where *c* is the concentration in mg ml^−1^, *ϕ* is the volume fraction and *n* is the partial specific volume equal to 7.266 × 10^−4^ and 7.326 × 10^−4^ ml mg^−1^ for HERD-2.2–GFP and GFP, respectively, as calculated using sedfit software^75^.

### Cloud-point measurements

Measurement of the binodal phase boundary was performed in a PerkinElmer Lambda 35 UV/Vis spectrophotometer running UVWinLab (5.1) with a temperature-controlled cuvette holder regulated by an external circulating water bath. Measurements were performed at 125 mM NaCl, 4% PEG 3350 and 20 mM Tris–HCl pH 7.5, with varying concentrations of HERD-2.2–GFP (2.7–37 mg ml^−1^). Samples were filtered using a 0.2 μm filter and incubated at 40 °C in an incubator to maintain a single phase before measurement. For each sample concentration, solution temperature was measured using a thermocouple in the reference cuvette. Phase separation was monitored by transmission (%*T*) at 600 nm as the temperature was decreased from 40 °C to 5 °C and *T*_cloud_ identified as the 50% transmission point. After %*T* stabilized, the temperature was returned to 40 °C and *T*_clear_ identified as the 50% transmission point. The threshold temperature for LLPS at that concentration was calculated as the mean of *T*_cloud_ and *T*_clear_.

### FRAP

FRAP was performed using a Leica SP8 AOBS confocal with a 65 mW Ar laser exciting at 488 nm at 22 °C. For each bleaching measurement three images were taken before bleaching, and the mean intensity was recorded as the pre-bleach fluorescence intensity. Bleaching was performed using a 100 ms (in vitro) or 1 ms (in cell) laser burst at 40% laser power, followed by imaging every 0.65 s for 20–30 s to record fluorescence recovery. Data analysis was performed in Python. The fluorescence intensity of the background was subtracted from all measurements. For each bleaching measurement, recovery was normalized relative to the mean fluorescence intensity before bleaching (normalized to 1), and the minimum fluorescence intensity measured immediately after bleaching (normalized to 0) to allow comparison between different bleaching experiments. To account for bleaching during measurements, bleaching effects were normalized to a reference droplet (or a non-bleached area of the same droplet for case where the droplets were too large). For in vitro measurements, de-mixed droplets were placed on a clean glass slide and covered with a cover slip before imaging. In vitro conditions were 33 mg ml^−1^ HERD-2.2–GFP, 125 mM NaCl, 20 mM Tris–HCl pH 7.5 and 4% or 10% PEG 3350. For in-cell measurements, FRAP was performed on live *E. coli* cells prepared as described under in-cell confocal microscopy. Cells grown at 33 °C or 18 °C were maintained at the target temperatures using a temperature-controlled chamber up until imaging, and then imaged immediately at room temperature. For cells grown at 37 °C, cells were chilled by placing at 4 °C for 1 min to induce condensation followed by imaging at room temperature. Normalized FRAP data were fitted in OriginPro to an exponential model *f*(*t*) = *A* × (1 − *e*^−*τt*^), where *A* is the plateau intensity, *τ* is the fitted parameter, and *t* is the time after bleaching. Half-lives were determined using the formula: *t*_1/2_ = ln(0.5)/*τ*.

### TC-ReAsH II labelling

TC-tagged proteins (CCPGCC) were site-specifically labelled using the TC-ReAsH II TC detection dye (Invitrogen). TC-HERD-2.2–TnaA and TC-HERD-2.2–FMO (100 µM) were incubated separately with 1 µM TC-ReAsH II and 1 mM TCEP for 1 h in the dark, before mixing with 2 mM HERD-2.2–GFP. The mixture was phase separated by addition of buffer containing 8% PEG 3350, 250 mM NaCl and 20 mM Tris–HCl pH 7.5 and droplets formed imaged at 488 nm (GFP) and 561 nm (TC-ReAsH) on a Leica SP8 confocal microscope. For normalization of fluorescence intensity, HERD-2.2–GFP droplets containing ReAsH dye incubated in the same manner, but without TC-tagged proteins, were imaged alongside each sample and the fluorescence intensity within the droplets subtracted as background.

### In-cell indigo production

Indigo production in cells expressing synthetic genes for TnaA and FMO was performed using ∆tnaa BL21 (DE3) *E. coli* generously provided by Dr Chong Zhang^76^. ∆tnaa *E. coli* were co-transformed with two vectors: one encoding the relevant HERD-GFP protein under the control of the T7 promoter (AmpR), and a second duet-style expression vector encoding both the relevant HERD-TnaA and HERD-FMO proteins under the control of the arabinose promoter (CmR). Fifty millilitres of LB was inoculated 1:100 with overnight culture and grown at 37 °C to OD_700_ of 0.4 – 0.6. Cultures were induced with 400 µM isopropyl β-d-1-thiogalactopyranoside and varying concentrations of d-arabinose and grown at 18 °C or 33 °C, shaking at 200 rpm for 22 h. To measure relative indigo production between samples, indigo concentration was first measured by absorbance at 610 nm. Two millilitres of culture was pelleted by centrifugation (3,000*g*, 5 min). The cell pellet was resuspended in *N*-methyl-2-pyrrolidone and sonicated to dissolve the indigo. Solutions were centrifuged (13,000*g*, 3 min) to remove cell debris, and indigo concentration measured by absorbance at 610 nm on a PerkinElmer Lambda 25 UV/Vis spectrophotometer. The indigo concentration was then normalized to the cell density of the culture at the time of collection, measured by absorbance at 700 nm (OD_700_) to avoid discrepancies due to the absorbance spectrum of indigo^77^. Finally, the amount of indigo was also normalized for the concentration of FMO expressed between the different fusions, as measured by western blotting. FMO was used for normalization because the oxidation of indole to indoxyl is the rate-limiting step in this reaction, with a *k*_cat_/*K*_M_ of 7.8 × 10^3^ M^−1^ s^−1^ compared with the 2.7 × 10^4^ M^−1^ s^−1^ of TnaA^78,79^. Samples for western blotting were collected and normalized to cell density to ensure equal protein concentrations. Western blots were performed as described above, and blotted against the His epitope-labelled FMO and TnaA enzymes. Quantification of enzyme expression from western blots was performed in Image Studio Lite against triplicate cultures. Background subtraction used a 3-point top-and-bottom subtraction around the band of interest to subtract non-specific background particularly visible in the HERD samples. The final relative indigo production was reported relative to the amount of indigo produced by the free enzymes, which was set as 1.

### Reporting summary

Further information on research design is available in the Nature Portfolio Reporting Summary linked to this article.

## Online content

Any methods, additional references, Nature Portfolio reporting summaries, source data, extended data, supplementary information, acknowledgements, peer review information; details of author contributions and competing interests; and statements of data and code availability are available at 10.1038/s41557-023-01321-y.

### Supplementary information

Supplementary Information Supplementary Tables 1–3 and Figs. 1–44.

Reporting Summary

Supplementary Video 1 Temperature-induced LLPS of HERD-2.2–GFP.

Supplementary Video 2 Reversible phase transition of HERD-2.2–GFP.

## Data Availability

All raw data associated with this study are available from the Zenodo repository at 10.5281/zenodo.7199035 ref. ^80^. The DeepBacs *E. coli* dataset is available at the Zenodo repository at 10.5281/zenodo.5550935 ref. ^81^.

## Code availability

Additional files used for cell and foci detection are available from the Zenodo repository at 10.5281/zenodo.6949385 ref. ^63^. Code related to the modular image analysis (MIA) plugin is available from the Zenodo repositories at 10.5281/zenodo.6832092 and 10.5281/zenodo.6907671 ref. ^62^.

## References

[1] Benedek GB (1997). Cataract as a protein condensation disease: the Proctor Lecture. Investig. Ophthalmol. Vis. Sci..

[2] Brangwynne CP, Mitchison TJ, Hyman AA (2011). Active liquid-like behavior of nucleoli determines their size and shape in *Xenopus laevis* oocytes. Proc. Natl Acad. Sci. USA.

[3] Boeynaems S (2018). Protein phase separation: a new phase in cell biology. Trends Cell Biol..

[4] Alberti S, Gladfelter A, Mittag T (2019). Considerations and challenges in studying liquid–liquid phase separation and biomolecular condensates. Cell.

[5] McManus JJ, Charbonneau P, Zaccarelli E, Asherie N (2016). The physics of protein self-assembly. Curr. Opin. Colloid Interface Sci..

[6] Schramm FD, Schroeder K, Jonas K (2020). Protein aggregation in bacteria. FEMS Microbiol. Rev..

[7] Rana U, Brangwynne CP, Panagiotopoulos AZ (2021). Phase separation vs aggregation behavior for model disordered proteins. J. Chem. Phys..

[8] Villegas JA, Heidenreich M, Levy ED (2022). Molecular and environmental determinants of biomolecular condensate formation. Nat. Chem. Biol..

[9] Sridharan S (2022). Systematic discovery of biomolecular condensate-specific protein phosphorylation. Nat. Chem. Biol..

[10] Antifeeva IA (2022). Liquid–liquid phase separation as an organizing principle of intracellular space: overview of the evolution of the cell compartmentalization concept. Cell. Mol. Life Sci..

[11] Alberti S, Hyman AA (2021). Biomolecular condensates at the nexus of cellular stress, protein aggregation disease and ageing. Nat. Rev. Mol. Cell Biol..

[12] Qian Z-G, Huang S-C, Xia X-X (2022). Synthetic protein condensates for cellular and metabolic engineering. Nat. Chem. Biol..

[13] Schuster BS (2018). Controllable protein phase separation and modular recruitment to form responsive membraneless organelles. Nat. Commun..

[14] Reinkemeier CD, Girona GE, Lemke EA (2019). Designer membraneless organelles enable codon reassignment of selected mRNAs in eukaryotes. Science.

[15] Dzuricky M, Rogers BA, Shahid A, Cremer PS, Chilkoti A (2020). De novo engineering of intracellular condensates using artificial disordered proteins. Nat. Chem..

[16] Heidenreich M (2020). Designer protein assemblies with tunable phase diagrams in living cells. Nat. Chem. Biol..

[17] Yoshikawa M, Yoshii T, Ikuta M, Tsukiji S (2021). Synthetic protein condensates that inducibly recruit and release protein activity in living cells. J. Am. Chem. Soc..

[18] Niu J, Qiu C, Abbott NL, Gellman SH (2022). Formation of versus recruitment to RNA-rich condensates: controlling effects exerted by peptide side chain identity. J. Am. Chem. Soc..

[19] Huang PS, Boyken SE, Baker D (2016). The coming of age of de novo protein design. Nature.

[20] Beesley JL, Woolfson DN (2019). The de novo design of α-helical peptides for supramolecular self-assembly. Curr. Opin. Biotechnol..

[21] Lapenta F, Aupič J, Strmšek Ž, Jerala R (2018). Coiled coil protein origami: from modular design principles towards biotechnological applications. Chem. Soc. Rev..

[22] Lee MJ (2018). Engineered synthetic scaffolds for organizing proteins within the bacterial cytoplasm. Nat. Chem. Biol..

[23] Lin D (2023). Time-tagged ticker tapes for intracellular recordings. Nat. Biotechnol..

[24] Linghu C (2023). Recording of cellular physiological histories along optically readable self-assembling protein chains. Nat. Biotechnol..

[25] Olshefsky A, Richardson C, Pun SH, King NP (2022). Engineering self-assembling protein nanoparticles for therapeutic delivery. Bioconjug. Chem..

[26] Fletcher JM (2012). A basis set of de novo coiled-coil peptide oligomers for rational protein design and synthetic biology. ACS Synth. Biol..

[27] Dawson WM (2021). Coiled coils 9-to-5: rational de novo design of α-helical barrels with tunable oligomeric states. Chem. Sci..

[28] Fletcher JM (2013). Self-assembling cages from coiled-coil peptide modules. Science.

[29] Smith AJ, Thomas F, Shoemark D, Woolfson DN, Savery NJ (2019). Guiding biomolecular interactions in cells using de novo protein–protein interfaces. ACS Synth. Biol..

[30] Zhang Q (2018). Visualizing dynamics of cell signaling in vivo with a phase separation-based kinase reporter. Mol. Cell.

[31] Lasker K (2022). The material properties of a bacterial-derived biomolecular condensate tune biological function in natural and synthetic systems. Nat. Commun..

[32] Gomes E, Shorter J (2019). The molecular language of membraneless organelles. J. Biol. Chem..

[33] Woolfson DN (2017). Coiled-coil design: updated and upgraded. Subcell Biochem..

[34] Woolfson DN (2021). A brief history of de novo protein design: minimal, rational, and computational. J. Mol. Biol..

[35] Petka WA, Harden JL, McGrath KP, Wirtz D, Tirrell DA (1998). Reversible hydrogels from self-assembling artificial proteins. Science.

[36] Peran I, Mittag T (2020). Molecular structure in biomolecular condensates. Curr. Opin. Struct. Biol..

[37] Edgell CL, Savery NJ, Woolfson DN (2020). Robust de novo-designed homotetrameric coiled coils. Biochemistry.

[38] Theillet F-X (2013). The alphabet of intrinsic disorder. Intrinsically Disord. Proteins.

[39] Harmon TS, Holehouse AS, Rosen MK, Pappu RV (2017). Intrinsically disordered linkers determine the interplay between phase separation and gelation in multivalent proteins. eLife.

[40] Erdős G, Pajkos M, Dosztányi Z (2021). IUPred3: prediction of protein disorder enhanced with unambiguous experimental annotation and visualization of evolutionary conservation. Nucleic Acids Res..

[41] Koga S, Williams DS, Perriman AW, Mann S (2011). Peptide–nucleotide microdroplets as a step towards a membrane-free protocell model. Nat. Chem..

[42] Schavemaker PE, Śmigiel WM, Poolman B (2017). Ribosome surface properties may impose limits on the nature of the cytoplasmic proteome. eLife.

[43] Cubitt AB, Woollenweber LA, Heim R (1998). Chapter 2: understanding structure—function relationships in the Aequorea victoria green fluorescent protein. Methods Cell. Biol..

[44] Pace CN, Scholtz JM (1998). A helix propensity scale based on experimental studies of peptides and proteins. Biophys. J..

[45] Dong H, Hartgerink JD (2006). Short homodimeric and heterodimeric coiled coils. Biomacromolecules.

[46] André AAM, Spruijt E (2020). Liquid–liquid phase separation in crowded environments. Int. J. Mol. Sci..

[47] Muschol M, Rosenberger F (1995). Interactions in undersaturated and supersaturated lysozyme solutions: static and dynamic light scattering results. J. Chem. Phys..

[48] McManus JJ (2007). Altered phase diagram due to a single point mutation in human γD-crystallin. Proc. Natl Acad. Sci. USA.

[49] van den Berg B, Ellis RJ, Dobson CM (1999). Effects of macromolecular crowding on protein folding and aggregation. EMBO J..

[50] Buck M (1998). Trifluoroethanol and colleagues: cosolvents come of age. Recent studies with peptides and proteins. Q. Rev. Biophys..

[51] Leonard DJ (2021). Scalable synthesis and coupling of quaternary α-arylated amino acids: α-aryl substituents are tolerated in α-helical peptides. Chem. Sci..

[52] Myhrvold C, Polka JK, Silver PA (2016). Synthetic lipid-containing scaffolds enhance production by colocalizing enzymes. ACS Synth. Biol..

[53] Das Subash C, Panda D, Nayak D, Pattnaik Asit K (2009). Biarsenical labeling of vesicular stomatitis virus encoding tetracysteine-tagged M protein allows dynamic imaging of M protein and virus uncoating in infected cells. J. Virol..

[54] Castellana M (2014). Enzyme clustering accelerates processing of intermediates through metabolic channeling. Nat. Biotechnol..

[55] Yoshikawa M, Tsukiji S (2021). Modularly built synthetic membraneless organelles enabling targeted protein sequestration and release. Biochemistry.

[56] Garabedian MV (2022). Protein condensate formation via controlled multimerization of intrinsically disordered sequences. Biochemistry.

[57] Reinkemeier CD, Lemke EA (2021). Dual film-like organelles enable spatial separation of orthogonal eukaryotic translation. Cell.

[58] Guan M (2021). Incorporation and assembly of a light-emitting enzymatic reaction into model protein condensates. Biochemistry.

[59] Gasteiger, E. et al. In *The Proteomics Protocols Handbook* (ed. Walker, J. M.) 571–607 (Humana Press, 2005).

[60] Schindelin J (2012). Fiji: an open-source platform for biological-image analysis. Nat. Methods.

[61] Schneider CA, Rasband WS, Eliceiri KW (2012). NIH Image to ImageJ: 25 years of image analysis. Nat. Methods.

[62] Cross, S. J. ModularImageAnalysis (MIA) (1.0.3). *Zenodo*10.5281/zenodo.6832092 (2022).

[63] Hilditch, A. T. et al. Additional files for cell and foci detection and analysis (1.0.0). *Zenodo*10.5281/zenodo.6949385 (2022).

[64] Umorin, M. Stack focuser. *ImageJ* https://imagej.nih.gov/ij/plugins/stack-focuser.html (2022).

[65] Schmidt U, Weigert M, Broaddus C, Myers G (2018). Cell detection with star-convex polygons. Medical Image Computing and Computer Assisted Intervention – MICCAI.

[66] Weigert M (2018). Content-aware image restoration: pushing the limits of fluorescence microscopy. Nat. Methods.

[67] Cross, S. J. MIA DeepLearning 1.0.0. *Zenodo*10.5281/zenodo.6907671 (2021).

[68] Spahn C (2022). DeepBacs for multi-task bacterial image analysis using open-source deep learning approaches. Commun. Biol..

[69] Tinevez J-Y (2017). TrackMate: an open and extensible platform for single-particle tracking. Methods.

[70] Legland D, Arganda-Carreras I, Andrey P (2016). MorphoLibJ: integrated library and plugins for mathematical morphology with ImageJ. Bioinformatics.

[71] Gill SC, von Hippel PH (1989). Calculation of protein extinction coefficients from amino acid sequence data. Anal. Biochem..

[72] Kuipers BJH, Gruppen H (2007). Prediction of molar extinction coefficients of proteins and peptides using UV absorption of the constituent amino acids at 214 nm to enable quantitative reverse phase high-performance liquid chromatography−mass spectrometry analysis. J. Agric. Food Chem..

[73] Myers JK, Pace CN, Scholtz JM (1997). A direct comparison of helix propensity in proteins and peptides. Proc. Natl Acad. Sci. USA.

[74] Wilkins, M. R. et al. In *2-D Proteome Analysis Protocols* (ed. Link, A. J.) 531–552 (Humana Press, 1999).

[75] Schuck P (2000). Size-distribution analysis of macromolecules by sedimentation velocity ultracentrifugation and Lamm equation modeling. Biophys. J..

[76] Fang M-Y (2015). High crude violacein production from glucose by *Escherichia coli* engineered with interactive control of tryptophan pathway and violacein biosynthetic pathway. Microb. Cell Factories.

[77] Seixas de Melo J, Moura AP, Melo MJ (2004). Photophysical and spectroscopic studies of indigo derivatives in their keto and leuco forms. J. Phys. Chem. A.

[78] Alfieri A, Malito E, Orru R, Fraaije MW, Mattevi A (2008). Revealing the moonlighting role of NADP in the structure of a flavin-containing monooxygenase. Proc. Natl Acad. Sci. USA.

[79] Harris AP, Phillips RS (2013). Benzimidazole analogs of l-tryptophan are substrates and inhibitors of tryptophan indole lyase from *Escherichia coli*. FEBS J..

[80] Hilditch, A. T. et al. Data associated with the publication ‘Assembling membraneless organelles from de novo designed proteins’. *Zenodo*10.5281/zenodo.7199035 (2023).

[81] Spahn, C. & Heilemann, M. DeepBacs—*Escherichia coli* bright field segmentation dataset. *Zenodo*. 10.5281/zenodo.5550935 (2021).

