# Supplementary material for: Assembling membraneless organelles from de novo designed proteins
Source: Nat Chem. 2023 Sep 14;16(1):89–97. doi: 10.1038/s41557-023-01321-y (PMC10774119; doi:10.1038/s41557-023-01321-y)
Supplement: Supplementary file 1 — Supplementary Tables 1–3 and Figs. 1–44. [file 41557_2023_1321_MOESM1_ESM.pdf]

nature chemistry

Article

https://doi.org/10.1038/s41557-023-01321-y

Assembling membraneless organelles from de novo designed proteins

In the format provided by the authors and unedited

Table of contents

Supplementary Table S1: Sequences of HERD tags. 3
Supplementary Table S2: Graphical representation of HERD tags. 5
Supplementary Table S3: Chemically synthesised peptides. 6
Supplementary Fig. S1: Design of the unstructured linker. 7
Supplementary Fig. S2: Confocal microscopy images of HERD-0-GFP expressed in *E. coli*. 7
Supplementary Fig. S3: Confocal microscopy images of mEmerald and His-TEV-mEmerald expressed in *E. coli*. 8
Supplementary Fig. S4: Western blot of HERD-0-GFP from *E. coli* cell lysates. 8
Supplementary Fig. S5: Confocal microscopy images of HERD-1.1 expressed in *E. coli*. 9
Supplementary Fig. S6: Western blot of HERD-1.1-GFP from *E. coli* cell lysates. 9
Supplementary Fig. S7: Confocal microscopy images of HERD-2.1 through HERD-2.4 expressed in *E. coli*. 10
Supplementary Fig. S8: Western blots of HERD-2.1 through HERD-2.4 fusions from *E. coli* cell lysates. 11
Supplementary Fig. S9: Confocal microscopy images of HERD-2.5 through HERD-2.8 expressed in *E. coli*. 12
Supplementary Fig. S10: Confocal microscopy images of HERD-Ctrl1 and HERD-Ctrl2 expressed in *E. coli*. 13
Supplementary Fig. S11: Western blots of HERD-2 .5 through HERD-2.8 fusions from *E. coli* cell lysates. 14
Supplementary Fig. S12: Western blots of HERD-Ctrl1 and HERD-Ctrl2 fusions from *E. coli* cell lysates. 14
Supplementary Fig. S13: Designed linker variations. 15
Supplementary Fig. S14: Confocal microscopy images of HERD-3.1 through HERD-3.4 expressed in *E. coli*. 16
Supplementary Fig. S15: Automated image analysis of HERD–GFP fusions from confocal microscopy. 17
Supplementary Fig. S16: Temperature-controlled live-cell imaging of HERD-2.2–GFP expressed in *E. coli*. 18
Supplementary Fig. S17: SDS-PAGE gel of purified HERD-2.2–GFP. 19
Supplementary Fig. S18: Image based buffer screen for phase separation of HERD-2.2–GFP. 20
Supplementary Fig. S19: Confocal microscopy images of HERD-2.2-GFP droplets *in vitro*. 21
Supplementary Fig. S20: Coalescence of HERD-2.2-GFP droplets. 22
Supplementary Fig. S21: SDS-PAGE of HERD-2.2-GFP before and after TEV cleavage. 23
Supplementary Fig. S22: Confocal microscopy of HERD-2.2-GFP droplets *in vitro* after TEV cleavage 23
Supplementary Fig. S23: Circular dichroism spectroscopy of HERD-2.2-GFP. 24
Supplementary Fig. S24: Analytical HPLC traces of synthesised peptides 1-10. 25
Supplementary Fig. S25. MALDI-TOF MS spectra for peptides 1-9 and ESI MS spectrum for peptide 10. 26
Supplementary Fig. S26. Circular dichroism spectra for the chemically synthesised peptides 1 – 10. 28
Supplementary Fig. S27: CD spectra for peptide 10 at different PEG 3350 concentrations. 29
Supplementary Fig. S28: CD spectra for the peptides 4 and 8 – 10 at 100 µM in TFE (0 – 90%). 30
Supplementary Fig. S29: Automated image analysis of helix destabilising HERD-GFP constructs. 31
Supplementary Fig. S30: Cloud-point measurements for HERD-2.2–GFP *in vitro* with respect to temperature. 32
Supplementary Fig. S31: Asymmetrical bleaching of HERD-0–GFP and HERD-2.2–GFP condensates in cells. 33
Supplementary Fig. S32: Confocal microscopy images of HERD-2.2–GFP co-expressed with HERD-2.2–mCherry. 34
Supplementary Fig. S33: SDS-PAGE of HERD-2.2 fusions with TnaA and FMO. 35
Supplementary Fig. S34: Brightfield transmission images of de-mixed droplets formed by HERD-2.2–TnaA. 35
Supplementary Fig. S35: In vitro confocal microscopy images of enriched TC tagged HERD-2.2 fusions. 36

Supplementary Fig. S36: Quantification of enriched TC tagged HERD-2.2 fusions. 37
Supplementary Fig. S37: Confocal microscopy images of TnaA and FMO HERD constructs co-expressed in *E. coli*. 38
Supplementary Fig. S38: Quantification of enzyme expression and indigo production of HERD-2.2. 39
Supplementary Fig. S39: Microscopy of HERD-2.2–GFP with increasing HERD-2.2–FMO and HERD-2.2–TnaA. 40
Supplementary Fig. S40: Quantification of enzyme expression and indigo production at 33 °C. 41
Supplementary Fig. S41: Quantification of enzyme expression and indigo production at 18 °C. 42
Supplementary Fig. S42: FRAP of HERD-2.2-GFP droplets in cells under different growth conditions. 43
Supplementary Fig. S43: Quantification of enzyme expression and indigo production....... 44
Supplementary Fig. S44: pDIC (design in cells) vectors used for protein expression. 45
Unprocessed scans of blots and gels for supplementary figures 46

HERD #	Helical region 1 (HR1)<br>*gabcdef gabcdef gabcdef gabcdef*	Linker	Helical region 2 (HR2)<br>*cdefgab cdefgab cdefgab cdefgab*
0	G EIAAIKE EIAAIKE EIAAIKW EIAAIKE G	ASPEPQPKPSGDPQSKQTPEPSRSQ	G EIQEQLK EIQEQLK EIQWQLK EIQEQLK G
1.1	G EIAAIKE EIAAIKW EIAAIKE G	ASPEPQPKPSGDPQSKQTPEPSRSQ	G EIQEQLK EIQWQLK EIQEQLK G
2.1	G EAAAIKE EAAAIKW EAAAIKE G	ASPEPQPKPSGDPQSKQTPEPSRSQ	G EIQEQAK EIQWQAK EIQEQAK G
2.2	G IKE EAAAIKW EAAAIKE G	ASPEPQPKPSGDPQSKQTPEPSRSQ	G QAK EIQWQAK EIQEQAK G
2.3	G EISSIKE EISSIKW EISSIKE G	ASPEPQPKPSGDPQSKQTPEPSRSQ	G EIQEQLK EIQWQLK EIQEQLK G
2.4	G EISSIKE EISSIKW EASSIKE G	ASPEPQPKPSGDPQSKQTPEPSRSQ	G EIQEQLK EIQWQLK EIQEQAK G
2.5	G EAAAIKW EAAAIKE G	ASPEPQPKPSGDPQSKQTPEPSRSQ	G EIQWQAK EIQEQAK G
2.6	G EASSIKE EASSIKE EASSIKW EASSIKE G	ASPEPQPKPSGDPQSKQTPEPSRSQ	G EIQEQAK EIQEQAK EIQWQAK EIQEQAK G
2.7	G EISSIKE EASSIKW EASSIKE G	ASPEPQPKPSGDPQSKQTPEPSRSQ	G EIQEQLK EIQWQAK EIQEQAK G
2.8	G EASSIKE EASSIKW EASSIKE G	ASPEPQPKPSGDPQSKQTPEPSRSQ	G EIQEQAK EIQWQAK EIQEQAK G
3.1	G IKE EAAAIKW EAAAIKE G	ASPEPQPKPSGDPQSKQTPEASPRPQ<br>PEPSGKPQSEQTPKASPEPQPKPS	G QAK EIQWQAK EIQEQAK G
3.2	G IKE EAAAIKW EAAAIKE G	ASESQKPS	G QAK EIQWQAK EIQEQAK G
3.3	G IKE EAAAIKW EAAAIKE G	ASEPKQSDPKGDPRSEQKPEKSESR	G QAK EIQWQAK EIQEQAK G
3.4	G IKE EAAAIKW EAAAIKE G	ASPTSYPAPSWAPQYLQTPGPSVSQ	G QAK EIQWQAK EIQEQAK G
4.1	G IKQ QAAAIKW QAAAIKQ G	ASPQPQPKPSGQPQSKQTPQPSRSQ	G QAK QIQWQAK QIQQQAK G
Ctrl1	G EAAAAKE EAAAAKW EAAAAKE G	ASPEPQPKPSGDPQSKQTPEPSRSQ	G EIQEQAK EIQWQAK EIQEQAK G
Ctrl2	G EAAAAKE EAAAAKW EAAAAKE G	ASPEPQPKPSGDPQSKQTPEPSRSQ	G EAQEQAK EAQWQAK EAQEQAK G
Ctrl3	G KEI EAAAIKW EAAAKIE G	ASPEPQPKPSGDPQSKQTPEPSRSQ	G QAK IEQWQAK EEQIQAK G
Ctrl4	G PKE EAAAPKW EAAAPKE G	ASPEPQPKPSGDPQSKQTPEPSRSQ	G QAK EPQWQAK EPQEQAK G
Ctrl5	G GKE EAAAGKW EAAAGKE G	ASPEPQPKPSGDPQSKQTPEPSRSQ	G QAK EGQWQAK EGQEQAK G
Ctrl6	G IKE EAPAIKW EAPAIKE G	ASPEPQPKPSGDPQSKQTPEPSRSQ	G QAK EIQPQAK EIQEPAK G
Ctrl7	G IKE EAGAIKW EAGAIKE G	ASPEPQPKPSGDPQSKQTPEPSRSQ	G QAK EIQGQAK EIQEGAK G
TC-2.2	G IKE EAAAIKW EAAAIKE G	ASPEPQPKPSCCPGCCQTPEPSRSQ	G QAK EIQWQAK EIQEQAK G
TC-4.1	G IKQ QAAAIKW QAAAIKQ G	ASPQPQPKPCCPGCCKQTPQPSRSQ	G QAK QIQWQAK QIQQQAK G

Supplementary Table S1: Sequences of HERD tags. Sequences of the helical regions (HR1 and 2) are given in heptad registers of the parent coiled coils on which they were based.

HERD #	Structural model	Description
0	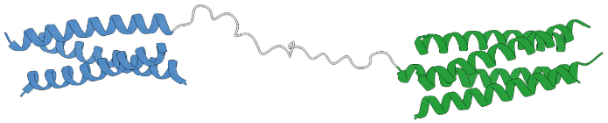	4 heptads (28 residues)<br>Original coiled coil designs
1.1	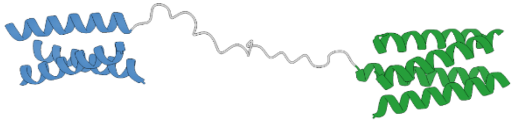	3 heptads (21 residues)
2.1	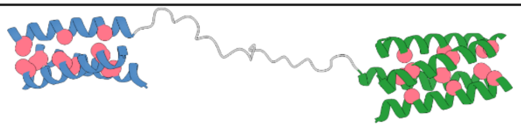	3 heptads (21 residues)<br>Ala at *a* position
2.2	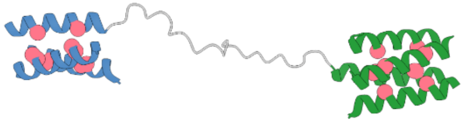	2.5 heptads (18 residues)<br>Ala at *a* position
2.3	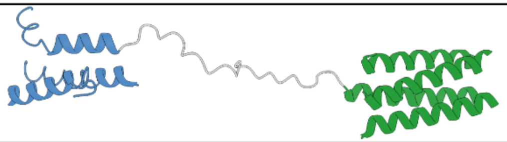	3 heptads (21 residues)<br>Low helical propensity residues in HR1
2.4	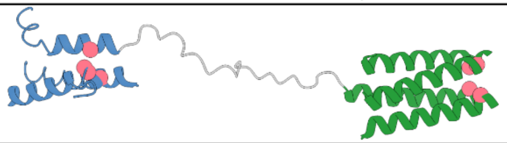	3 heptads (21 residues)<br>Low helical propensity residues in HR1<br>1 Ala at *a* position
2.5	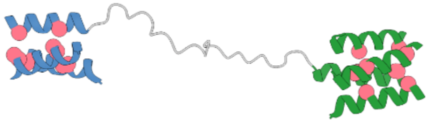	2 heptads (14 residues)<br>Ala at *a* position
2.6	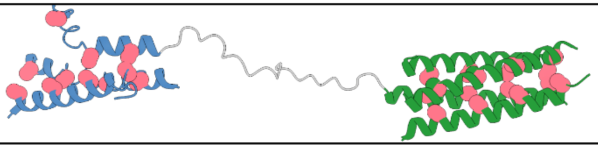	4 heptads (28 residues)<br>Low helical propensity residues in HR1<br>Ala at *a* position

HERD #	Structural model	Description
2.7	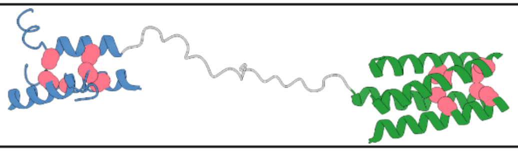	3 heptads (21 residues)<br>Low helical propensity residues in HR1<br>2 Ala at *a* position
2.8	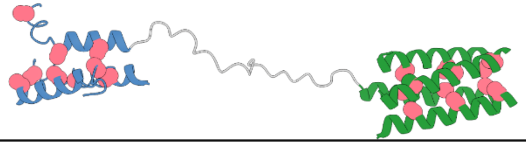	3 heptads (21 residues)<br>Low helical propensity residues in HR1<br>3 Ala at *a* position
3.1	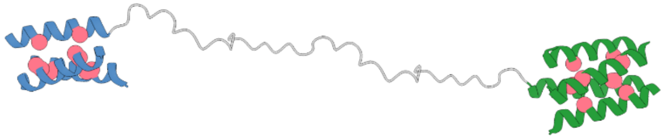	Long (50 residue) linker
3.2	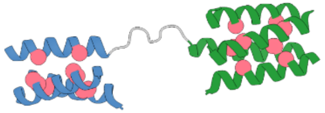	Short (8 residue) linker
3.3	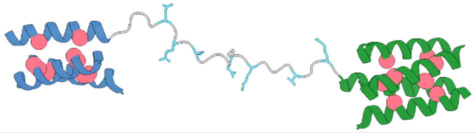	Increased ionic residue density linker
3.4	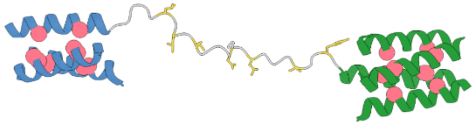	Uncharged linker
Ctrl1	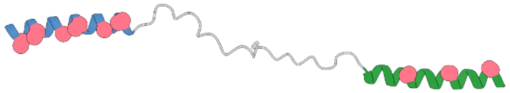	3 heptads (21 residues)<br>Ala at *a* and *d* positions in HR1<br>Ala at *a* position in HR2
Ctrl2	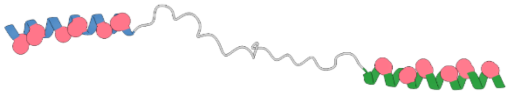	3 heptads (21 residues)<br>Ala at *a* and *d* positions in HR1 and 2

Supplementary Table S2: Graphical representation of HERD tags. Cartoon models of the main design routes of the HERD proteins with notes on their sequence compositions. HERD constructs shown relate to sequences shown in Supplementary Table S1.

#	Peptide	Sequence	m/z	
			Theoretical	Observed
**Helical region 1 (HR1)**		*gabcdef gabcdef gabcdef gabcdef*		
1	HR1-4H	Ac-G EIAAIKE EIAAIKE EIAAIKW EIAAIKE G-NH_2	3248.810	3248.785
2	HR1-A@a-4H	Ac-G EAAAIKE EAAAIKE EAAAIKW EAAAIKE G-NH_2	3080.622	3080.651
3	HR1-A@a-3H	Ac-G EAAAIKE EAAAIKW EAAAIKE G-NH_2	2368.247	2368.810
4	HR1-A@a-2.5H	Ac-G IKE EAAAIKW EAAAIKE G-NH_2	2025.089	2025.722
**Helical region 2 (HR2)**		*cdefgab cdefgab cdefgab cdefgab*		
5	HR1-4H	Ac-G EIQEQLK EIQEQLK EIQWQLK EIQEQLK G-NH2	3704.982	3705.882
6	HR1-A@a-4H	Ac-G EIQEQAK EIQEQAK EIQWQAK EIQEQAK G-NH2	3536.794	3537.772
7	HR1-A@a-3H	Ac-G EIQEQAK EIQWQAK EIQEQAK G-NH2	2710.376	2710.527
8	HR1-A@a-2.5H	Ac-G QAK EIQWQAK EIQEQAK G-NH2	2211.148	2211.857
**Linker**				
9	Linker	Ac-ASPEP QPKPS GDPQS KQTPE PSRSQ-NH2	2701.314	2701.378
10	HR1-L-HR2	Ac-G IKE EAAAIKW EAAAIKE G-<br>-ASPEP QPKPS GDPQS KQTPE PSRSQ-<br>-G QAK EIQWQAK EIQEQAK G-NH2	6821.46	6821.234

Supplementary Table S3: Chemically synthesised peptides. Sequences of the synthesized peptides and the main peaks ([M+H$^+$]) obtained by MALDI-TOF MS for the peptides 1-9 and ESI MS for the peptide 10. The heptad register is indicated above the sequences (for helical regions 1 and 2). All sequences contained *N*- and *C*-terminal glycine residues (not included in the heptad register assignment) and were *N*-terminally acylated and *C*-terminally amidated.

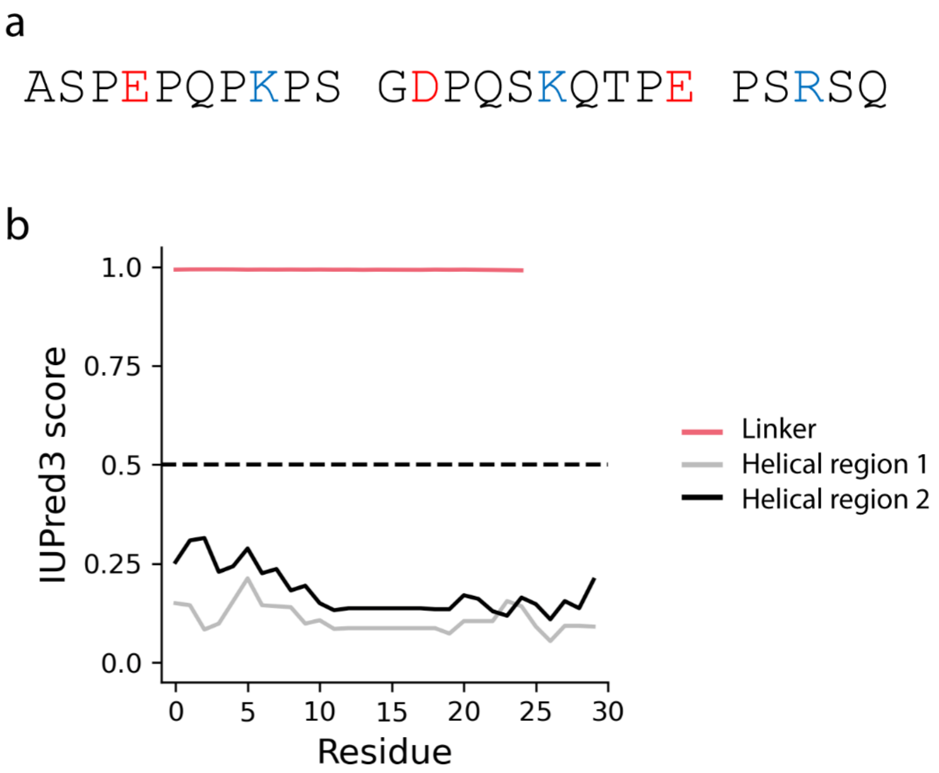

Supplementary Fig. S1: Design of the unstructured linker. a, The linker was designed with a length of 25 residues and a fraction of charged residues of 25% based on in silico data by Harmon *et al.* (2017) to improve global cooperativity between stickers. Additionally, we included the constraints that the linker should have a net-zero charge, and that charged residues (red, blue) be evenly spaced to reduce patchy ionic interactions. We required that the linker should also be unstructured, incorporating several proline and glycine residues, and excluded aromatic and arginine residues as they could form additional protein-protein or protein-RNA interactions, and are particularly implicated in LLPS. **b,** Disorder prediction of HERD-0 components by IUPred3. Probability of each of the 3 designed components of HERD-0 being part of a disordered region is given by a value between 0 and 1, with a cut-off of 0.5 given by a hashed black line. Pink, linker; grey, helical region 1; solid black, helical region 2. IUPred3 predictions were generated using long disorder parameters with no smoothing applied.

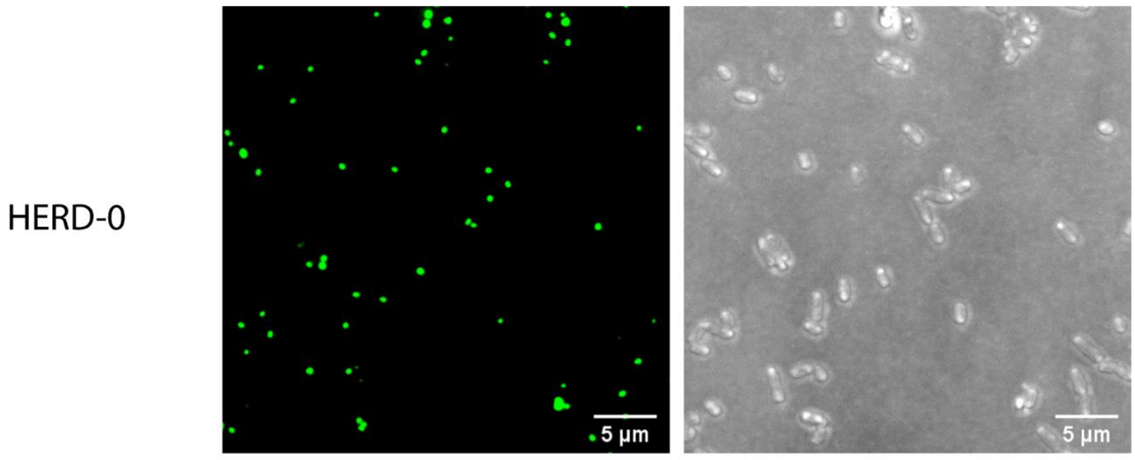

Supplementary Fig. S2: Confocal microscopy images of HERD-0-GFP expressed in *E. coli*. mEmerald fluorescence at 488 nm (green) and brightfield transmission (grey).

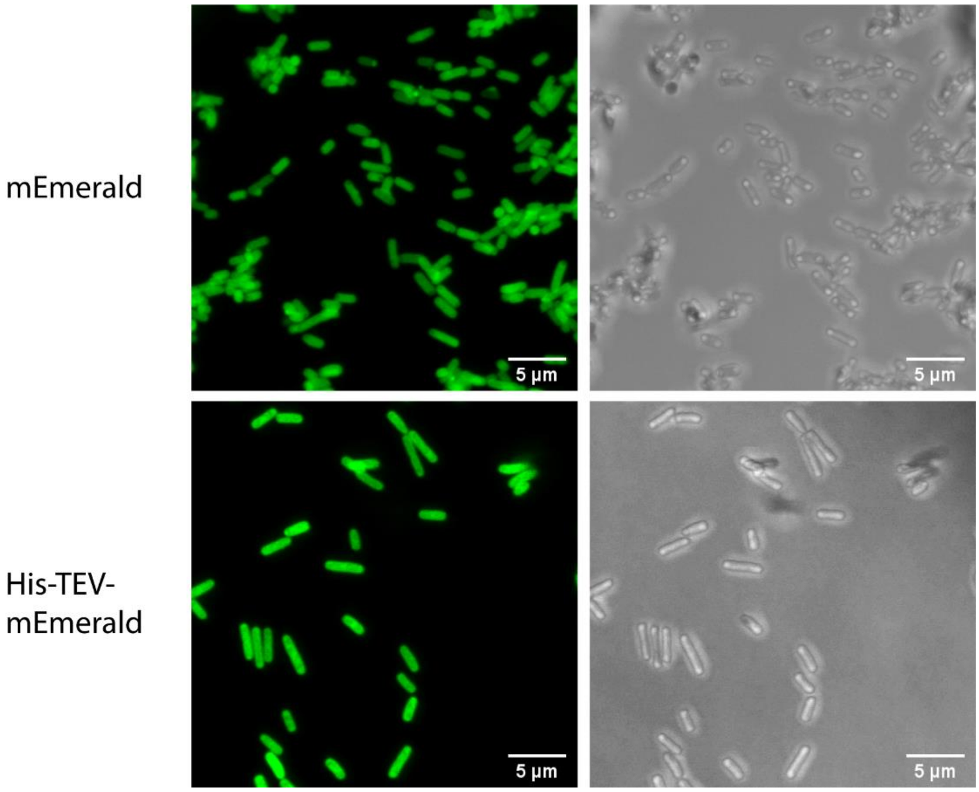

Supplementary Fig. S3: Confocal microscopy images of mEmerald and His-TEV-mEmerald expressed in *E. coli*. mEmerald fluorescence at 488 nm (green) and brightfield transmission (grey).

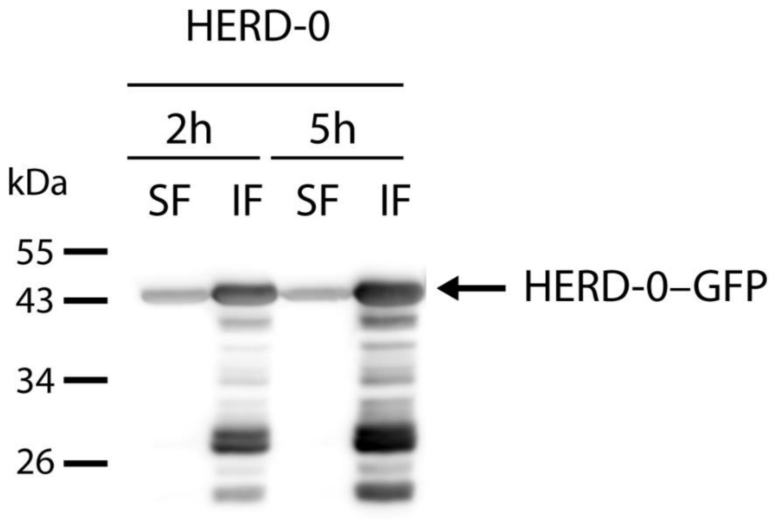

Supplementary Fig. S4: Western blot of HERD-0-GFP from *E. coli* cell lysates. Cell lysates 2 hours (2h) and 5 hours (5h) after induction were separated into their soluble fractions (SF) and insoluble fractions (IF) before analysis by SDS-PAGE and western blotting against the *N*-terminal His epitope tag. Degradation products in the IF lanes are shown below the band corresponding to HERD-0-GFP.

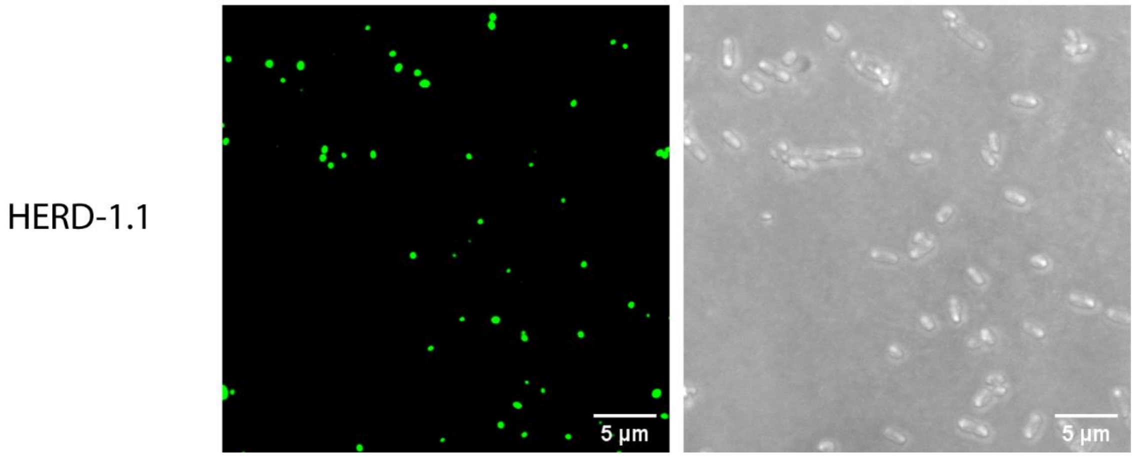

Supplementary Fig. S5: Confocal microscopy images of HERD-1.1 expressed in *E. coli*. mEmerald fluorescence at 488 nm (green) and brightfield transmission (grey).

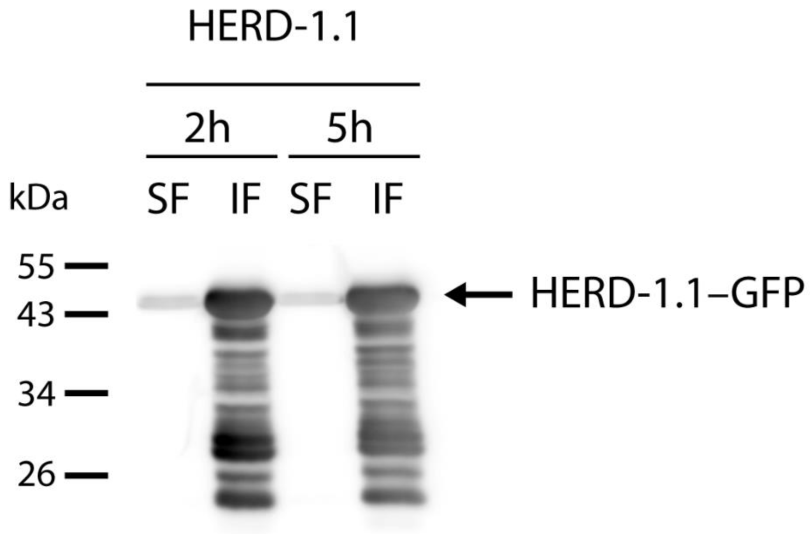

Supplementary Fig. S6: Western blot of HERD-1.1-GFP from *E. coli* cell lysates. Cell lysates 2 hours (2h) and 5 hours (5h) after induction were separated into their soluble fractions (SF) and insoluble fractions (IF) before analysis by SDS-PAGE and western blotting against the *N*-terminal His epitope tag. Degradation products in the IF lanes are shown below the band corresponding to HERD-1.1-GFP.

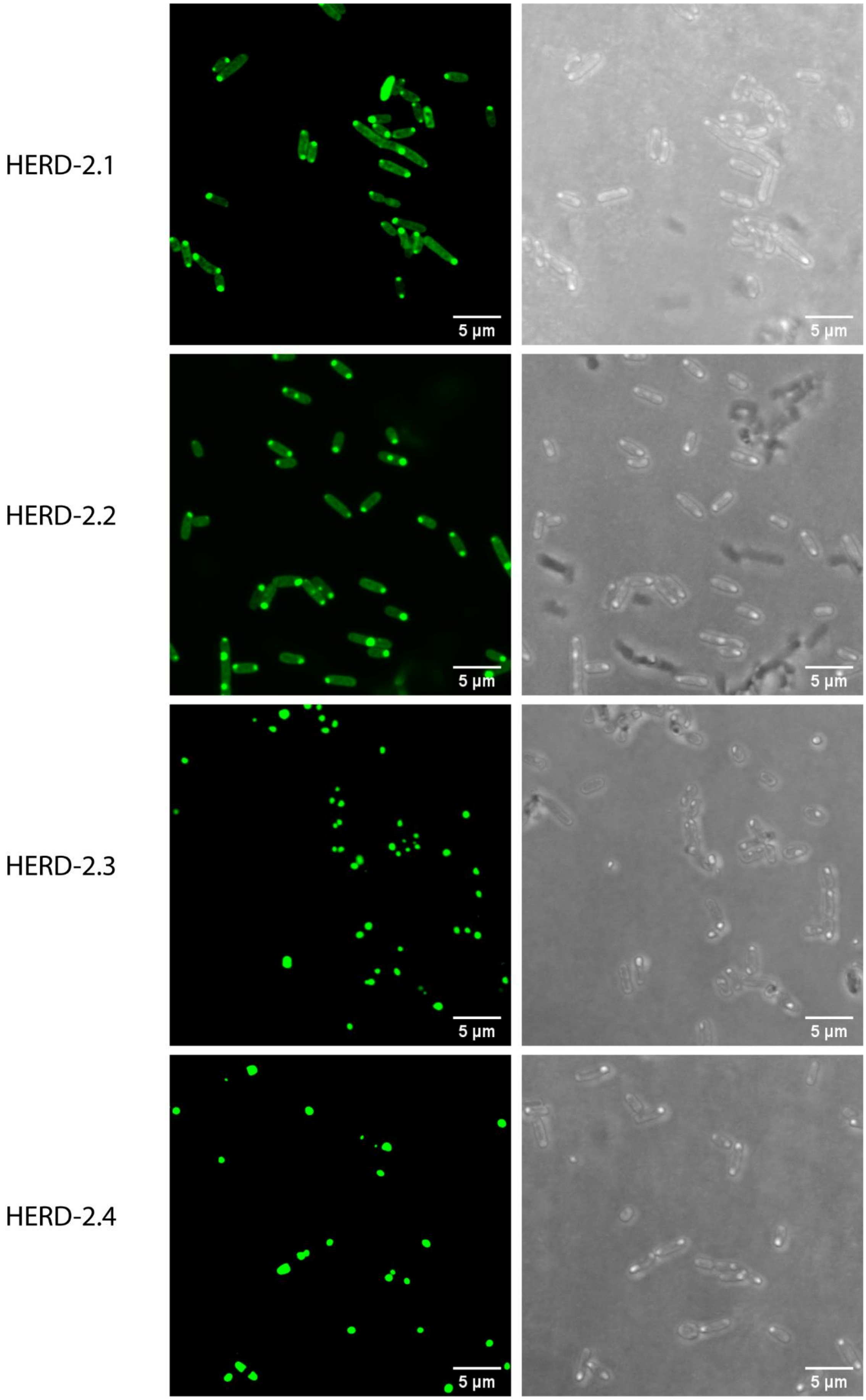

Supplementary Fig. S7: Confocal microscopy images of HERD-2.1 through HERD-2.4 expressed in *E. coli*. mEmerald fluorescence at 488 nm (green) and brightfield transmission (grey).

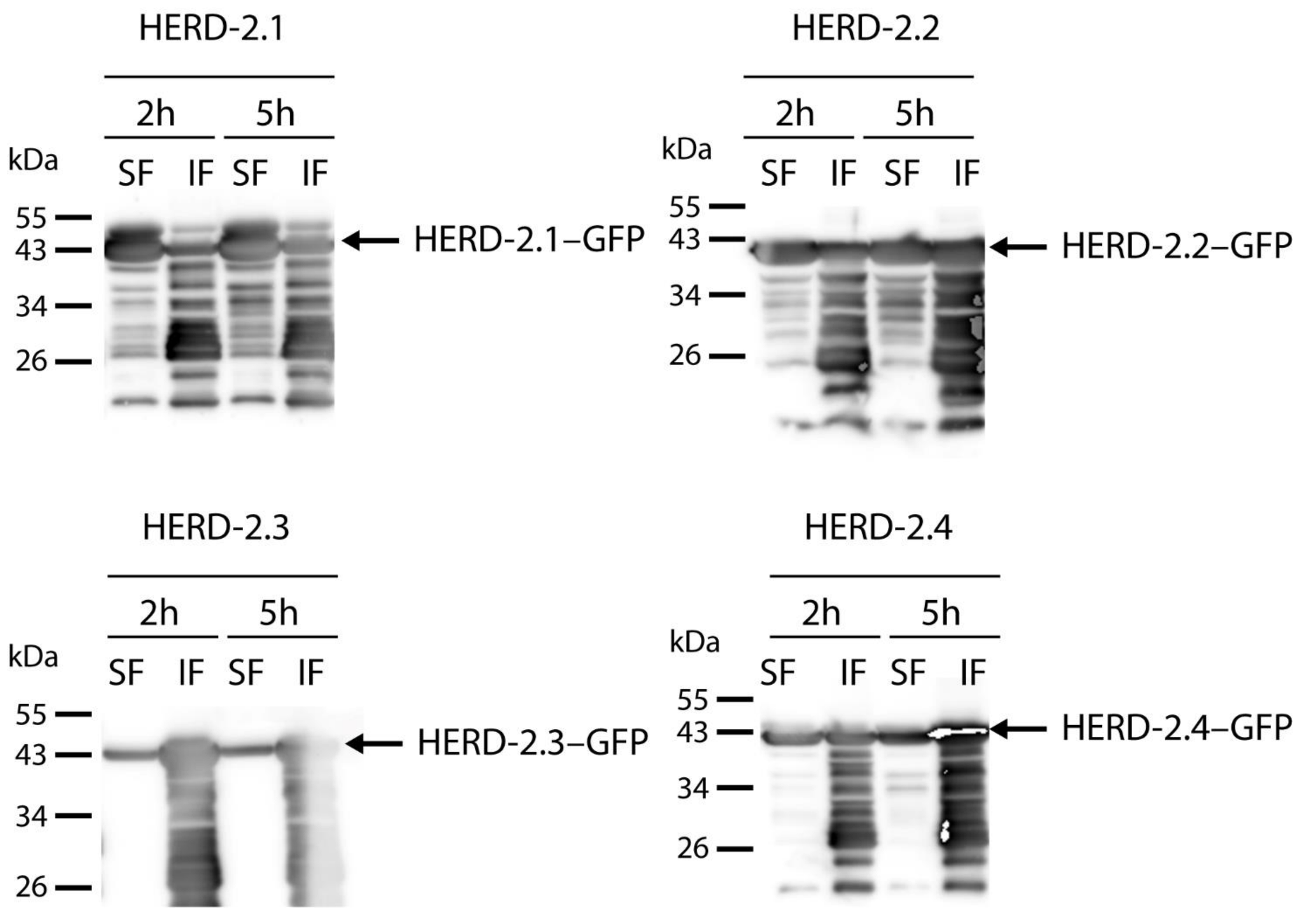

Supplementary Fig. S8: Western blots of HERD-2.1 through HERD-2.4 fusions from *E. coli* cell lysates. Cell lysates 2 hours (2h) and 5 hours (5h) after induction were separated into their soluble fractions (SF) and insoluble fractions (IF) before analysis by SDS-PAGE and western blotting against the *N*-terminal His epitope tag. Degradation products in the IF lanes, and to a lesser extent in the SF lanes are shown below the bands corresponding to the HERD-GFP fusions.

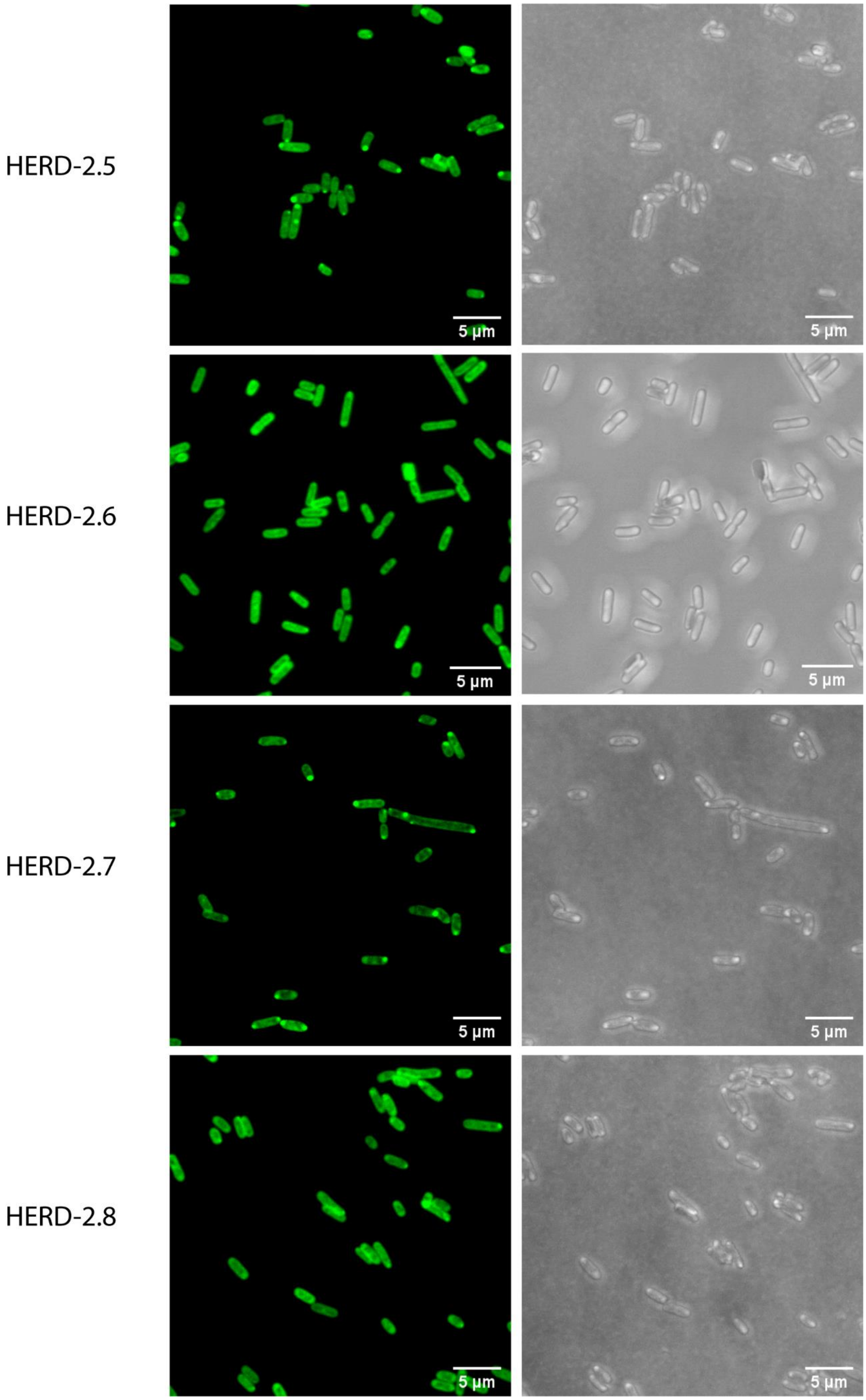

Supplementary Fig. S9: Confocal microscopy images of HERD-2.5 through HERD-2.8 expressed in *E. coli*. mEmerald fluorescence at 488 nm (green) and brightfield transmission (grey).

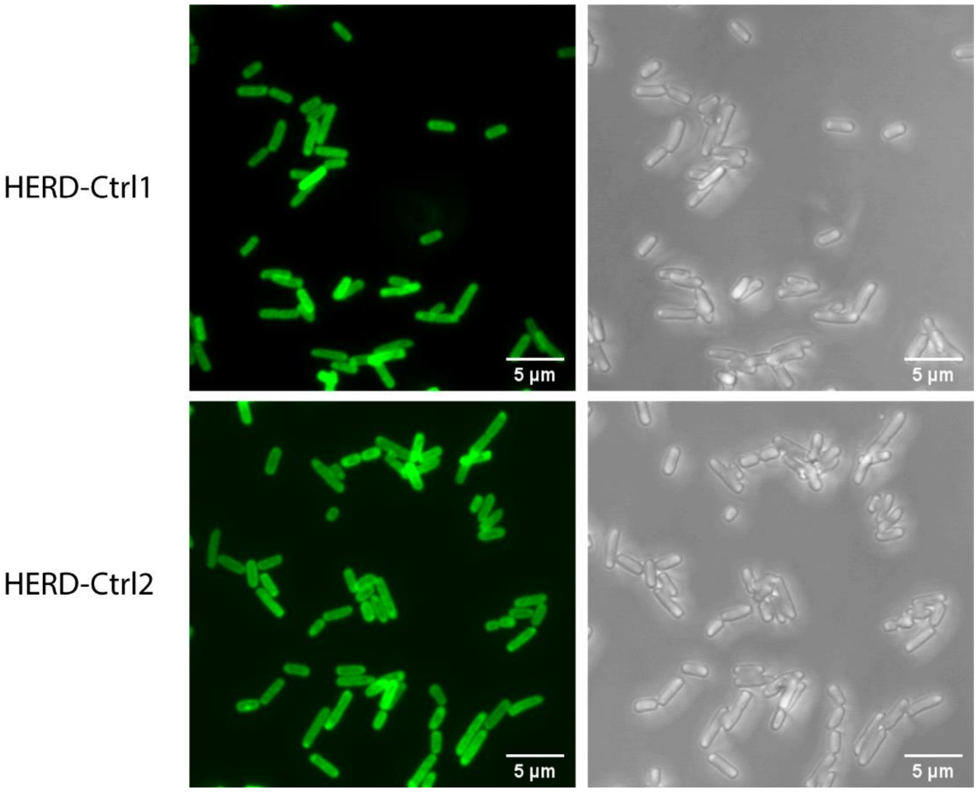

Supplementary Fig. S10: Confocal microscopy images of HERD-Ctrl1 and HERD-Ctrl2 expressed in *E. coli*. mEmerald fluorescence at 488 nm (green) and brightfield transmission (grey).

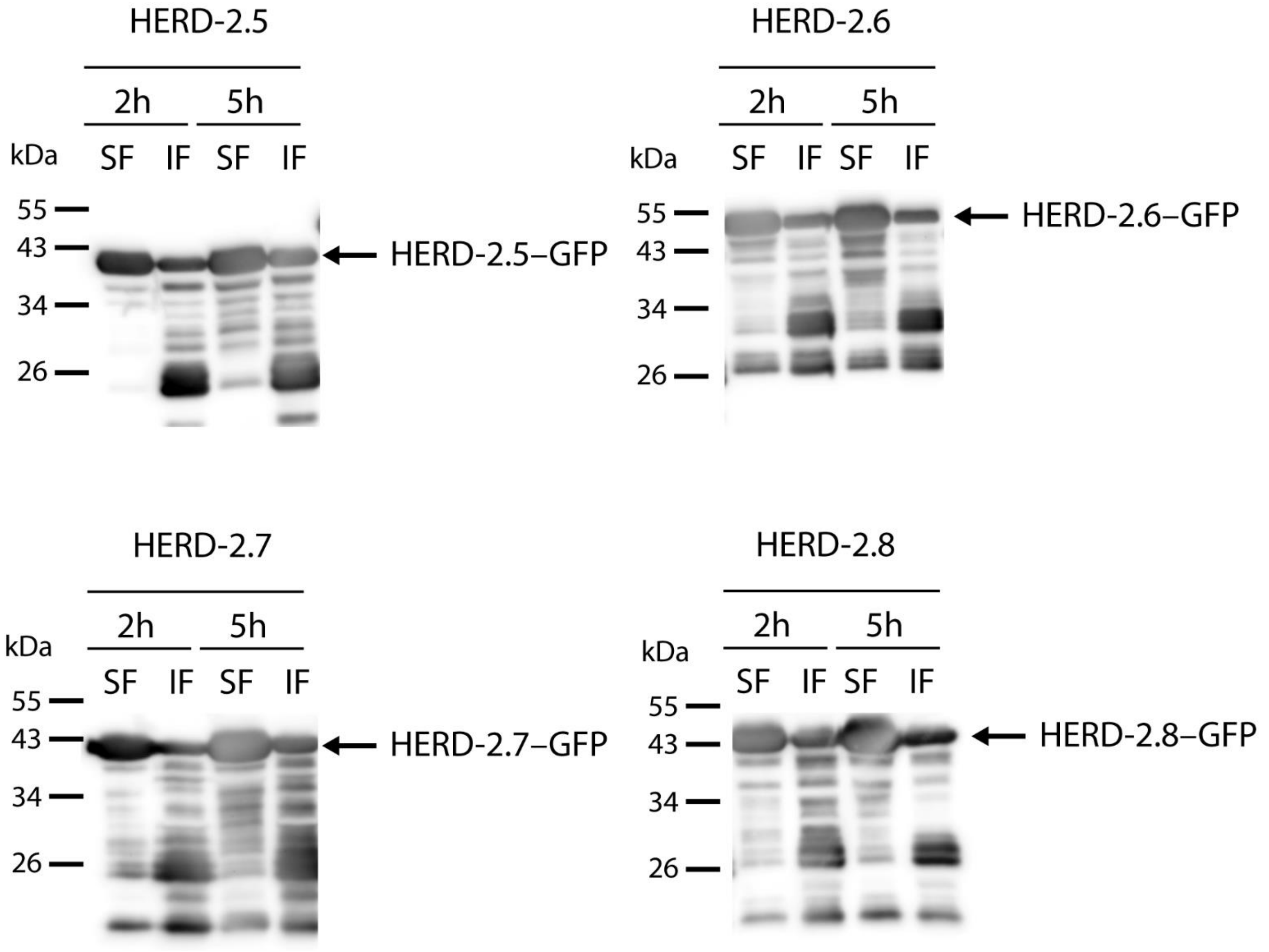

Supplementary Fig. S11: Western blots of HERD-2 .5 through HERD-2.8 fusions from *E. coli* cell lysates. Cell lysates 2 hours (2h) and 5 hours (5h) after induction were separated into their soluble fractions (SF) and insoluble fractions (IF) before analysis by SDS-PAGE and western blotting against the *N*-terminal His epitope tag. Degradation products in the IF lanes, and to a lesser extent in the SF lanes are shown below the bands corresponding to the HERD-GFP fusions.

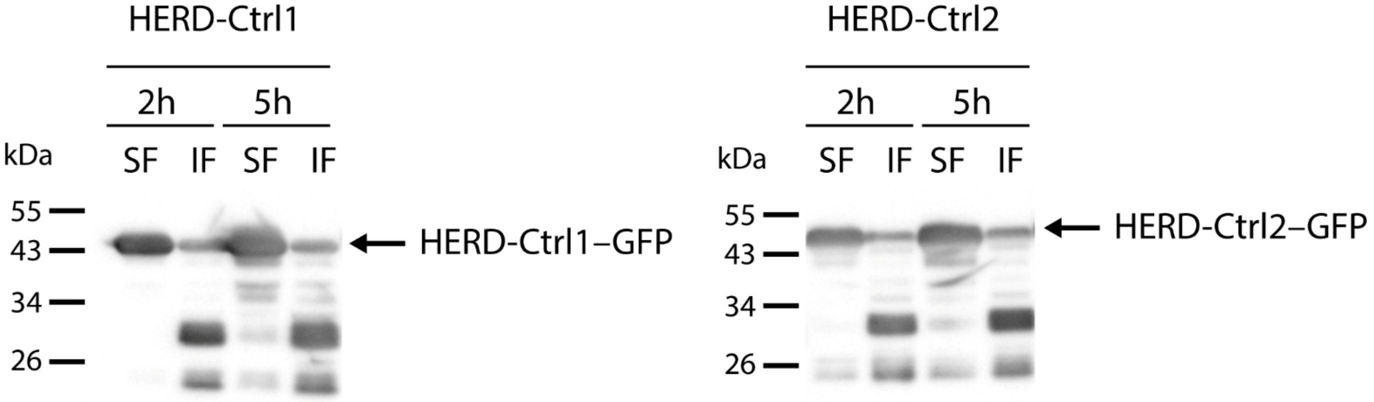

Supplementary Fig. S12: Western blots of HERD-Ctrl1 and HERD-Ctrl2 fusions from *E. coli* cell lysates. Cell lysates 2 hours (2h) and 5 hours (5h) after induction were separated into their soluble fractions (SF) and insoluble fractions (IF) before analysis by SDS-PAGE and western blotting against the *N*-terminal His epitope tag. Degradation products in the IF lanes, and to a lesser extent in the SF lanes are shown below the bands corresponding to the HERD-GFP fusions.

Originally designed linker

ASPEPQPKPS GDPQSKQTPE PSRSQ

Short linker

ASESQKPS

Long linker

ASPEPQPKPS GDPQSKQTPE ASPRPQPEPS
GKPQSEQTPK ASPEPQPKPS

Increased charge density linker

ASEPKQSDPK GDPRSEQKPE KSESR

Uncharged linker

ASPTSYPAPS WAPQYLQTPG PSVSQ

Supplementary Fig. S13: Designed linker variations. Charged residues are shown in blue (positive) and red (negative). Linker variations were designed to vary either the length or the solvation of the linker, and therefore make the helical regions more or less cooperative as stickers. Sequences leading to additional protein-protein interactions were excluded, to maintain the helical regions as the only stickers in the HERD tag. In order to vary the length 2 additional linkers were designed with shorter (8 residues) and longer (50 residues) lengths. These linkers were designed to have similar overall chemistries to the original 25 residue linker (net 0 charge, unstructured, approximately 25% charged residues). To expand on this further, 2 more linkers were created with the original 25 residue length, but with different solvation characteristics. One had a greater proportion of charged residues (50%) but still net 0 charge, and one had all charged residues replaced.

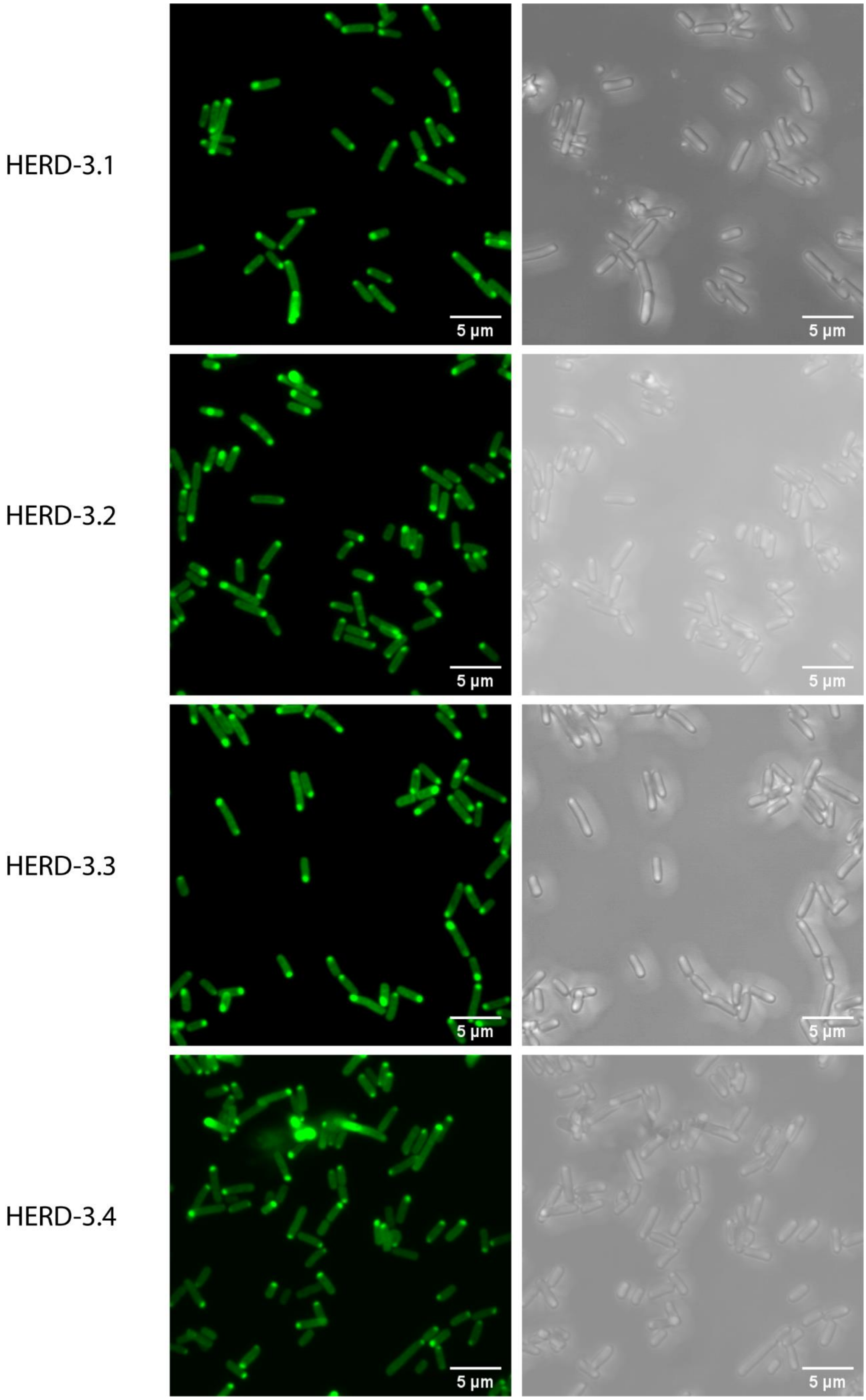

Supplementary Fig. S14: Confocal microscopy images of HERD-3.1 through HERD-3.4 expressed in *E. coli*. mEmerald fluorescence at 488 nm (green) and brightfield transmission (grey).

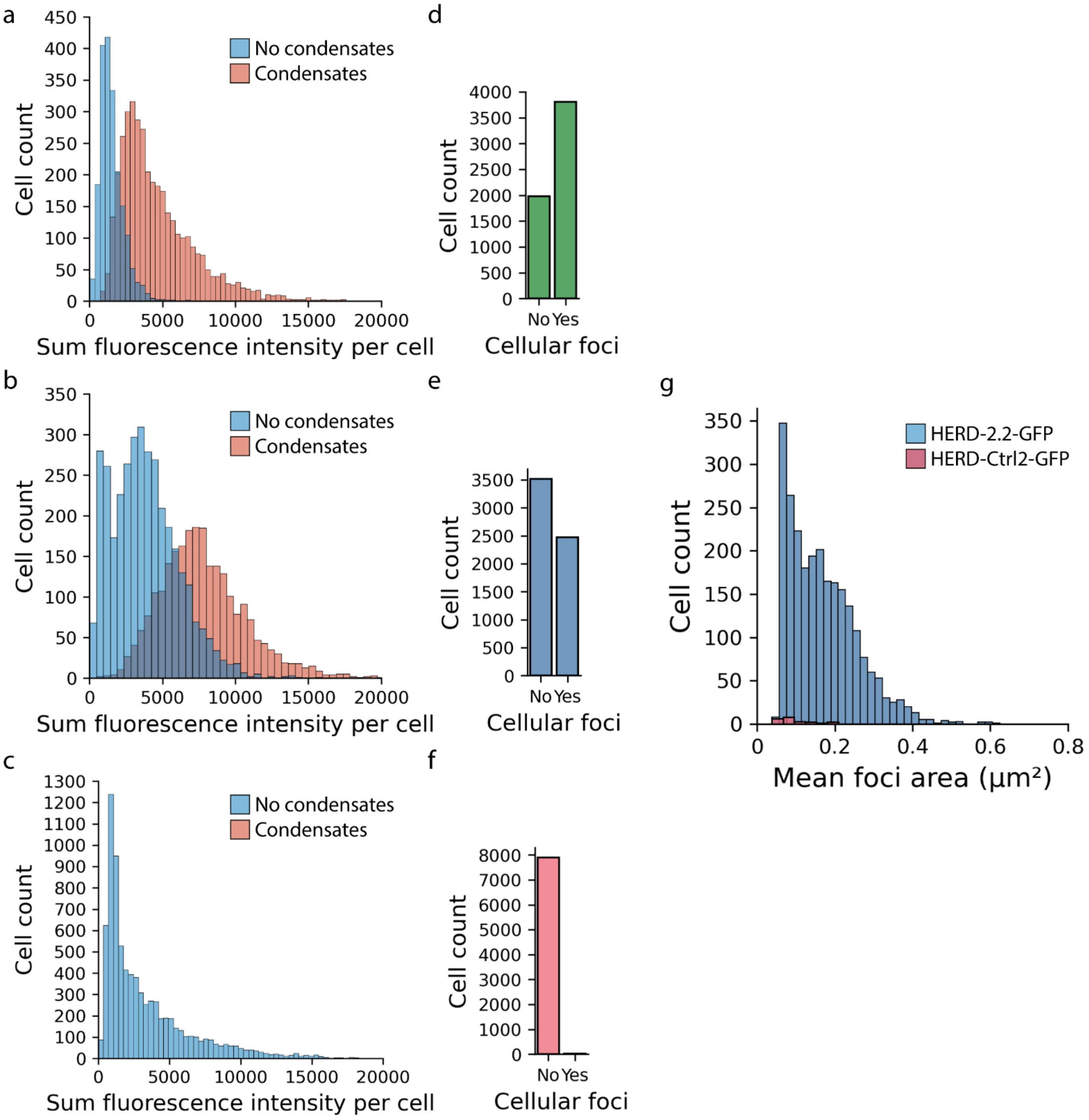

Supplementary Fig. S15: Automated image analysis of HERD–GFP fusions from confocal microscopy. a, b & c, Histogram of cells expressing HERD-0–GFP **(a)**, HERD-2.2–GFP **(b)**, and HERD-Ctrl2–GFP **(c).** Images for analysis taken across 6 hours of protein expression to gather variable intracellular protein concentrations. Cells detected as containing no protein condensates are labelled blue, and cells containing at least one protein condensate are labelled red. **d, e, & f,** Histogram of cells expressing HERD-0–GFP **(d)**, HERD-2.2–GFP **(e)**, and HERD-Ctrl2–GFP **(f)** analysed for the presence of protein condensates. **g,** Histogram of mean protein condensate area in cells expressing HERD-2.2–GFP (blue) and HERD-Ctrl2–GFP (pink).

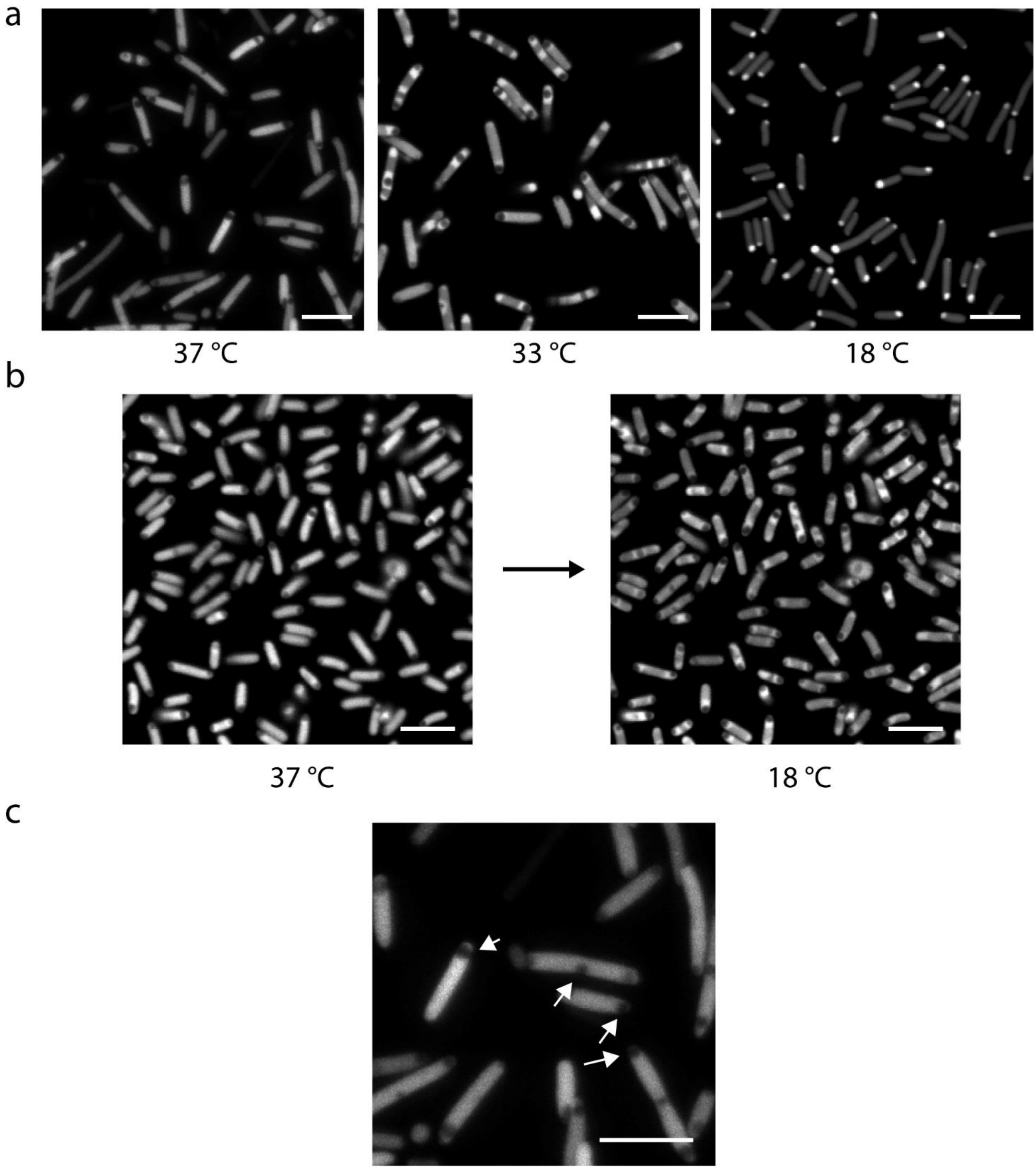

Supplementary Fig. S16: Temperature-controlled live-cell imaging of HERD-2.2–GFP expressed in *E. coli*. a, Images of cells grown and imaged at the indicated temperature. **b,** Images of cells grown at 37 °C and imaged beginning at 37°C and with the temperature gradually lowered on the microscope to 18 °C. **c,** HERD-2.2–GFP grown at 37 °C with non-fluorescent inclusion bodies indicated by white arrows. Non-fluorescent inclusion bodies are a natural part of *E. coli* cell biology, particularly present when over-expressing recombinant proteins. Scale bars 5 μM.

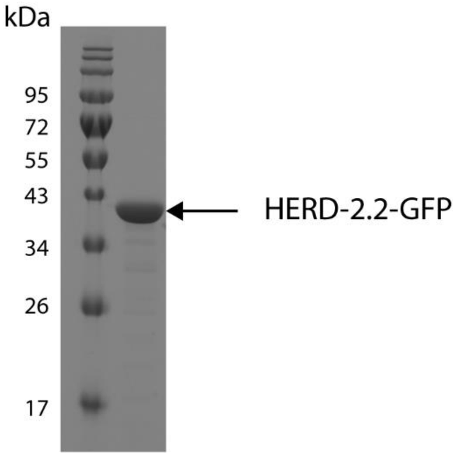

Supplementary Fig. S17: SDS-PAGE gel of purified HERD-2.2–GFP. Molecular weight marker is NEB colour pre-stained protein standard broad range (10 – 250 kDa).

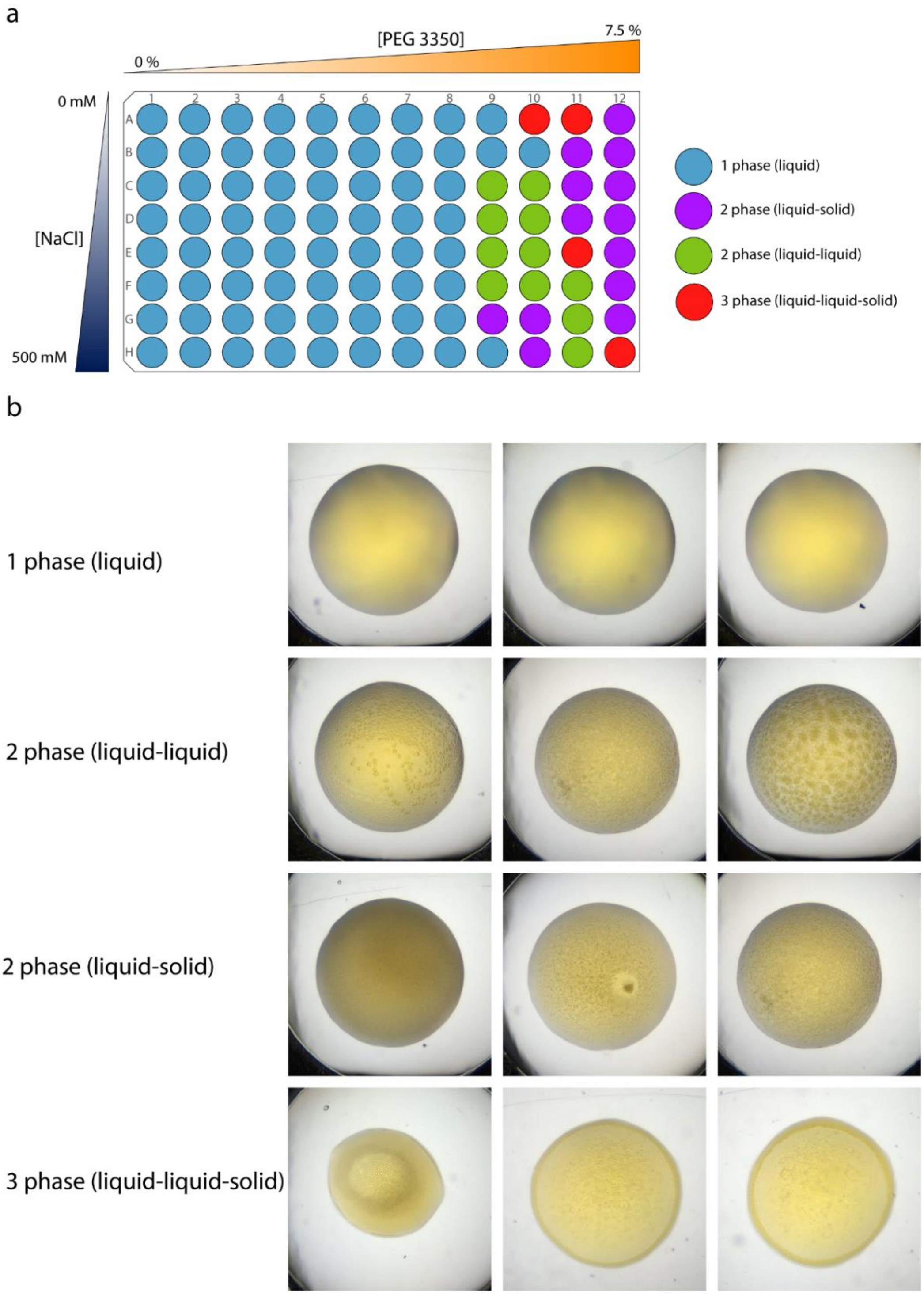

Supplementary Fig. S18: Image based buffer screen for phase separation of HERD-2.2–GFP. a, Tabulated buffer screen for phase separation of HERD-2.2–GFP. A 2 dimensional gradient of NaCl and PEG 3350 was created in a 96 well MRC crystallography plate with HERD-2.2-GFP in a total volume of 0.8 µl, and the droplets automatically imaged in a Formulatrix RI-1000 crystallisation hotel with a 5 MP camera. Conditions – Varying from 0 – 500 mM NaCl, 0 – 7.5% PEG 3350, with 20 mM Tris-HCl pH 7.5, 400 µM HERD-2.2–GFP. **b,** Representative images of the properties described in the tabulated screen for phase separation. Images are of 0.8 µl droplets, approximately 1 – 2 mm in diameter. Classification was made on the following observed properties: 1 phase was classified based on the absence of any protein precipitation or de-mixing. 2 phases (liquid-liquid) was classified based on the appearance of de-mixed particles that appeared largely spherical and had smooth boundaries between the two phases. 2 phases (liquid-solid) was classified based on the appearance off granular particles that were not spherical and had coarse or granular boundaries between the 2 phases, and “darkening” of the solution. 3 phases (liquid-liquid-solid) was classified based on the appearance of both morphologies within the same droplet.

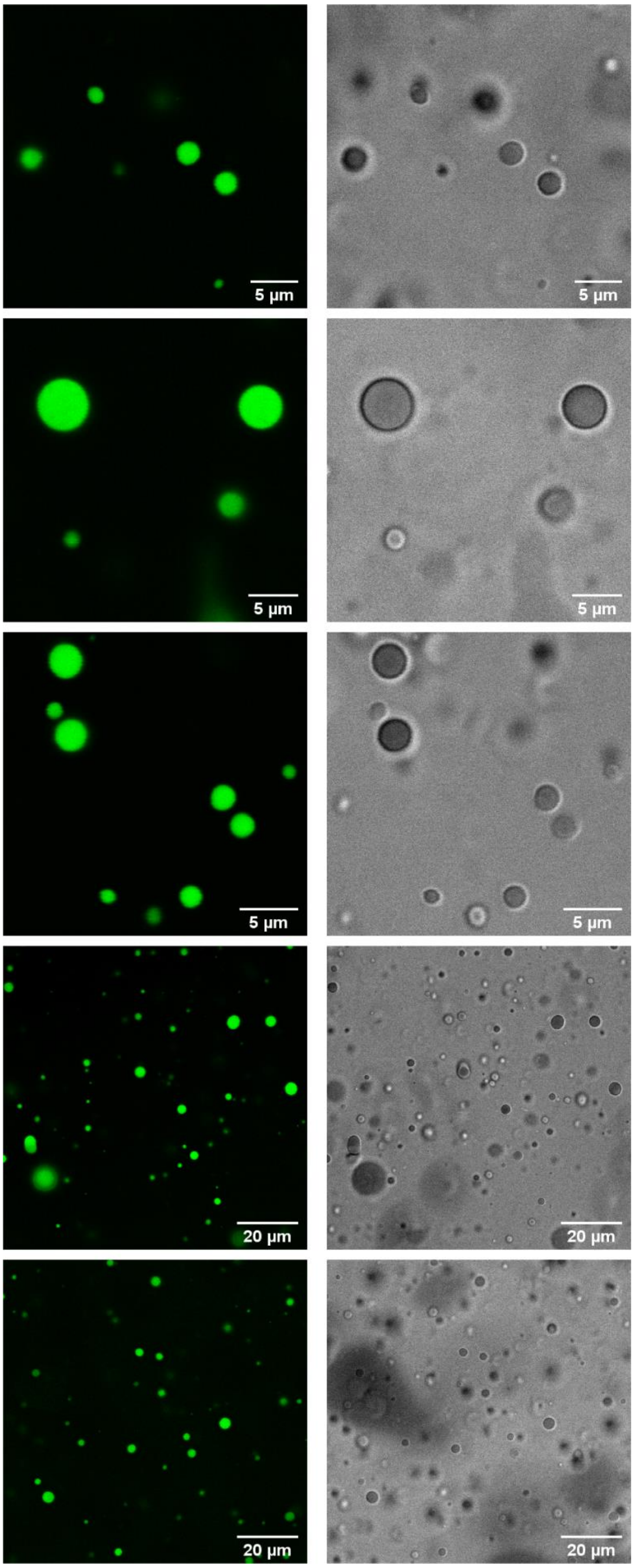

Supplementary Fig. S19: Confocal microscopy images of HERD-2.2-GFP droplets *in vitro*. mEmerald fluorescence at 488 nm (green) and brightfield transmission (grey). Conditions: 125 mM NaCl, 4% PEG 3350, 20 mM Tris-HCl pH 7.5, 1 mM HERD-2.2–GFP. Imaging performed at room temperature (21 °C).

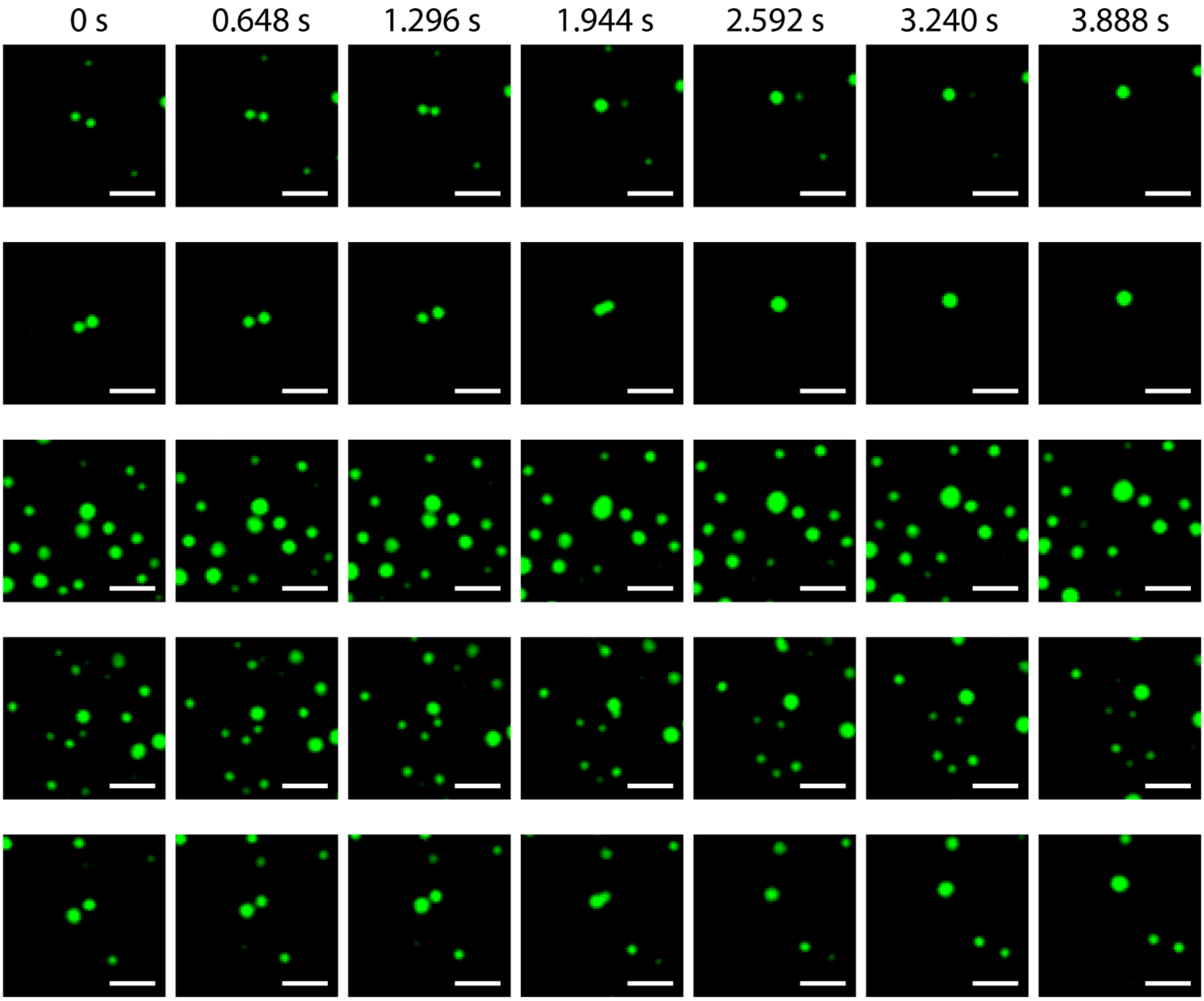

Supplementary Fig. S20: Coalescence of HERD-2.2-GFP droplets. Sequential images taken at 648 ms intervals. Scale bar 10 µM. Conditions: 125 mM NaCl, 10 % PEG 3350, 20 mM Tris-HCl pH 7.5, 1 mM HERD-2.2–GFP.

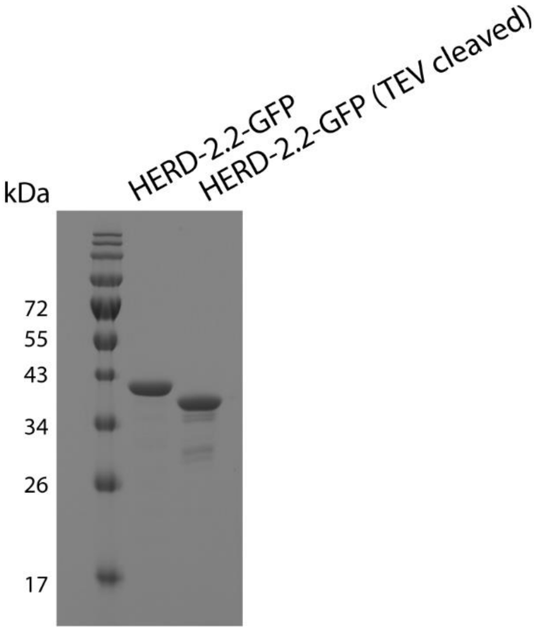

Supplementary Fig. S21: SDS-PAGE of HERD-2.2-GFP before and after TEV cleavage. Molecular weight marker is NEB colour pre-stained protein standard broad range (10 – 250 kDa).

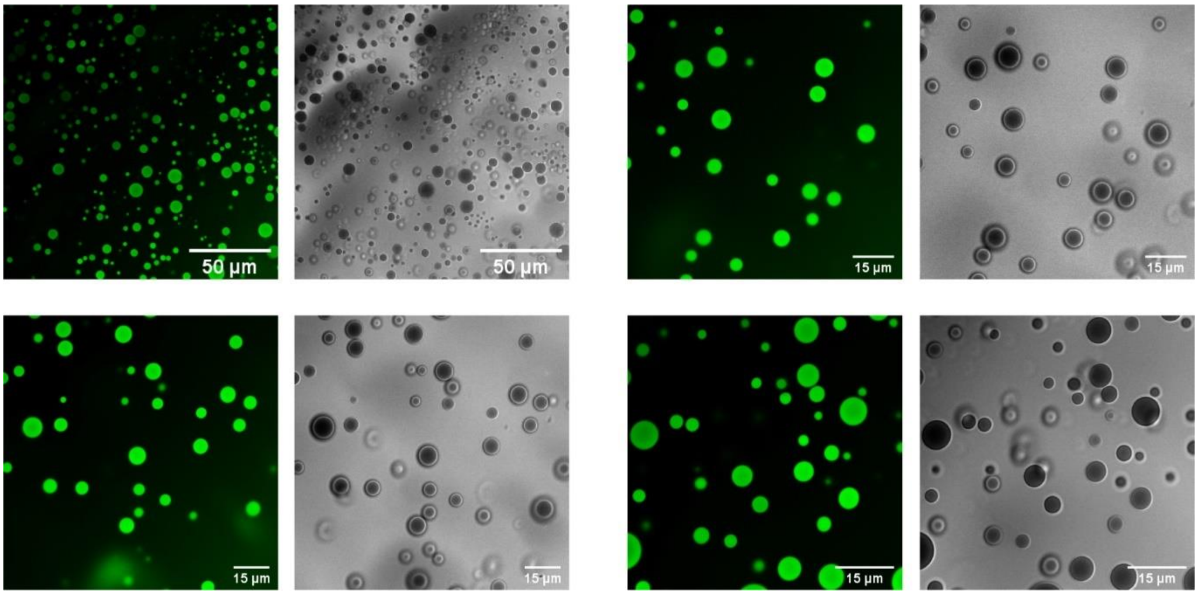

Supplementary Fig. S22: Confocal microscopy of HERD-2.2-GFP droplets *in vitro* after TEV cleavage. mEmerald fluorescence at 488 nm (green) and brightfield transmission (grey). Conditions: 125 mM NaCl, 10 % PEG 3350, 20 mM Tris-HCl pH 7.5, 2 mM HERD-2.2–GFP (cleaved).

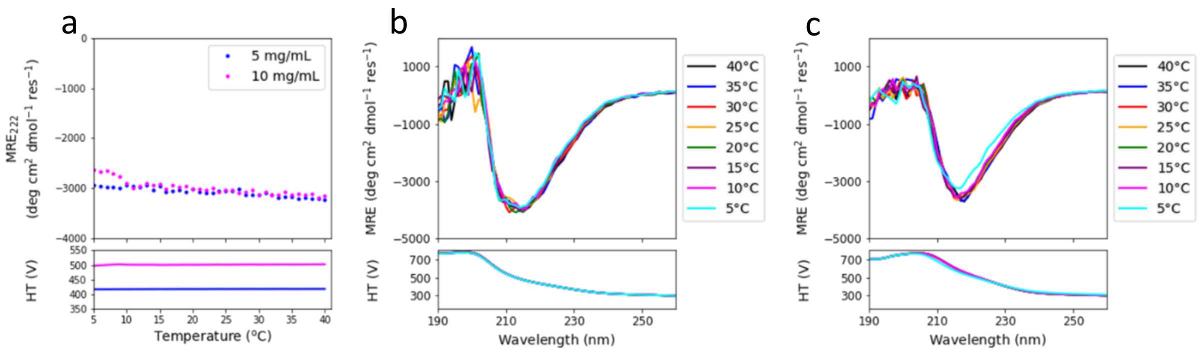

Supplementary Fig. S23: Circular dichroism spectroscopy of HERD-2.2-GFP. a, Changes in MRE_{222} at 5 and 10 mg/ml protein concentration. **b & c,** CD spectra for 5 and 10 mg/ml protein corresponding to the curves on panel **a**. Spectra were recorded from 40 – 5 °C with a 5 °C step. Conditions: 20 mM Tris-HCl pH 7.5, 125 mM NaCl, 4% PEG3350, 0.1 mm path length.

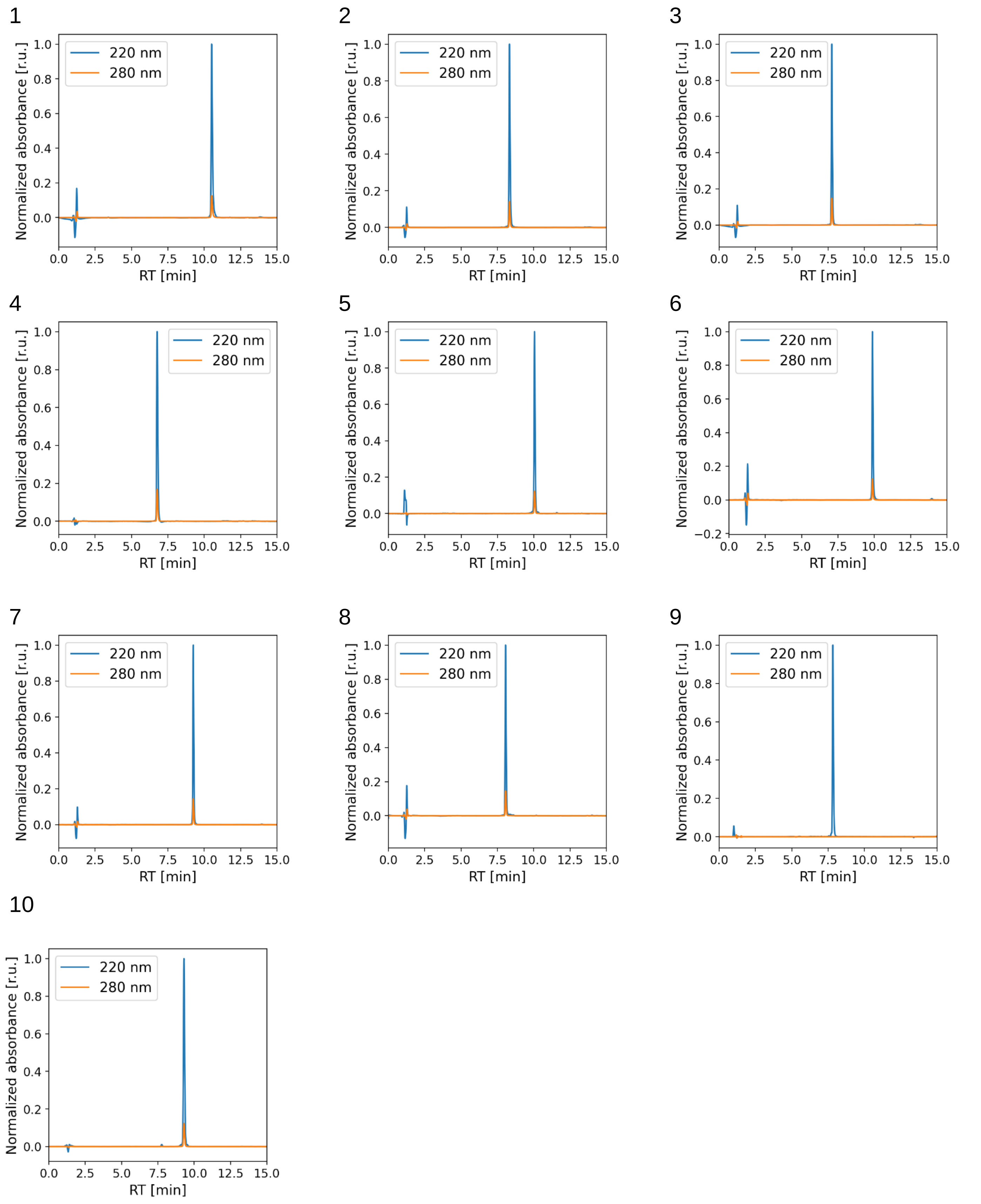

Supplementary Fig. S24: Analytical HPLC traces of synthesised peptides 1-10. Absorbances monitored at 220 and 280 nm are normalised intensities to the maxima at 220 nm. Peptides are numbered as in Supplementary Table S3. HPLC was performed using a C18 reverse phase column (Phenomenex Kinetex C18, 5 µm particle size, 100 Å pore size, 100 × 4.6 mm), a gradient of Buffer A (0.1 % TFA in H_2O) and Buffer B (0.1 % TFA in MeCN), at a flow rate of between 1-3 ml/min.

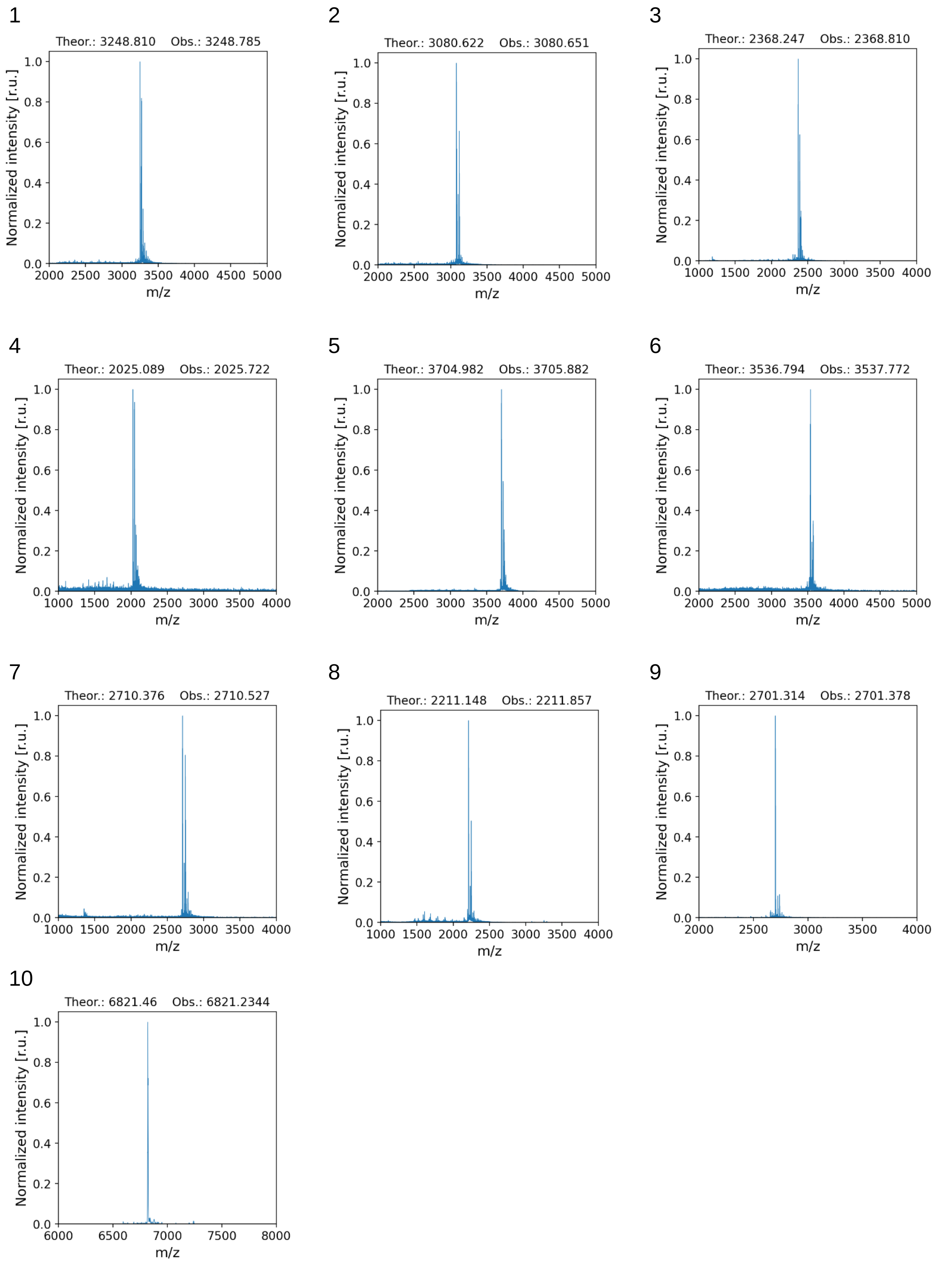

Supplementary Fig. S25. MALDI-TOF MS spectra for peptides 1-9 and ESI MS spectrum for peptide 10. Mass spectra of the synthetic peptides given in Supplementary Table S3. ESI MS spectrum has been deconvoluted.

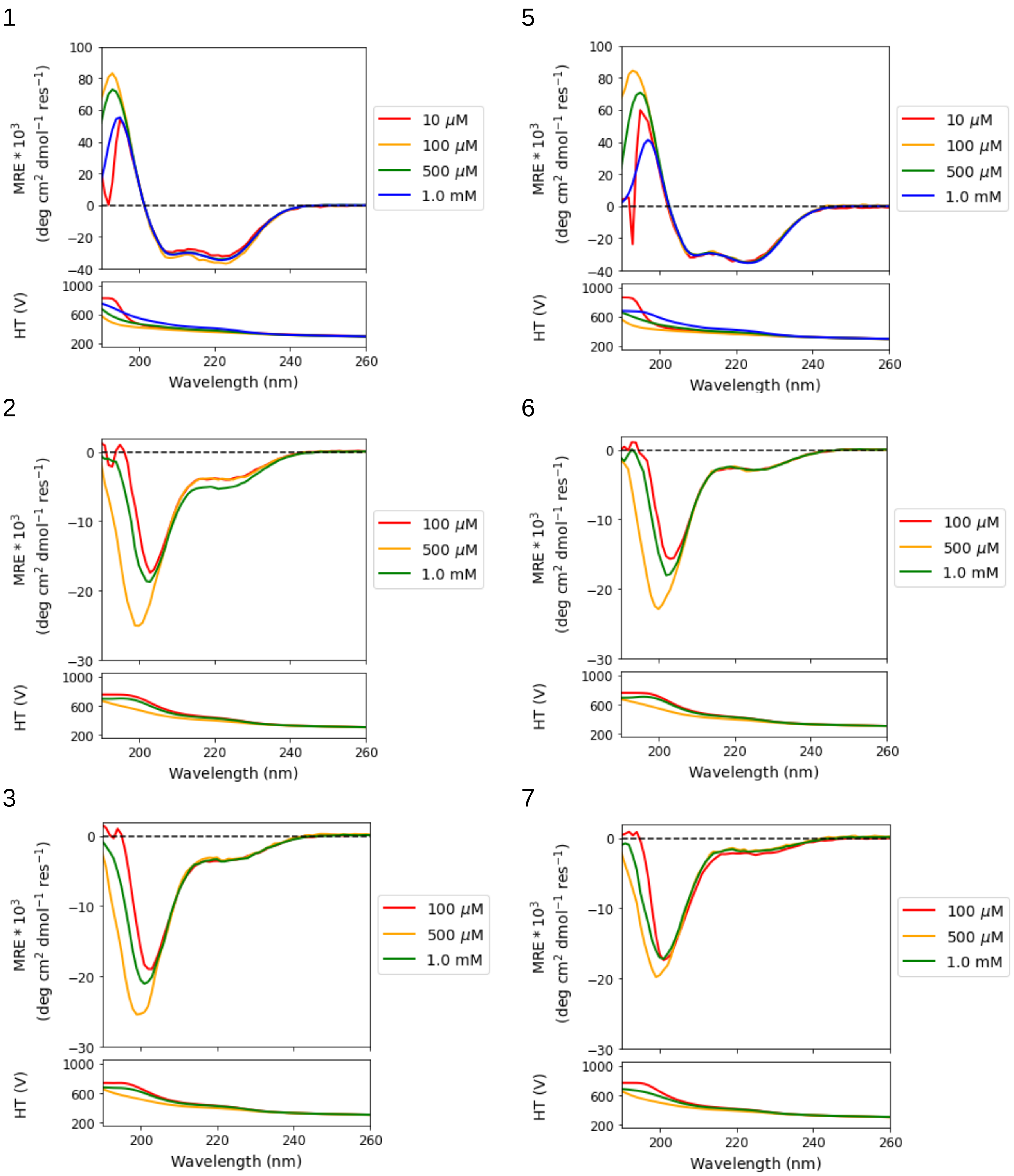
1
5
2
6
3
7
MRE * 10^3 (deg cm^2 dmol^{-1} res^{-1})
HT (V)
Wavelength (nm)
10 μM
100 μM
500 μM
1.0 mM

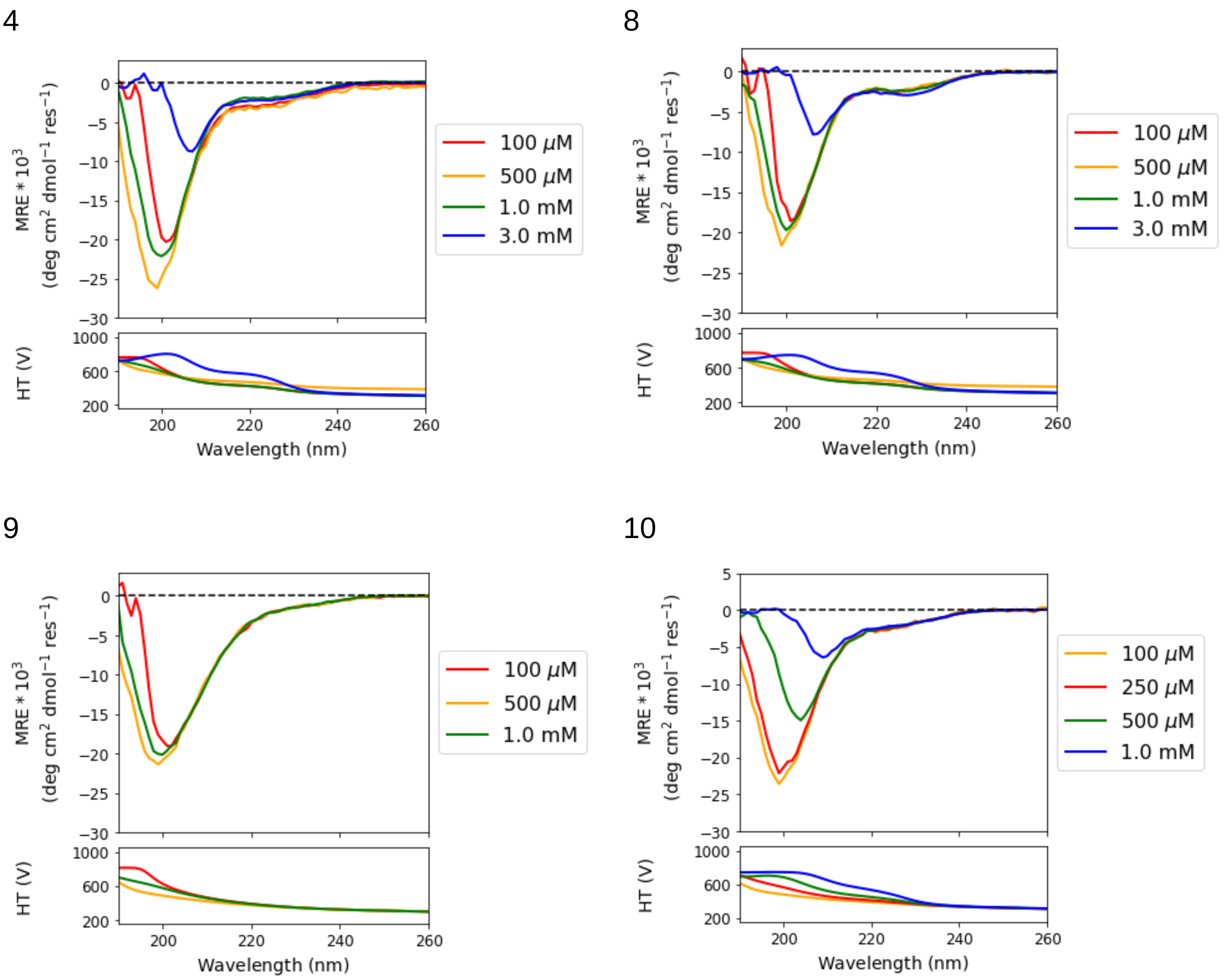

Supplementary Fig. S26. Circular dichroism spectra for the chemically synthesised peptides 1 – 10. Peptide sequences are given in Supplementary Table S3. Conditions: 20 mM Tris-HCl pH 7.5, 125 mM NaCl, 5 °C, with varied peptide concentrations.

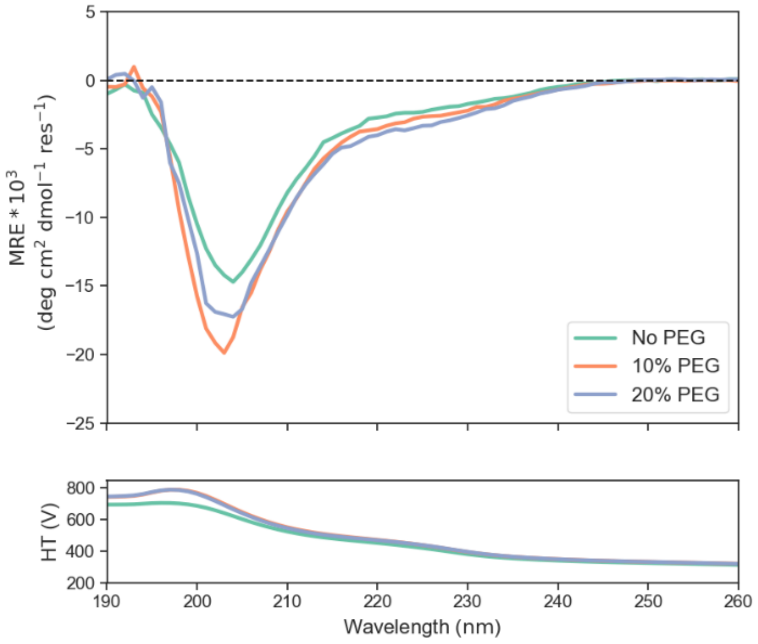

Supplementary Fig. S27: CD spectra for peptide 10 at different PEG 3350 concentrations. Conditions: 500 µM peptide 20 mM Tris-HCl pH 7.5, 125 mM NaCl, 5 °C.

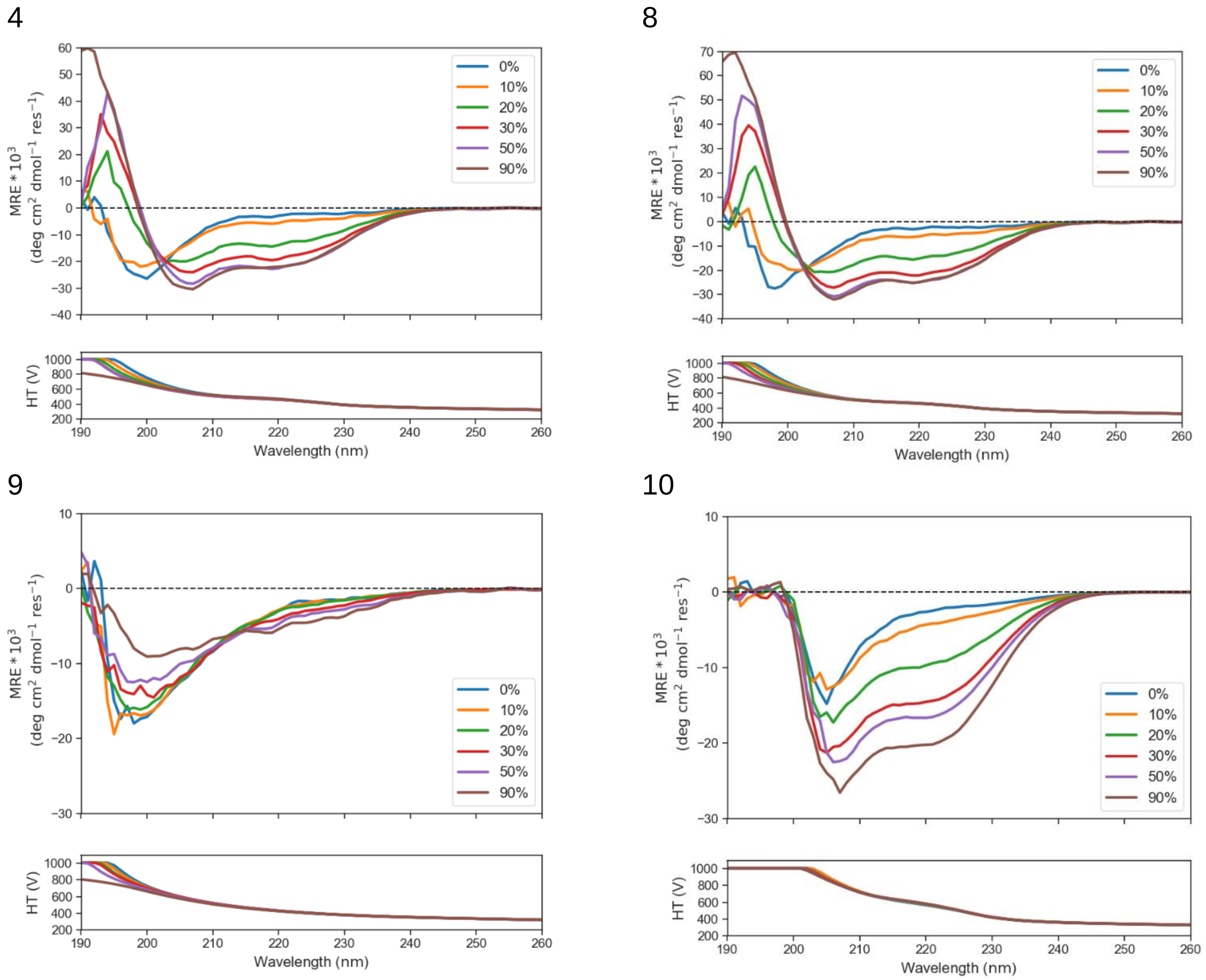

Supplementary Fig. S28: CD spectra for the peptides 4 and 8 – 10 at 100 µM in TFE (0 – 90%). Peptide sequences are given in Supplementary Table S3. Conditions: 20 mM Tris-HCl pH 7.5, 125 mM NaCl, 5 °C.

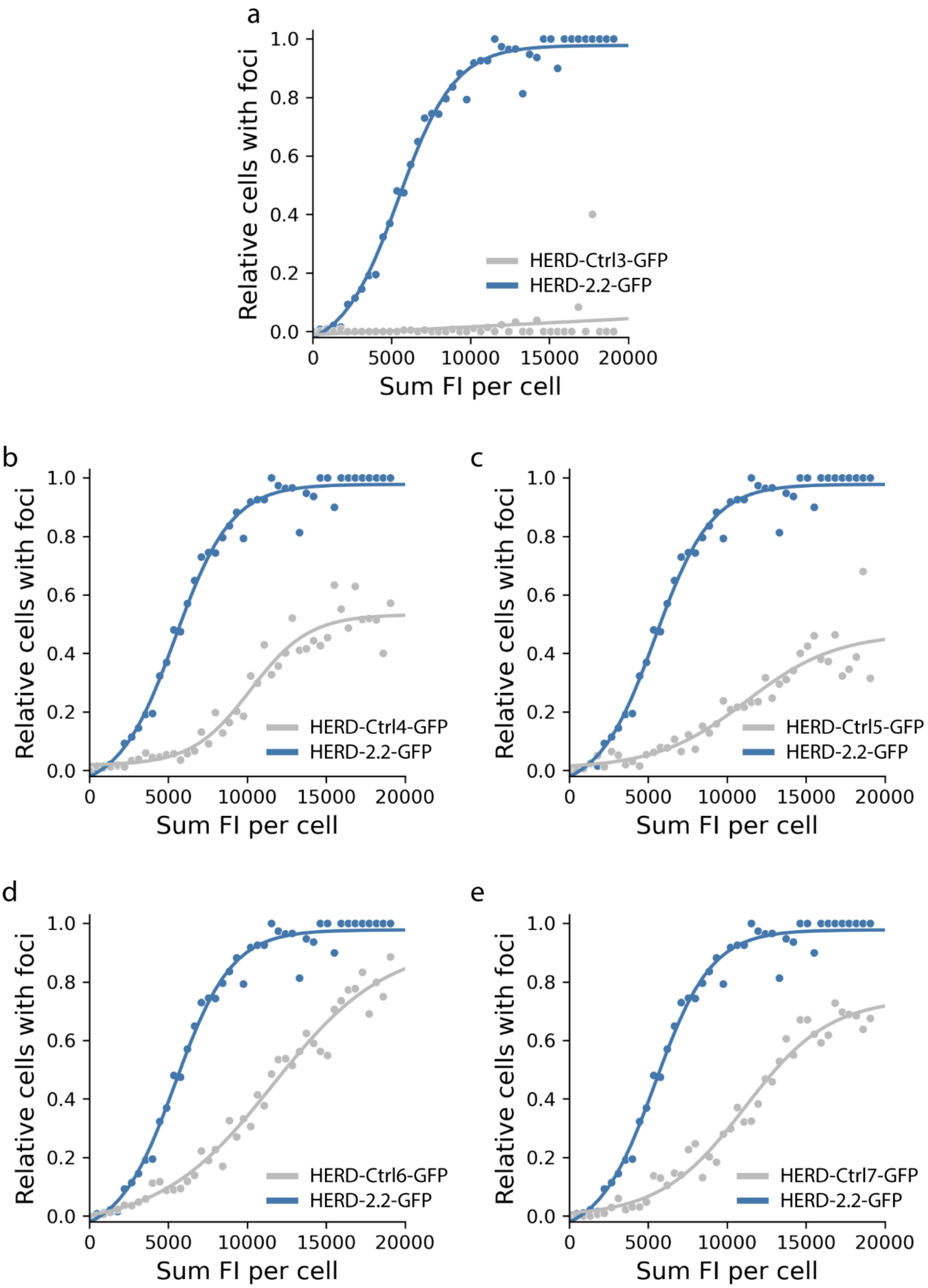

Supplementary Fig. S29: Automated image analysis of helix destabilising HERD-GFP constructs. Comparison of protein condensate formation between cells expressing HERD-2.2–GFP (blue, n = 5993) and cells expressing constructs with the destabilising mutations (grey). Helix disrupted HERD fusions in each panel: **a**, HERD-Ctrl3–GFP, n = 4141; **b**, HERD-Ctrl4–GFP, n = 5530; **c**, HERD-Ctrl5–GFP, n = 5554; **d**, HERD-Ctrl6–GFP, n = 4339; **e** – HERD-Ctrl7–GFP, n = 4113.

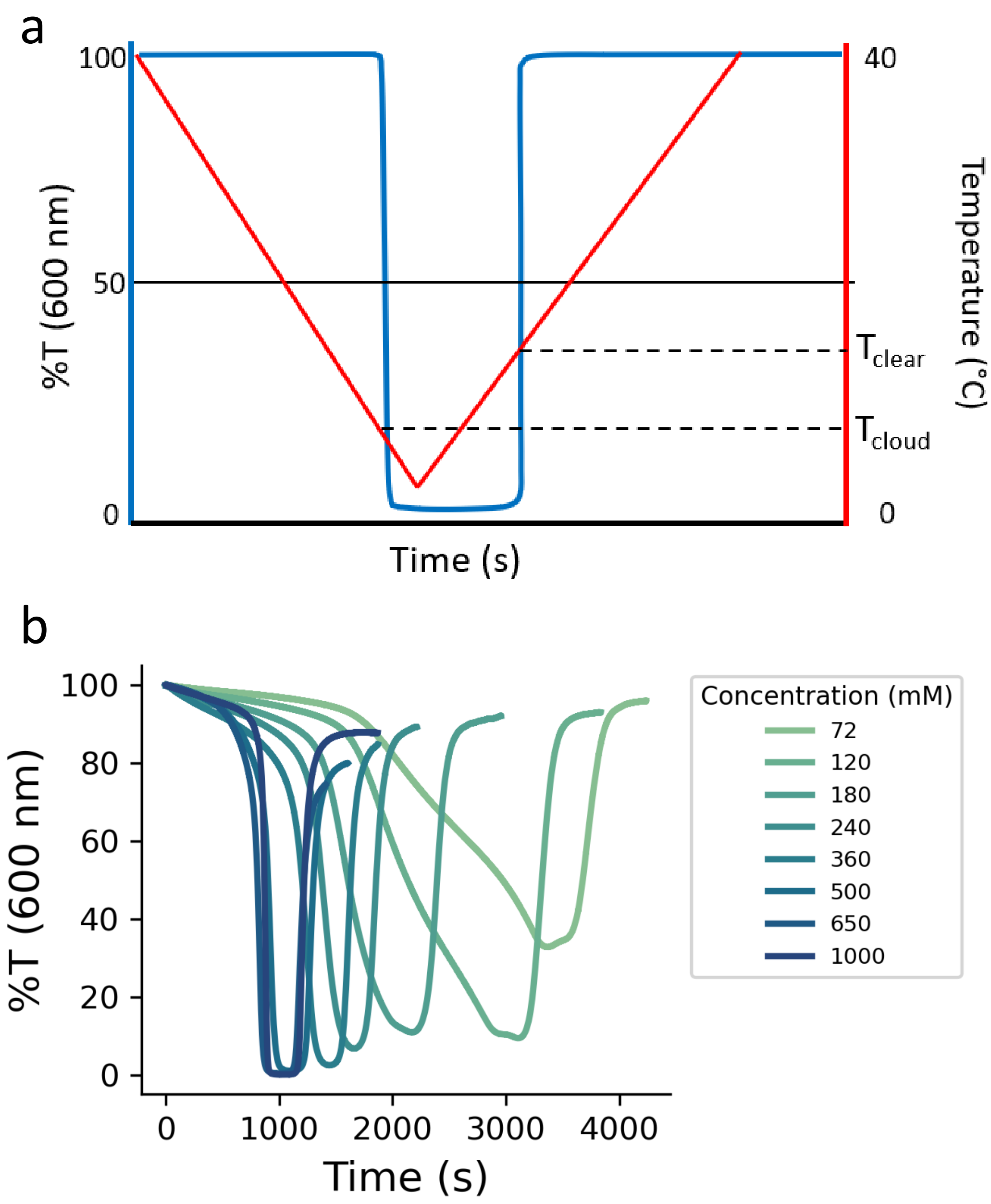

Supplementary Fig. S30: Cloud-point measurements for HERD-2.2–GFP *in vitro* with respect to temperature. a, Graphical representation of cloud-point measurements, showing the decrease in percent transmission (blue; %T, 600 nm) as the temperature is reduced (red; °C), followed by a subsequent recovery in %T as the temperature is increased. The points T_{cloud} and T_{clear} are the 50% transmission points as the temperature is reduced and increased respectively, and show a characteristic hysteresis associated with nucleation. **b**, Cloud point measurements of HERD-2.2–GFP varied from 72 μM to 1 mM. Conditions: 20 mM Tris-HCl pH 7.5, 125 mM NaCl , 4% PEG 3350.

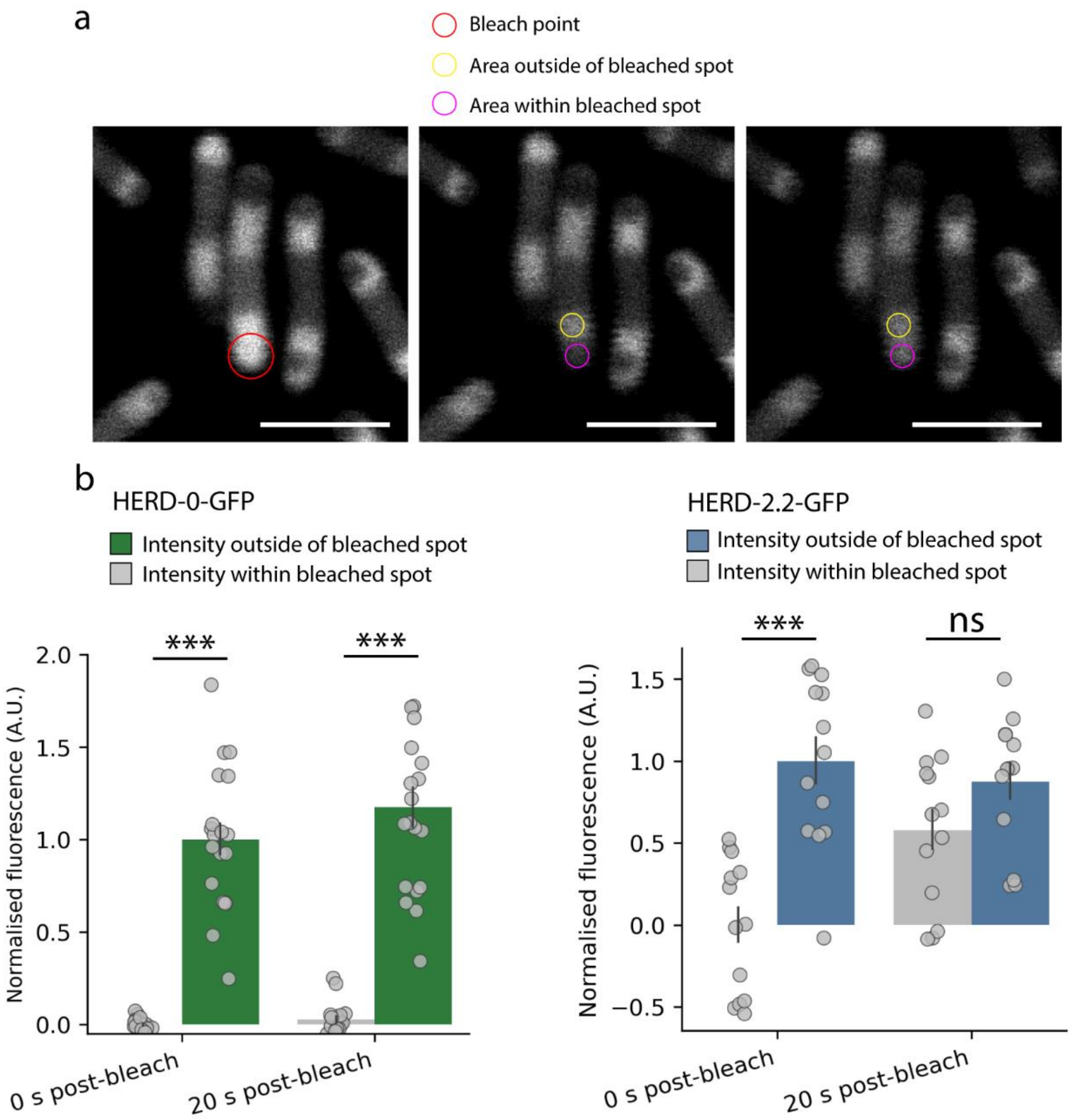

Supplementary Fig. S31: Asymmetrical bleaching of HERD-0–GFP and HERD-2.2–GFP condensates in cells*.* a, Illustration of condensate bleaching in cells, showing the bleach point (red) and the two measurement areas: directly within the bleached area (purple) and outside of the bleached area (yellow). Scale bars, 5 µm. **b,** Normalised fluorescence intensity within the directly bleached area is shown in grey, fluorescence intensity outside of the bleached area within the same droplet is shown in green (HERD-0–GFP; n = 19) and blue (HERD-2.2–GFP; n = 13). Fluorescence intensity was normalised relative to the mean fluorescence intensity in the bleached area (0) and the mean fluorescence intensity outside of the bleached area, in the same foci (1). Data are represented as the mean +/- the standard error from biologically independent cells. Statistical testing used a two tailed t-test comparing the normalised fluorescence intensity within (in-spot) the bleached spot and outside (ex-spot) of the bleached spot. Samples were confirmed to conform to assumptions for equal variance and normality by Shapiro testing and Fligner-Killeen testing respectively. P-value = 0.001 for all comparisons aside from HERD-2.2–GFP 20 s post-bleach (P-value = 0.0958). Mean and standard deviation for all samples as follows. HERD-0–GFP: 0 s post bleach; in-spot mean = 0.000, standard deviation = 0.031; ex-spot mean = 1.000, standard deviation = 0.374. 20 s post bleach; in-spot mean = 0.027, standard deviation = 0.080; ex-spot mean = 1.17, standard deviation = 0.471. HERD-2.2–GFP: 0 s post bleach; in-spot mean = 0.000, standard deviation = 0.395, ex-spot: mean = 1.000, standard deviation = 0.492. 20 s post bleach; in-spot mean = 0.579, standard deviation = 0.444; ex-spot mean = 0.875, standard deviation = 0.391.

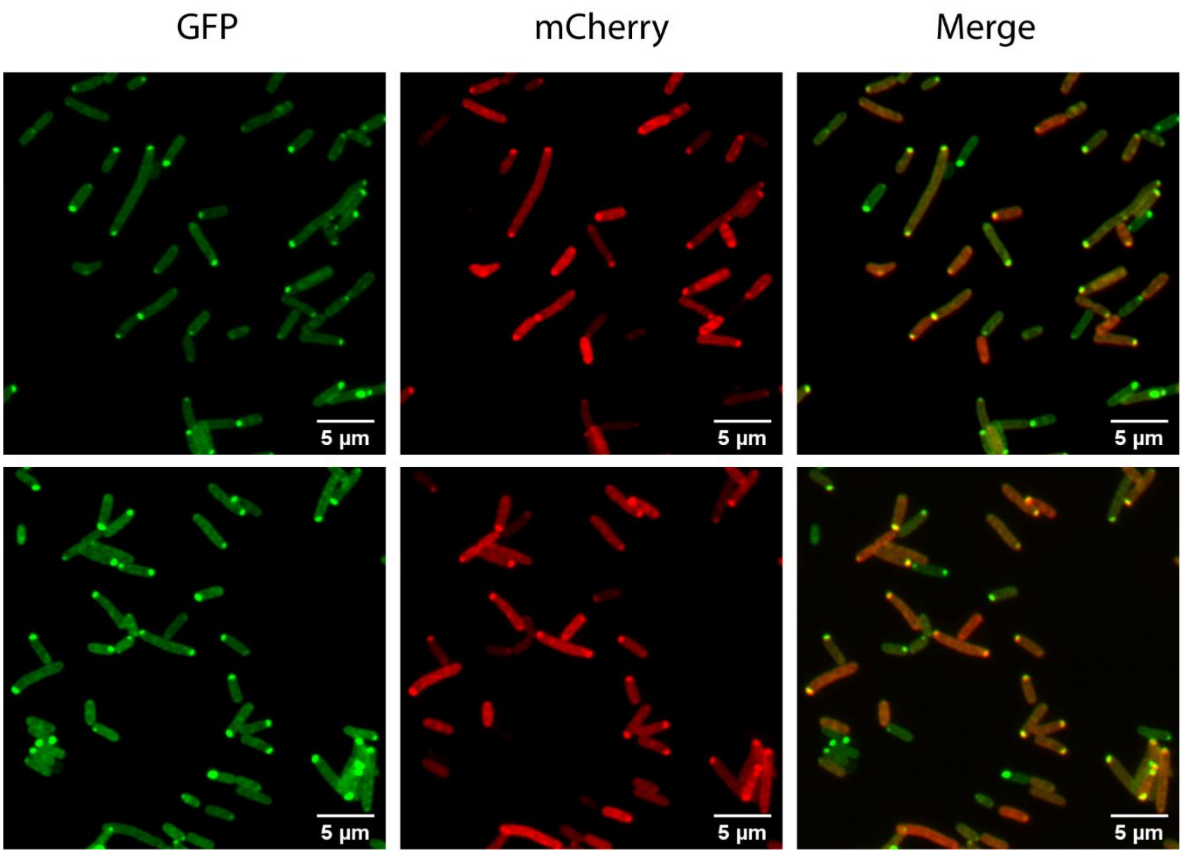

Supplementary Fig. S32: Confocal microscopy images of HERD-2.2–GFP co-expressed with HERD-2.2–mCherry. mEmerald fluorescence at 488 nm (green), mCherry fluorescence at 561 nm (red), and the merged channels.

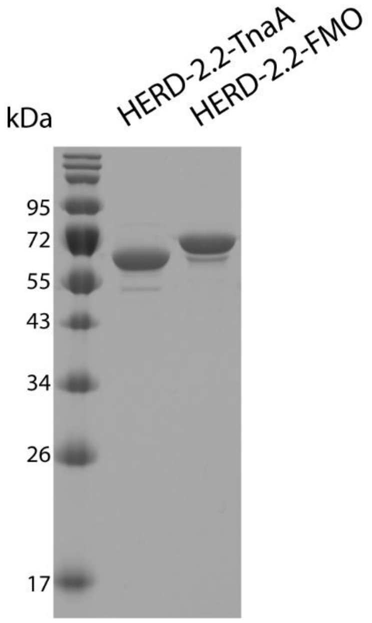

Supplementary Fig. S33: SDS-PAGE of HERD-2.2 fusions with TnaA and FMO. Molecular weight marker is NEB colour pre-stained protein standard broad range (10 – 250 kDa).

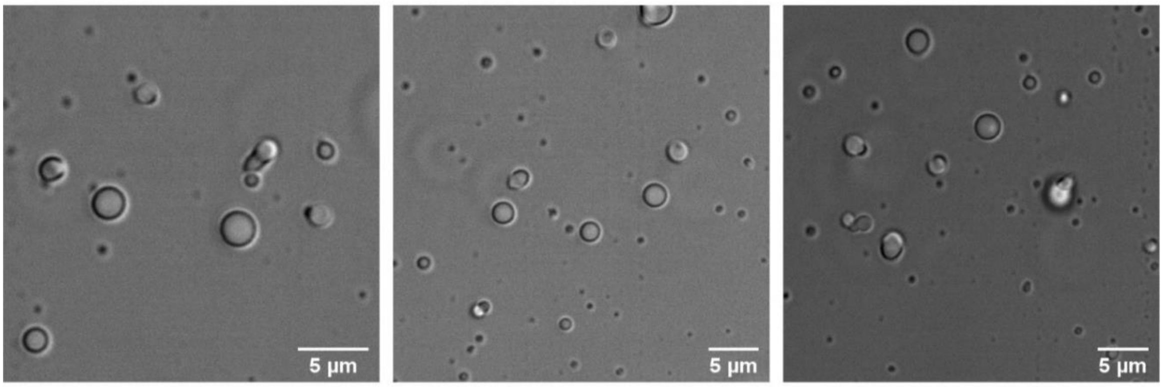

Supplementary Fig. S34: Brightfield transmission images of de-mixed droplets formed by HERD-2.2–TnaA. Conditions: 200 mM NaCl, 8% PEG 3350, 40 mM Bis-Tris-HCl pH 6, 120 µM HERD-2.2–TnaA.

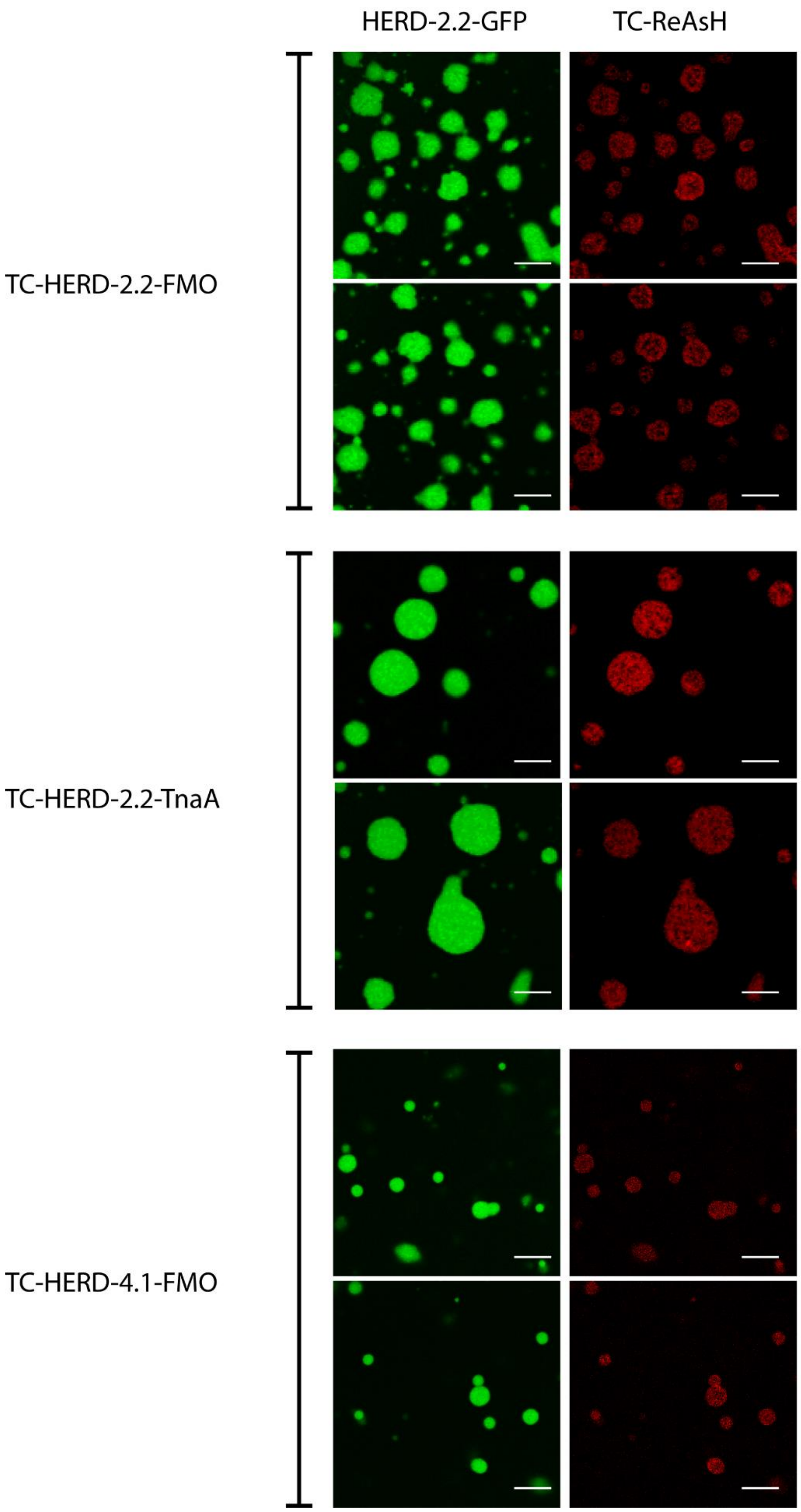

Supplementary Fig. S35: In vitro confocal microscopy images of enriched TC tagged HERD-2.2 fusions. mEmerald fluorescence at 488 nm corresponding to HERD-2.2–GFP (green), and TC-ReAsH fluorescence at 561 nm corresponding to the enriched tetra-cysteine tagged enzyme fusions (red). Conditions: 250 nM TC-ReAsH, 125 mM NaCl, 4% PEG 3350, 20 mM Tris-HCl pH 7.5, 500 μM HERD-2.2–GFP, 25 μM TC-HERD-2.2–TnaA/TC-HERD-2.2–FMO/TC-HERD-4.1–FMO. Scale bar 10 μM.

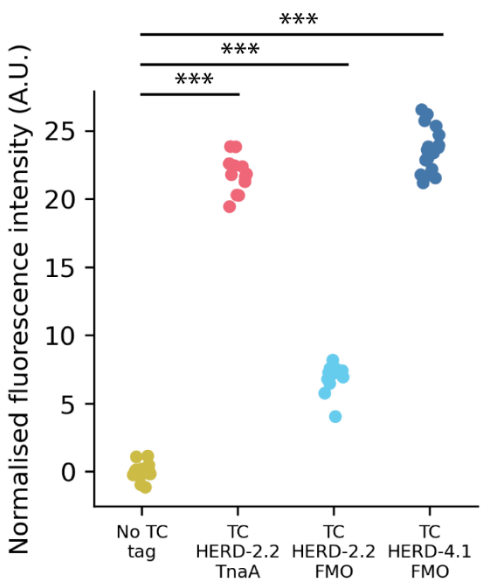

Supplementary Fig. S36: Quantification of enriched TC tagged HERD-2.2 fusions. Quantification of the fluorescence intensity by excitation at 561 nm (for TC-ReAsH) in de-mixed droplets formed by HERD-2.2-GFP shown as scatter plot chart (left) and bar plot (right). Samples are discrete droplets measured independently. Fluorescence intensity was normalised by subtraction of the fluorescence intensity within HERD-2.2-GFP droplets containing ReAsH dye but no TC tagged proteins. Statistical analysis was performed by one way ANOVA between the 4 samples followed by TukeyHSD post-hoc test to perform a multiple comparison of means and variance. P = 0.001 (***) for all comparisons. Sample size, mean and standard deviation for each sample: No TC tag: n = 23, mean = 0.00, standard deviation = 0.48; TC-HERD-2.2-TnaA: n = 12, mean = 21.79, standard deviation = 1.30; TC-HERD-2.2-FMO: n = 12, mean = 6.84, standard deviation = 1.03; TC-HERD-4.1-FMO: n = 18, mean = 23.56, standard deviation = 1.58.

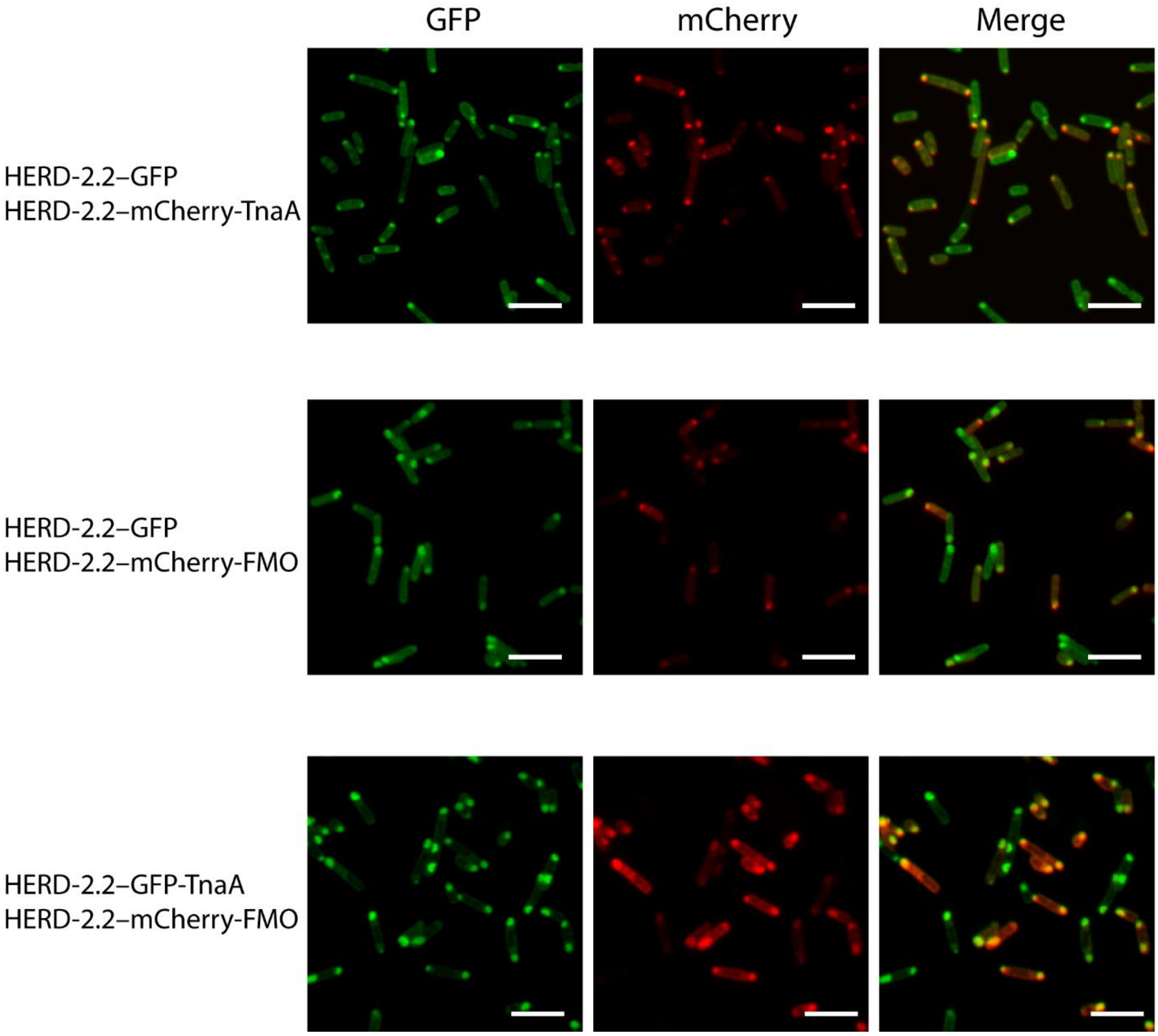

Supplementary Fig. S37: Confocal microscopy images of TnaA and FMO HERD constructs co-expressed in *E. coli*. mEmerald fluorescence at 488 nm (green), mCherry fluorescence at 561 nm (red), and the merged channels. Scale bar 5 μm.

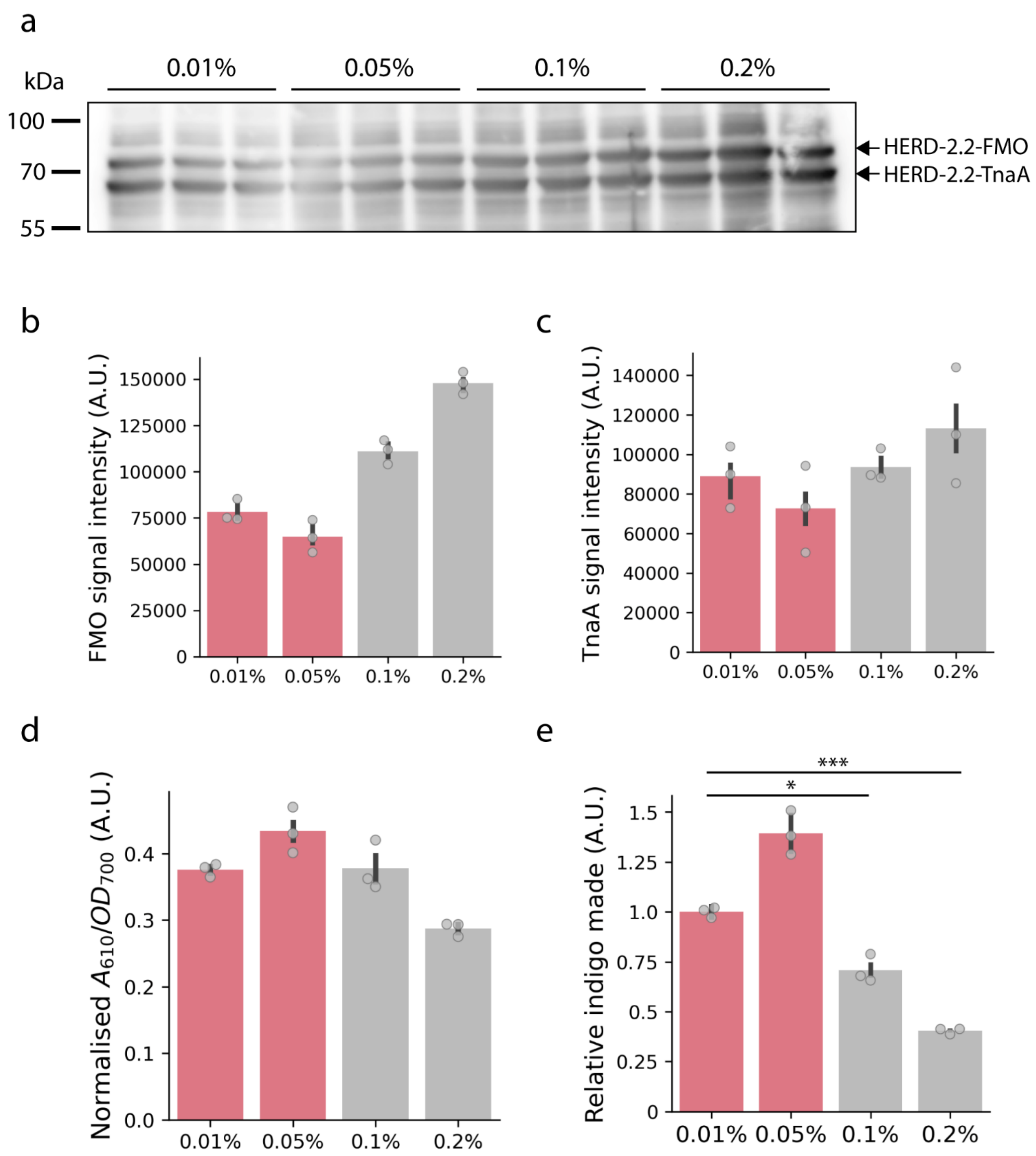

Supplementary Fig. S38: Quantification of enzyme expression and indigo production of HERD-2.2. a, Western blot for HERD-2.2-FMO and HERD-2.2-TnaA (transferred from a 10% acrylamide/bis-acrylamide SDS-PAGE gel). **b, c,** Quantification of HERD-2.2-FMO and HERD-2.2-TnaA expression from western blotting. Quantification performed using Image Studio Lite with upper and lower median background subtraction to normalise for background signal. Data are represented as the mean +/- the standard error from n = 3 biologically independent experiments for each sample. **d,** Quantification of indigo produced (A_{610}), normalised by cell density (OD_{700}) to give the raw amount of indigo produced per culture. Data are represented as the mean +/- the standard error from n = 3 biologically independent experiments for each sample. **e,** Amount of indigo produced normalised both by cell density and by FMO expression levels to give the relative amount of indigo made. Data are represented as the mean +/- the standard error from n = 3 biologically independent experiments for each sample. Mean and standard deviation for each sample: 0.01%: mean = 1.00, standard deviation = 0.07; 0.05%: mean = 1.39, standard deviation = 0.18; 0.1%: mean = 0.71, standard deviation = 0.07; 0.2%: mean = 0.41, standard deviation = 0.02. 0.01% vs 0.1% P = 0.0318 (*); 0.01% vs 0.2% P = 0.001 (***) by one-way ANOVA and Tukey HSD post-hoc test.

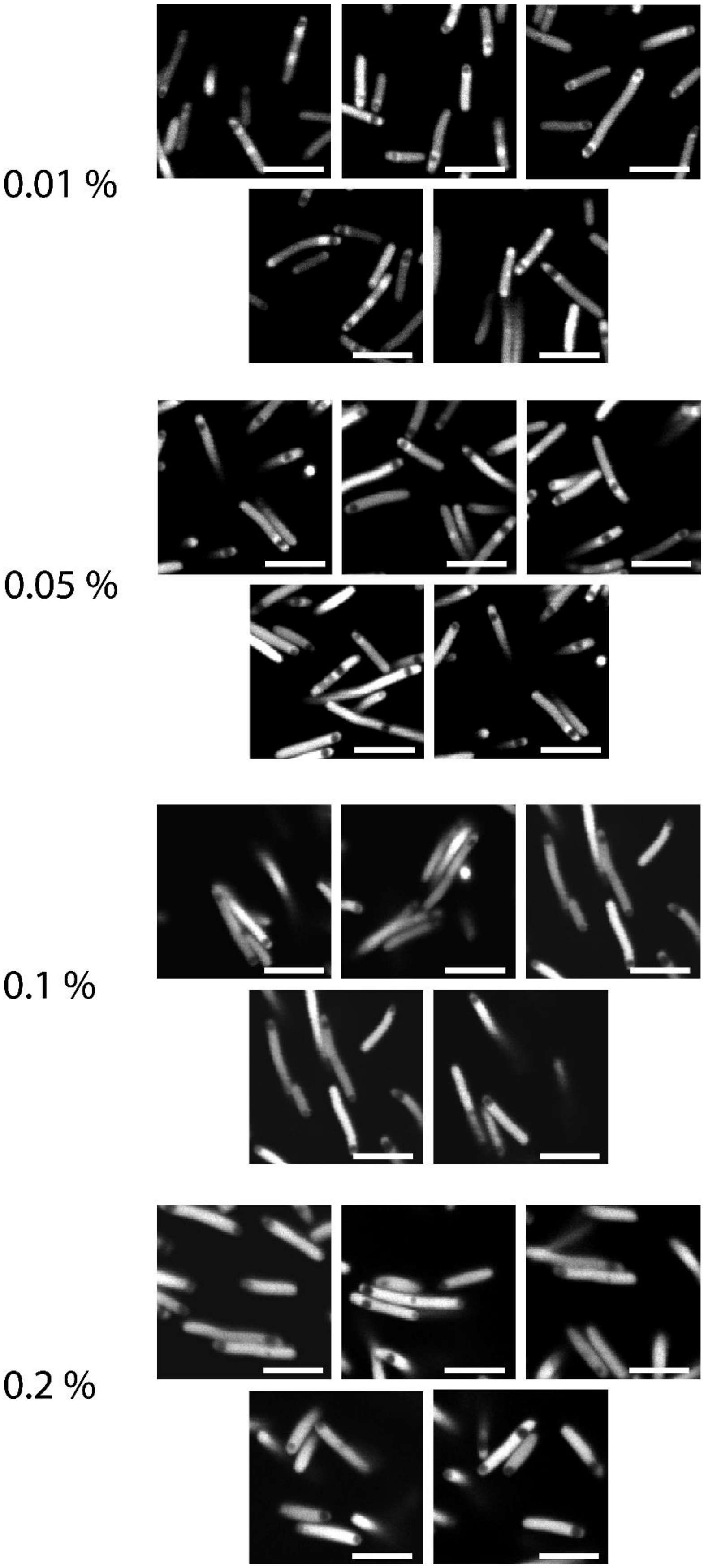

Supplementary Fig. S39: Microscopy of HERD-2.2–GFP with increasing HERD-2.2–FMO and HERD-2.2–TnaA. Cells grown and imaged at 33 °C with varying (0.01% - 0.2% w/v) D-arabinose. Scale bars 5 µm.

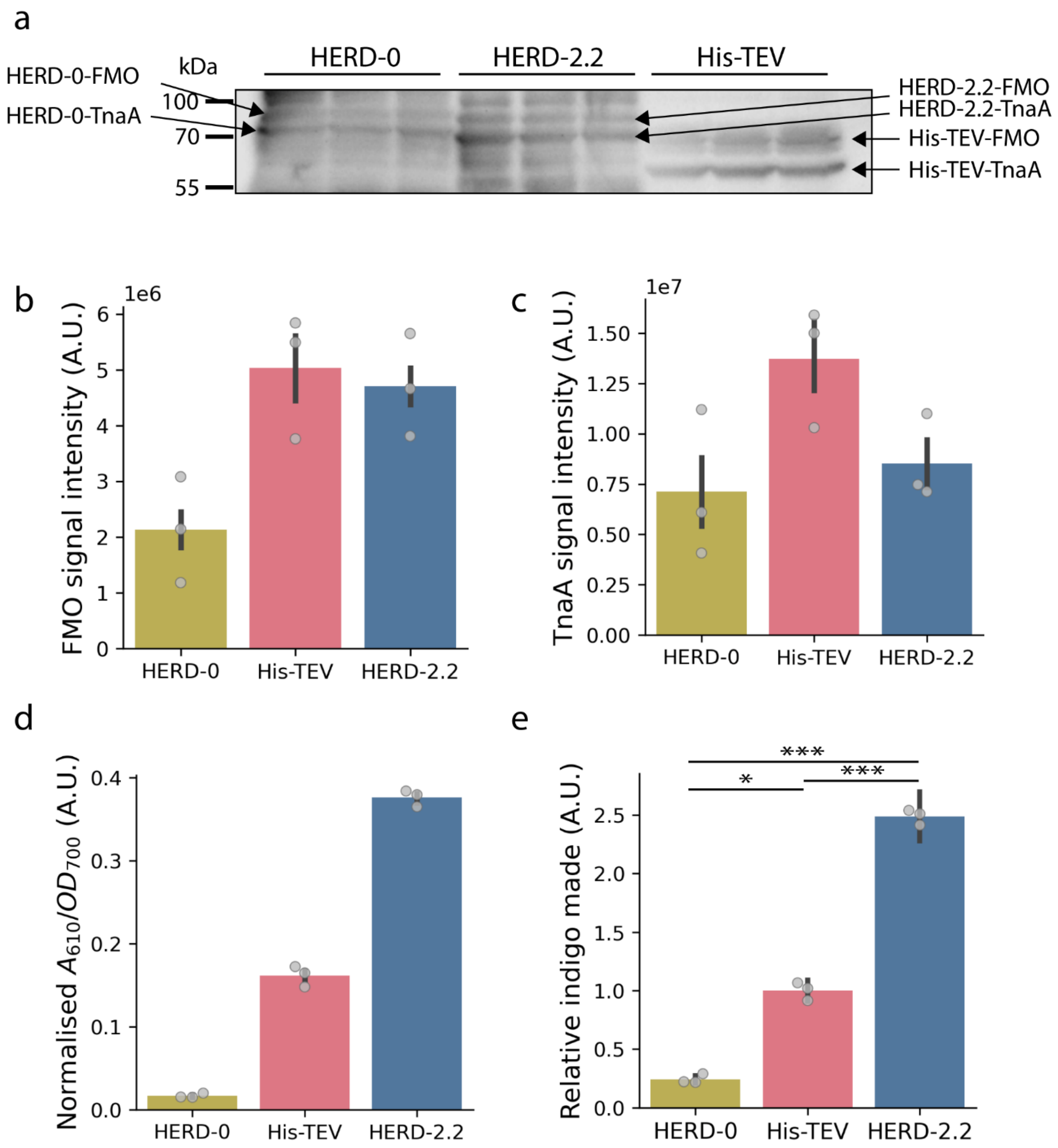

Supplementary Fig. S40: Quantification of enzyme expression and indigo production at 33 °C. a, Western blot for recombinantly expressed FMO and TnaA fusions (transferred from a 12% acrylamide/bis-acrylamide SDS-PAGE gel). HERD-0 and His-TEV fusions were expressed with 0.2% D-arabinose, and HERD-2.2 fusions with 0.01%. **b, c,** Quantification of FMO and TnaA protein expression from western blotting. Quantification performed using Image Studio Lite with upper and lower median background subtraction to normalise for background signal. Data are represented as the mean +/- the standard error from n = 3 biologically independent experiments for each sample. **d,** Quantification of indigo produced (A_{610}), normalised by cell density (OD_{700}) to give the raw amount of indigo produced per culture. Data are represented as the mean +/- the standard error from n = 3 biologically independent experiments for each sample. **e,** Amount of indigo produced normalised both by cell density and by FMO expression levels to give the relative amount of indigo made. Data are represented as the mean +/- the standard error from n = 3 biologically independent experiments for each sample. Mean and standard deviation for each sample: His-TEV mean = 1.00, standard deviation = 0.19; HERD-0 mean = 0.24, standard deviation = 0.09; HERD-2.2 mean = 2.49, standard deviation = 0.40. HERD-0 vs His-TEV P=0.0285 (*); His-TEV vs HERD-2.2 P=0.001 (***); HERD-0 vs HERD-2.2 P=0.001 (***) by one-way ANOVA and Tukey HSD post-hoc test.

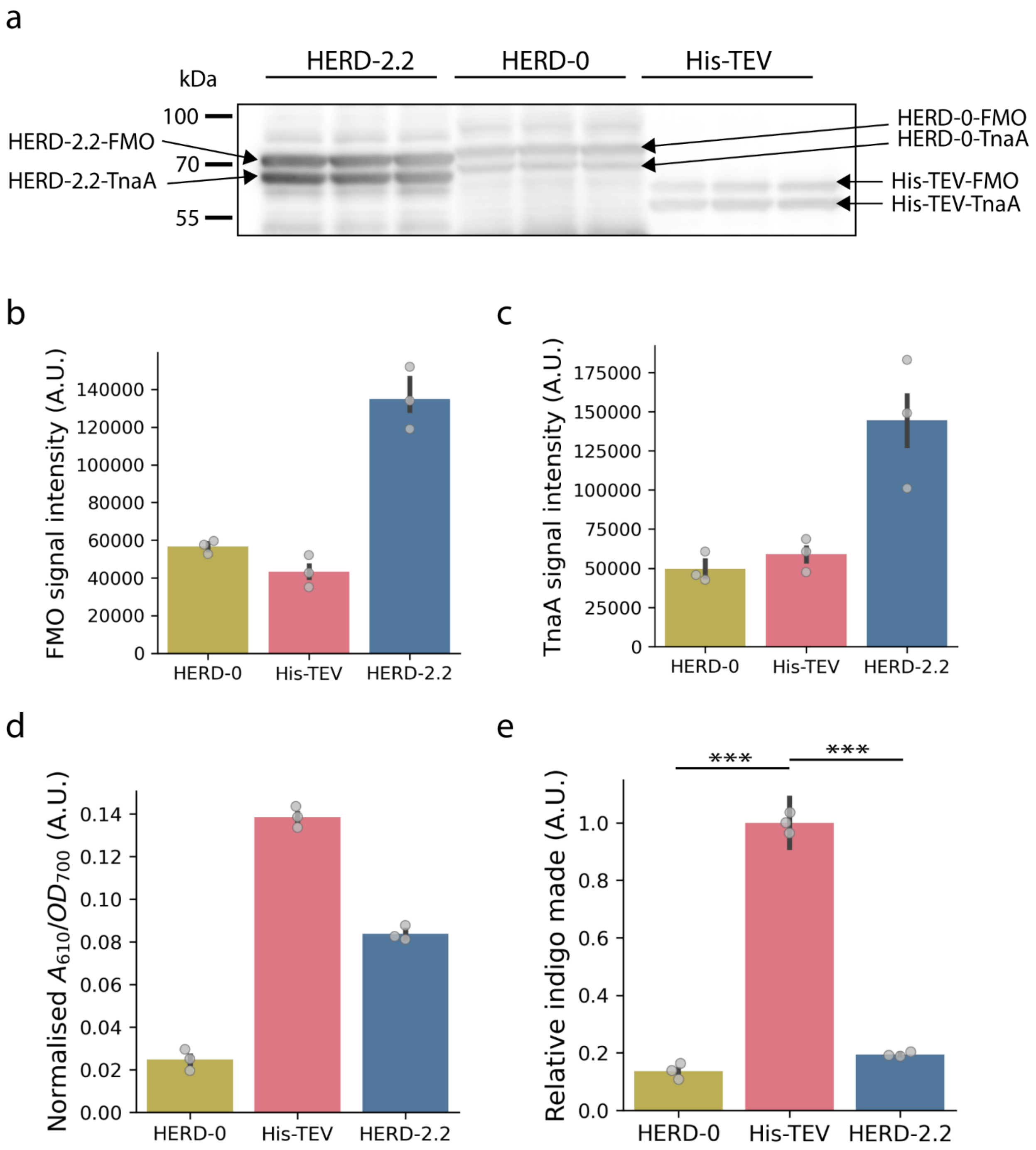

Supplementary Fig. S41: Quantification of enzyme expression and indigo production at 18 °C. a, Western blot for recombinantly expressed FMO and TnaA fusions (transferred from a 12% acrylamide/bis-acrylamide SDS-PAGE gel). HERD-0, HERD-2.2, and His-TEV fusions were expressed with 0.2% D-arabinose. **b, c,** Quantification of FMO and TnaA protein expression from western blotting. Quantification performed using Image Studio Lite with upper and lower median background subtraction to normalise for background signal. Data are represented as the mean +/- the standard error from n = 3 biologically independent experiments for each sample. **d,** Quantification of indigo produced (A_{610}), normalised by cell density (OD_{700}) to give the raw amount of indigo produced per culture. Data are represented as the mean +/- the standard error from n = 3 biologically independent experiments for each sample. **e,** Amount of indigo produced normalised both by cell density and by FMO expression levels to give the relative amount of indigo made. Data are represented as the mean +/- the standard error from n = 3 biologically independent experiments for each sample. Mean and standard deviation for each sample: His-TEV mean = 1.00, standard deviation = 0.09; HERD-0 mean = 0.14, standard deviation = 0.01; HERD-2.2 mean = 0.19, standard deviation = 0.01. HERD-0 vs His-TEV P = 0.001 (***); His-TEV vs HERD-2.2 P = 0.001 (***) by one-way ANOVA and Tukey HSD post-hoc test.

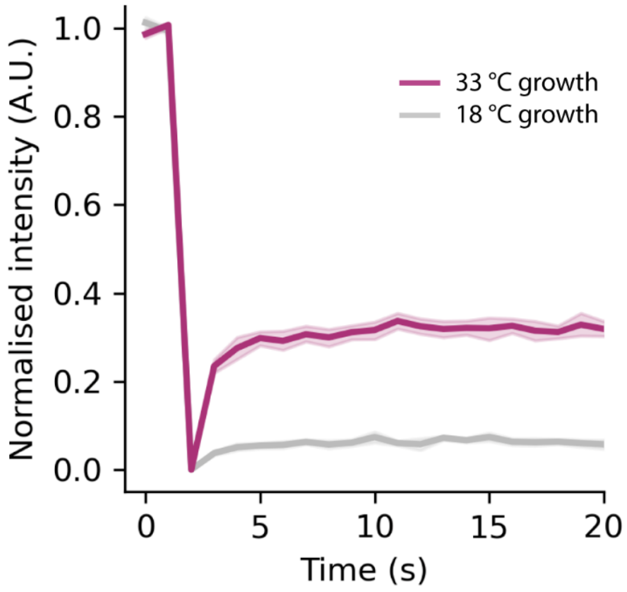

Supplementary Fig. S42: FRAP of HERD-2.2-GFP droplets in cells under different growth conditions. Fluorescence recovery of bleached spots within HERD-2.2–GFP protein condensates in *E. coli* grown at 33 °C (purple) and 18 °C (grey) after induction. Shaded area represents the standard error. Data are represented as the mean +/- the standard error from n=16 (33 °C) and n=15 (18 °C) biologically independent experiments.

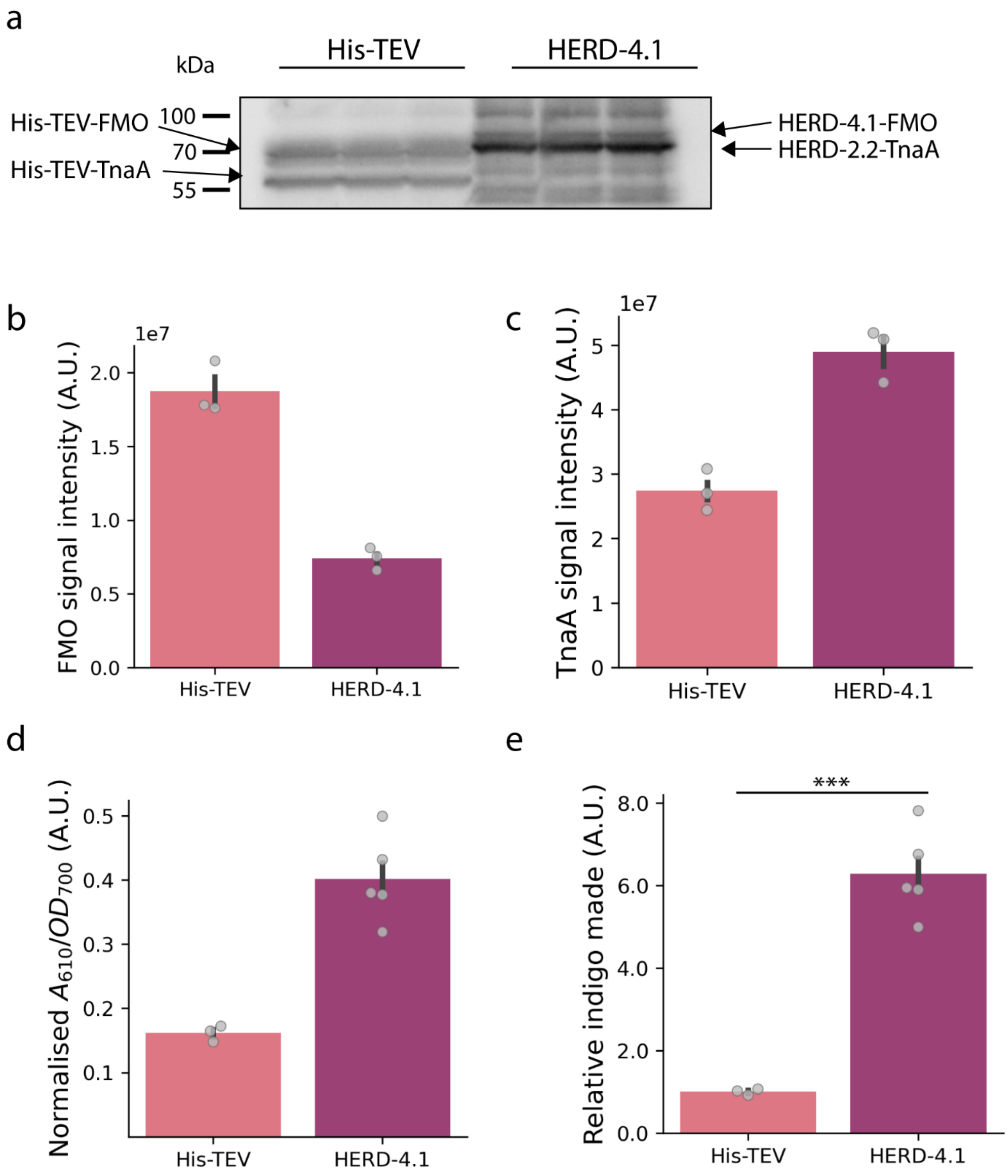

Supplementary Fig. S43: Quantification of enzyme expression and indigo production. **a,** Western blot for recombinantly expressed FMO and TnaA fusions (transferred from a 12% acrylamide/bis-acrylamide SDS-PAGE gel). His-TEV fusions were expressed with 0.2% D-arabinose, and HERD-4.1 fusions with 0.01%. **b, c,** Quantification of FMO and TnaA protein expression from western blotting. Quantification performed using Image Studio Lite with upper and lower median background subtraction to normalise for background signal. Data are represented as the mean +/- the standard error from n = 3 biologically independent experiments for each sample. **d,** Quantification of indigo produced (A_{610}), normalised by cell density (OD_{700}) to give the raw amount of indigo produced per culture. Data are represented as the mean +/- the standard error from n = 3 (His-TEV) or n = 5 (HERD-4.1) biologically independent samples. **e,** Amount of indigo produced normalised both by cell density and by FMO expression levels to give the relative amount of indigo made. Data are represented as the mean +/- the standard error from n = 3 (His-TEV) or n = 5 (HERD-4.1) biologically independent samples. Mean and standard deviation for each sample: His-TEV mean = 1.00, standard deviation = 0.05; HERD-4.1 mean = 6.28, standard deviation = 1.09. His-TEV vs HERD-4.1 P = 0.001 (***) by one-way ANOVA and Tukey HSD post-hoc text.

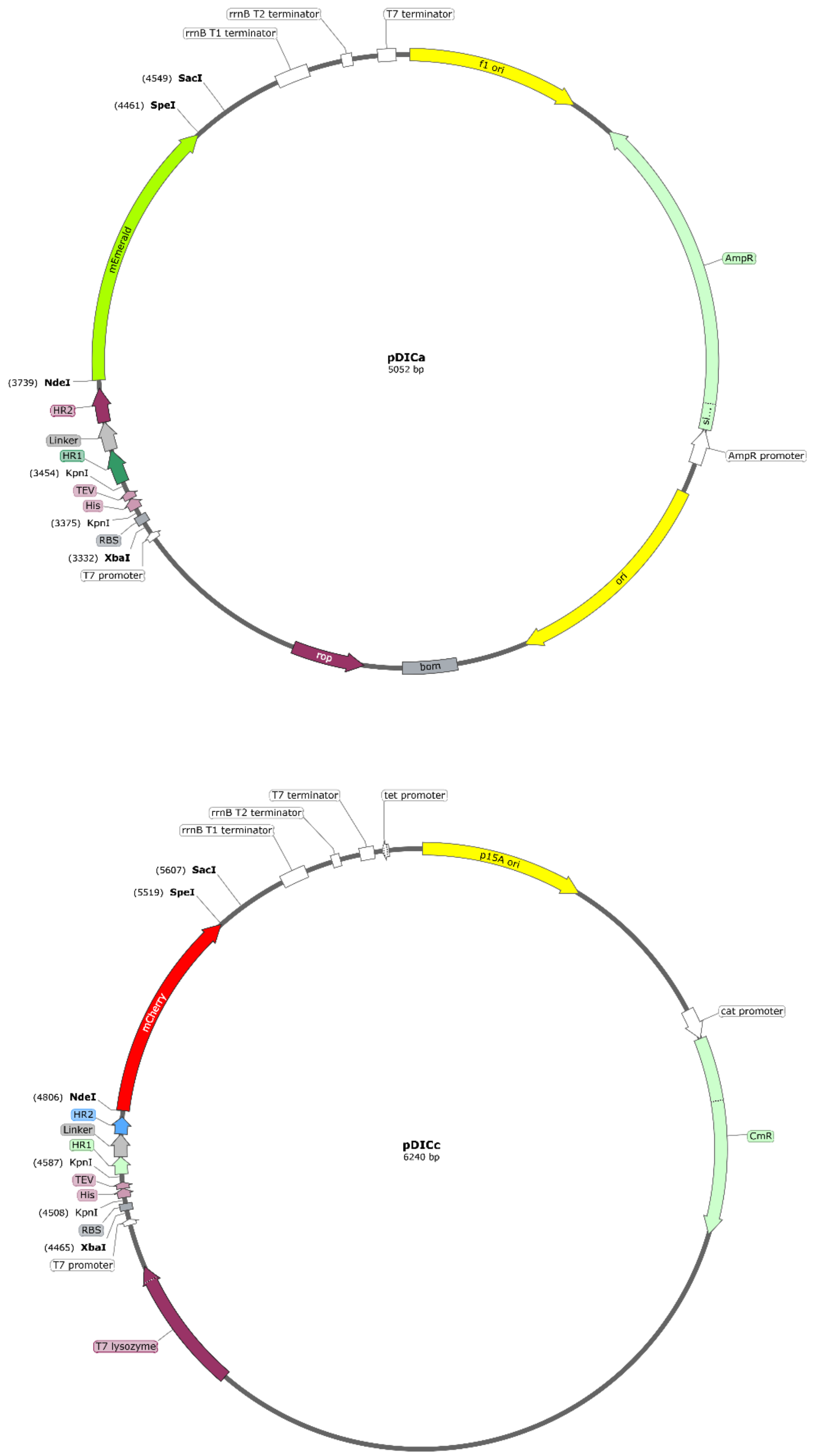

Supplementary Fig. S44: pDIC (design in cells) vectors used for protein expression. Upper – pDICa (ampicillin selection). Lower – pDICc (chloramphenicol selection).

Unprocessed scans of blots and gels for supplementary figures

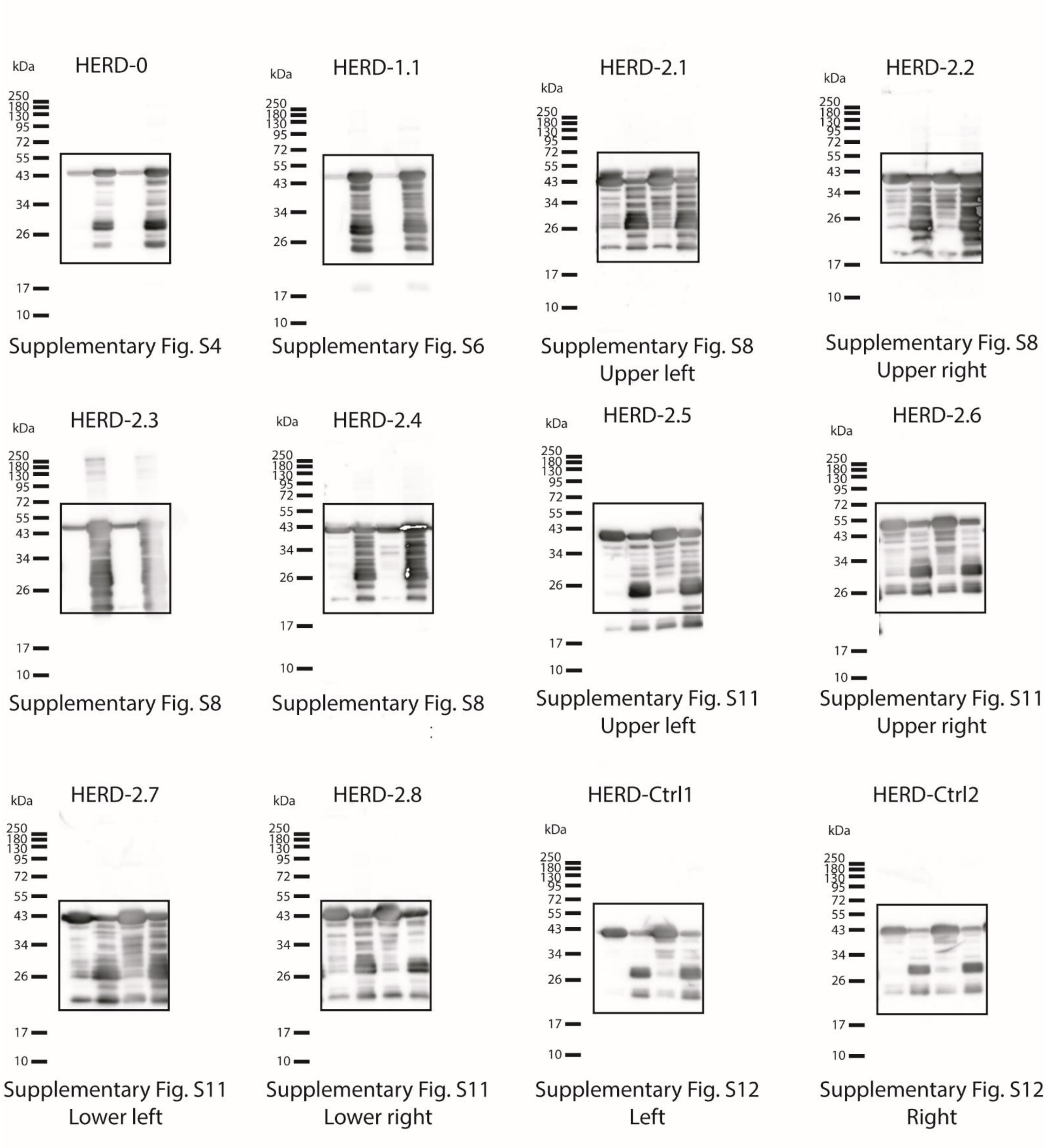

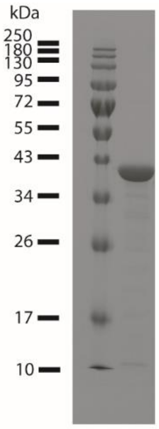

Supplementary Fig. S17

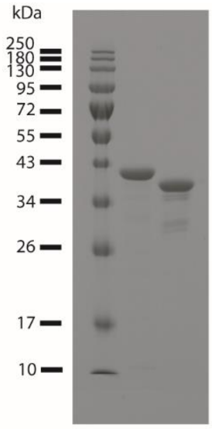

Supplementary Fig. S21

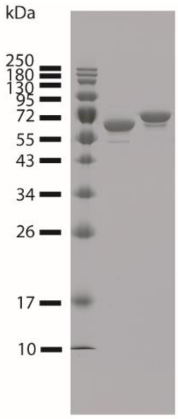

Supplementary Fig. S33

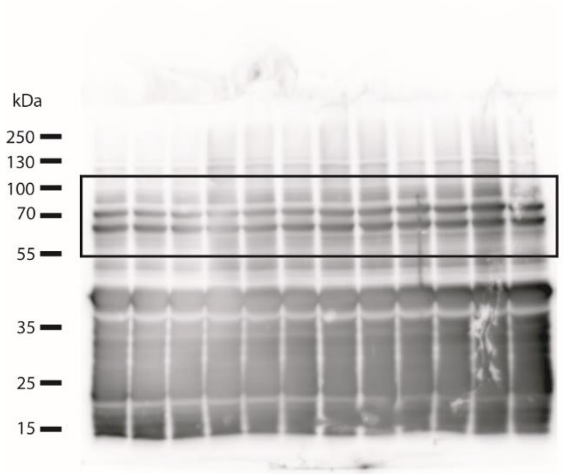

Supplementary Fig. S38a

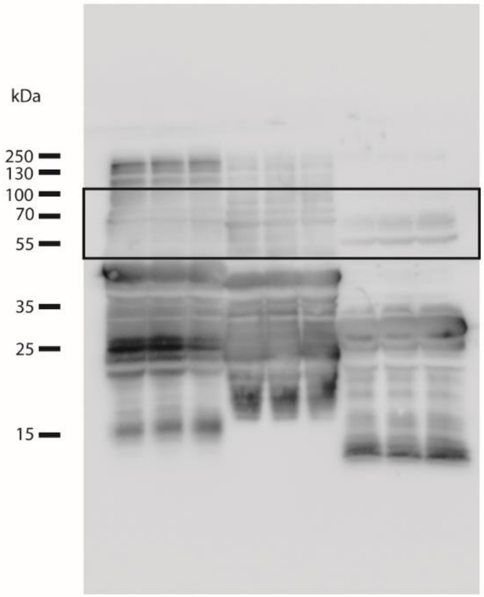

Supplementary Fig. S40a

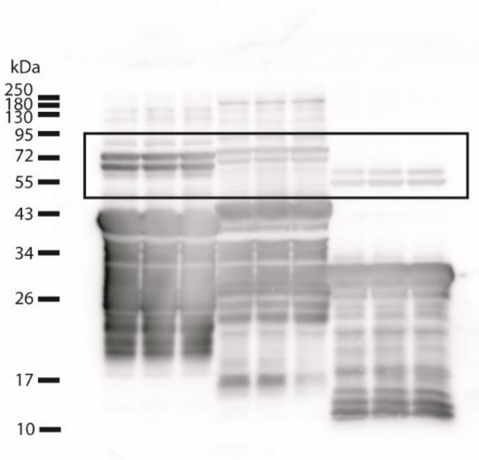

Supplementary Fig. S41a

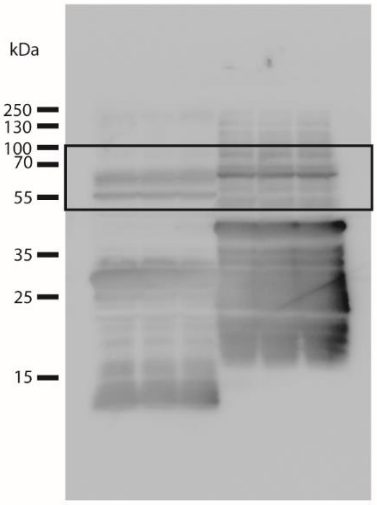

Supplementary Fig. S43a
