# Supplementary material for: Assembling membraneless organelles from de novo designed proteins
Source: Nat Chem. 2023 Sep 14;16(1):89–97. doi: 10.1038/s41557-023-01321-y (PMC10774119; doi:10.1038/s41557-023-01321-y)
Supplement: Supplementary file 2 — Reporting Summary [file 41557_2023_1321_MOESM2_ESM.pdf]

nature portfolio

Corresponding author(s): Richard Obexer, Jennifer J. McManus, & Derek N. Woolfson

Last updated by author(s): Jun 29, 2023

Reporting Summary

Nature Portfolio wishes to improve the reproducibility of the work that we publish. This form provides structure for consistency and transparency in reporting. For further information on Nature Portfolio policies, see our Editorial Policies and the Editorial Policy Checklist.

Statistics

For all statistical analyses, confirm that the following items are present in the figure legend, table legend, main text, or Methods section.

n/a	Confirmed	
☐	☒	The exact sample size (*n*) for each experimental group/condition, given as a discrete number and unit of measurement
☐	☒	A statement on whether measurements were taken from distinct samples or whether the same sample was measured repeatedly
☐	☒	The statistical test(s) used AND whether they are one- or two-sided<br>*Only common tests should be described solely by name; describe more complex techniques in the Methods section.*
☒	☐	A description of all covariates tested
☒	☐	A description of any assumptions or corrections, such as tests of normality and adjustment for multiple comparisons
☐	☒	A full description of the statistical parameters including central tendency (e.g. means) or other basic estimates (e.g. regression coefficient) AND variation (e.g. standard deviation) or associated estimates of uncertainty (e.g. confidence intervals)
☐	☒	For null hypothesis testing, the test statistic (e.g. *F*, *t*, *r*) with confidence intervals, effect sizes, degrees of freedom and *P* value noted<br>*Give P values as exact values whenever suitable.*
☒	☐	For Bayesian analysis, information on the choice of priors and Markov chain Monte Carlo settings
☒	☐	For hierarchical and complex designs, identification of the appropriate level for tests and full reporting of outcomes
☒	☐	Estimates of effect sizes (e.g. Cohen's *d*, Pearson's *r*), indicating how they were calculated

Our web collection on statistics for biologists contains articles on many of the points above.

Software and code

Policy information about availability of computer code

Data collection	Data were collected using the following software: Circular dichroism - Spectra Manager (1.55); Microscopy image acquisition - LAS_X_Core_3.7.6_25997; Cloud point acquisition - UVWinLab (5.1); Dynamic light scattering acquisition - ALV for Windows (3.04.11)
Data analysis	Microscopy image analysis was performed using ImageJ (1.53f51). Volume fraction calculations used Sedfit (16.1). Code for the foci detection software Modular Image Analysis (1.0.3) is available at the Zenodo repositories: https://doi.org/10.5281/zenodo.6832092 and https://doi:10.5281/zenodo.6907671 and used the MIA ImageJ plugin (0.21.11). Fitting of photobleaching data to exponential functions was performed using OriginPro (2021b). All other data were analysed using Python (3.8.5), matplotlib (3.3.2), pandas (1.1.3), and numpy (1.19.2).

For manuscripts utilizing custom algorithms or software that are central to the research but not yet described in published literature, software must be made available to editors and reviewers. We strongly encourage code deposition in a community repository (e.g. GitHub). See the Nature Portfolio guidelines for submitting code & software for further information.

Data

Policy information about availability of data

All manuscripts must include a data availability statement. This statement should provide the following information, where applicable:

- Accession codes, unique identifiers, or web links for publicly available datasets
- A description of any restrictions on data availability
- For clinical datasets or third party data, please ensure that the statement adheres to our policy

All raw data associated with this manuscript have been deposited in the following Zenodo repository: https://doi.org/10.5281/zenodo.7199035. The DeepBacs E. coli dataset is available in the Zenodo repository: https://doi.org/10.5281/zenodo.5550935.

Human research participants

Policy information about studies involving human research participants and Sex and Gender in Research.

Reporting on sex and gender	N/A
Population characteristics	N/A
Recruitment	N/A
Ethics oversight	N/A

Note that full information on the approval of the study protocol must also be provided in the manuscript.

Field-specific reporting

Please select the one below that is the best fit for your research. If you are not sure, read the appropriate sections before making your selection.

☒ Life sciences ☐ Behavioural & social sciences ☐ Ecological, evolutionary & environmental sciences

For a reference copy of the document with all sections, see nature.com/documents/nr-reporting-summary-flat.pdf

Life sciences study design

All studies must disclose on these points even when the disclosure is negative.

Sample size	Biophysical measurements do not have sample sizes but were validated by replication as described below. For detection of foci within E. coli, cells were detected from at least 18 images generating sample sizes of at least 4000 cells per condition. For fluorescence recovery after photobleaching, sample sizes of at least 10 independent measurements per sample were shown to be sufficient due to the low variation between measurements. For quantification of small molecule labeling (ReAsH) in droplets, sample sizes of 8-12 were used, and shown to be sufficient, with one-way ANOVA giving a P value of <0.001 for all cross comparisions. For quantification of indigo production in E. coli, each condition was performed in triplicate independent measurements which was sufficient, giving a P value of <0.001 by one-way ANOVA and low variance about the mean for each condition. This sample size is consistent with previous studies, e.g.: https://doi.org/10.1021/acssynbio.6b00141
Data exclusions	No data was excluded from this study.
Replication	All attempts at replication of the experiments in this study were successful, and mean and variance values were generated from at least 3 independent measurements in all cases.
Randomization	This study did not involve samples being allocated into experimental groups, and therefore statistical hypothesis issues related to randomisation do not apply to this study.
Blinding	This study does not involve experiments where the outcome would be influenced by blinding, and therefore statistical hypothesis issues related to blinding do not apply to this study.

Reporting for specific materials, systems and methods

We require information from authors about some types of materials, experimental systems and methods used in many studies. Here, indicate whether each material, system or method listed is relevant to your study. If you are not sure if a list item applies to your research, read the appropriate section before selecting a response.

Materials & experimental systems

n/a	Involved in the study
☐	☒ Antibodies
☒	☐ Eukaryotic cell lines
☒	☐ Palaeontology and archaeology
☒	☐ Animals and other organisms
☒	☐ Clinical data
☒	☐ Dual use research of concern

Methods

n/a	Involved in the study
☒	☐ ChIP-seq
☒	☐ Flow cytometry
☒	☐ MRI-based neuroimaging

Antibodies

Antibodies used	Monoclonal anti-polyhistidine antibody produced in mouse (H1029, clone HIS-1), Sigma Aldrich: https://www.sigmaaldrich.com/GB/en/product/sigma/h1029. Goat anti-mouse IgG (H+L) secondary antibody HRP (31430), Invitrogen: https://www.thermofisher.com/antibody/product/Goat-anti-Mouse-IgG-H-L-Secondary-Antibody-Polyclonal/31430.
Validation	Anti-polyhistidine antibodies have been used extensively in E. coli for specific detection of tagged proteins by western blotting, and data are consistent with previous studies, e.g.: https://doi.org/10.7554/eLife.54983, https://doi.org/10.1126/science.1195691, https://doi.org/10.1038/s41586-020-03056-z, and https://doi.org/10.1002/btpr.2227. Both H1029 and 31430 antibodies have been used by the scientific community for western blotting, having 428 and 1509 citations respectively.
